# Flower Conspicuousness to Bees Across Pollination Systems: A Generalized Test of the Bee-Avoidance Hypothesis

**DOI:** 10.3389/fpls.2020.558684

**Published:** 2020-09-24

**Authors:** Gabriel Coimbra, Carina Araujo, Pedro J. Bergamo, Leandro Freitas, Miguel A. Rodríguez-Gironés

**Affiliations:** ^1^ Jardim Botânico do Rio de Janeiro, Rio de Janeiro, Brazil; ^2^ Estación Experimental de Zonas Áridas, Almería, Spain

**Keywords:** pollination, bee avoidance, flower color, plant-animal communication, antagonism, flower depth, contrast, purity

## Abstract

Flower signals of bee- and bird-pollinated plants have converged *via* pollinator-mediated evolution, driven by the visual system of their respective pollinators. For bird flowers, sensory exclusion of less effective bees is also important and such exclusion is also mediated by floral morphological filters. Likewise, other systems based on pollination by red-sensitive insects are also associated with red flowers displaying lower short-wavelength secondary peaks of reflectance, which decreases detectability to animals that are less sensitive to red, such as bees. These flowers often also present long tubes. Here, we tested a generalization of the bee-avoidance hypothesis in order to assess if it holds only for bird flowers or for other non-bee pollination systems as well. For this, we compared flower contrasts and spectral purity in bee visual systems as proxies for conspicuousness among four kinds of pollination systems: bee-visited flowers, insect-visited flowers (including bees and other insects), non-bee insect flowers (flowers visited by red-sensitive insects such as flies, butterflies and beetles, but not bees), and bird-visited flowers. We also assessed the association between conspicuousness to bees and flower depth, used as a proxy for morphological exclusion of bees. Overall, flower conspicuousness to bees differed only between insect (all three groups) and bird flowers, due to lower visual signals for the latter. This suggests that bee sensory exclusion *via* color signals is exclusive to bird flowers, while non-bee insect flowers might use other sensory channels to exclude bees, such as olfactory signals. Visual bee avoidance might be a mechanism exclusive to plants pollinated by specific guilds of red-sensitive insects not well represented in our sample. We also found a negative association between flower conspicuousness to bees and flower depth, suggesting an interplay of morphological and spectral traits in discouraging bee visits. Our results support the bee-avoidance hypothesis exclusively for bird flowers and an overall association between lower visual signals to bees and long tubes.

## Introduction

Pollinator-mediated evolution of flower traits has been shown to be a major factor shaping angiosperm diversity [e.g., Schemske and Bradshaw (1999); Schiestl and Johnson (2013)]. Each pollinator group (e.g., bees, flies, and butterflies) responds to signals according to its, innate or not, preferences and sensorial skills, driving convergent flower evolution (Schiestl and Johnson, 2013). Floral color signals are expected to converge when sharing a specific guild of pollinators, such as UV-reflection gradients in bee flowers (Papiorek et al., 2016) and red reflection in bird flowers (Burd et al., 2014). Moreover, floral colors have often evolved in multiple-receiver contexts (Renoult et al., 2014). Nevertheless, consideration of the distinct pressures driven by multiple receivers, including less effective floral visitors, has been overlooked. Therefore, the contribution of pollinator preferences driving floral signal evolution may be overestimated in relation to selective pressures exerted by other visitors. Broad-scale comparisons of floral color considering less effective floral visitors may reveal unnoticed signaling patterns, with implications to the understanding of flower trait evolution.

Most bee flowers present inflection points (parts of rapidly changing reflectance that are optimally detected by visual systems in general) at regions of the spectrum of maximum discrimination for Hymenopteran vision [Chittka (1992); Dyer et al. (2012)], indicating a loose signal-receptor match that enhances detectability by bees. The same match has been found for bird-flower signals and bird vision (Shrestha et al., 2013). In the case of plants pollinated by hummingbirds, bee vision seems to have played a critical role as well, likely due to negative consequences of bee visitors in bird flowers (Bergamo et al., 2019). Bee pollination is known to precede vertebrate pollination in evolutionary history (Rosas-Guerrero et al., 2014), and bee-bird pollinator shifts may have occurred because of the differential associations these vectors have with pollen: while bees use it as a resource to feed their larvae, birds mostly ignore it (Thomson and Wilson, 2008). Moreover, vertebrate pollinators like birds require more energy and have the potential to transfer pollen at longer distances than bees (Ashworth et al., 2015) presumably because of their increased mobility, size and energy requirements (Rosas-Guerrero et al., 2014). Thus, selective pressures on bird flowers to deter bees from using pollen and depleting the costly, copious nectar required by this new type of pollen vector are expected (Thomson and Wilson, 2008).

The negative effects of bees on bird flowers lays the foundation for the **bee-avoidance hypothesis** (Raven, 1972), which attempts to explain an apparently unreasonable number of red bird flowers. Reddish colors are less detectable by bees, since these insects exhibit low red-wavelength sensitivity (Lunau et al., 2011). As a result, birds would visit more frequently red flowers in order to avoid competition with bees (Rodríguez-Gironés and Santamaría, 2004), the most abundant anthophilous animals (Michener, 2000). Bird flowers, in their turn, were selected to display colors less discriminable by bees, like red, imposing enough additional foraging costs for discouraging bee visits [Raven (1972); Lunau et al. (2011)], even though bees are not completely blind to red light (Chittka and Waser, 1997). In this case, pollinator preference is unlikely because birds visit flowers of other colors (Papiorek et al., 2016) and present no innate preference for red (Lunau et al., 2011). Bird flowers also lack known olfactory signals and present long tubes that may act as filters against bees since foraging costs for these insects increase with flower depth (Harder and Cruzan, 1990). However, it is unclear if flower depth is negatively associated with conspicuousness to bees across the angiosperms. All these traits seem to work synergistically, creating not just filters but a private channel of communication between birds and bee-avoiding, odorless, long-wavelength-reflecting and long-tubed bird flowers [Castellanos et al. (2004); Willmer (2011); Gegear et al. (2017)]. The bee-avoidance hypothesis for bird-pollinated plants has been hitherto supported by case studies [e.g., Bergamo et al. (2016); Gegear et al. (2017)] and broader comparisons between bee and bird flowers [e.g., Lunau et al. (2011); Camargo et al. (2019)].

Bees are the most diverse and abundant group of floral visitors in most ecosystems (Michener, 2000). Therefore, bees could have influenced the evolution of any animal-pollinated system. Little is known about pollinator shifts between insect groups, like bee-fly shifts, or how non-bee insect systems have evolved in the presence of bees. In other words, it is uncertain if visual bee-avoidance mechanisms have evolved in other non-bee systems. For Australian orchids, flower color was found to differ between bee- and fly-pollinated species, the latter having most of their inflection points beyond 500 nm (Shrestha et al., 2019), as found for bee-avoiding bird flowers (Lunau et al., 2011), which is roughly the limit of bee chromatic vision. The same difference was found between flowers of a community with a dipteran-exclusive pollinator fauna in Macquarie Island and their inland bee-pollinated relatives (Shrestha et al., 2016). In both cases, fly flowers seem to be more constrained in bee visual color space and less spectrally diverse than bee-flower color, which seems to be a general feature of these flowers Willmer (2011). Nevertheless, if non-bee insect flowers are less conspicuous to bee vision remains to be tested. Furthermore, red flowers are sometimes associated with other insect pollination systems such as butterfly- [e.g., Johnson and Bond (1994); Willmer (2011)] and beetle-pollinated species (Dafni et al., 1990). These differ from bees in their visual systems presenting a higher red sensitivity, which was demonstrated for some species [e.g., flies: Lunau (2014); beetles, butterflies: Briscoe and Chittka (2001)].

Red flowers pollinated by insects with red receptors have been shown to present lower reflectance intensity of secondary peaks at shorter wavelengths (Chen et al., 2020), which might result in lower contrasts to animals lacking red receptors like bees. Thus, we hypothesize that bee vision might be a factor acting on flower color selection in non-bee insect-pollinated systems, resulting in lower contrasts that might act synergistically with long tubes also found for butterfly- Willmer (2011) and some fly-pollinated (Goldblatt and Manning, 2000) species.

We tested a generalization of the bee-avoidance hypothesis in a broad-scale set of species, using plants spread on the phylogeny of angiosperms, which have evolved in diverse geographical and ecological backgrounds. Using flower reflectance data, we computed three metrics for conspicuousness in the bee visual systems. We then compared the conspicuousness of species visited by bees to those of species visited by other groups to test whether all non-bee-pollinated systems have evolved bee-avoidance mechanisms. We expected lower conspicuousness for species visited by other groups in comparison to species visited by bees (i.e., a general bee-avoidance pattern). We also looked for an association between flower depth and conspicuousness regardless of pollination system, to test if long tubes and low conspicuousness to bees are key traits that act synergistically in long-tubed flowers.

## Material and Methods

### Species Sample and Study Area

Data for 233 out of the total 389 species (see **Table S1** for the complete species list and **Table S2** for data sources) in our sample were downloaded from the Floral Reflectance Database [hereafter “FReD”, Arnold et al. (2008); accessed in August 2019]. We included all species for which reflectance, flower depth and flower visitors’ identity were available. Additionally for these species, leaf reflectance was also downloaded when available. The remaining 156 species were sampled at the Botanical Garden of Rio de Janeiro, Brazil (hereafter “JBRJ”). Flowers were sampled from February 2017 to August 2019 at the arboretum, a live collection house to roughly nine-thousand species in its 54 ha (http://rb.jbrj.gov.br/arboreto/arboretoleaflet.php). For all animal-pollinated species in bloom, at least three samples of the most dominant attractive structure in the display of a given species (see *Spectral Reflectance*) and three leaves were collected according to availability. Following literature research on visitors’ data for each plant species sampled at JBRJ, two final datasets were created. The first one, used for comparisons between pollination systems, comprised 285 species (233 from FReD and 52 from JBRJ). This group of species had available information on flower visitors which fell in the pollination system categories established, thus we excluded species visited by underrepresented groups (see *Pollination Systems*). We also excluded species for which we found evidence of sexual deceit and sapromyophily, since these flower colors evolved in different contexts from those where bee-avoidance mechanisms would be expected. The second dataset, used for assessing the association between bee contrast and flower depth, included all species but those with dish-shaped flowers (see *Flower Depth Measurements*), totaling 286 species (135 from FReD and 151 from JBRJ).

### Spectral Reflectance

Reflectance measurements of each structure (five on average) of species sampled at the Botanical Garden of Rio de Janeiro were taken using a portable spectrometer (USB 4000; Ocean Optics) at an angle of 45°. Barium sulfate (*BaSo*_4_) was used as white standard and a black chamber as black standard [Lunau et al. (2011); Bergamo et al., 2016)]. Only the predominant color in the display of a given species was considered (see **Table S2** for a list of structures). We decided to include structures such as bracts and calyces because bracts were found to play a more important role in bee avoidance than petals in bracted species (Bergamo et al., 2019). We hereafter refer to all structures generally as “flowers”. We restricted the analyses to the 300–700 nm wavelength range, which falls under the spectral sensitivity of animal pollinators.

### Flower Color Categories

**Figure 1:**
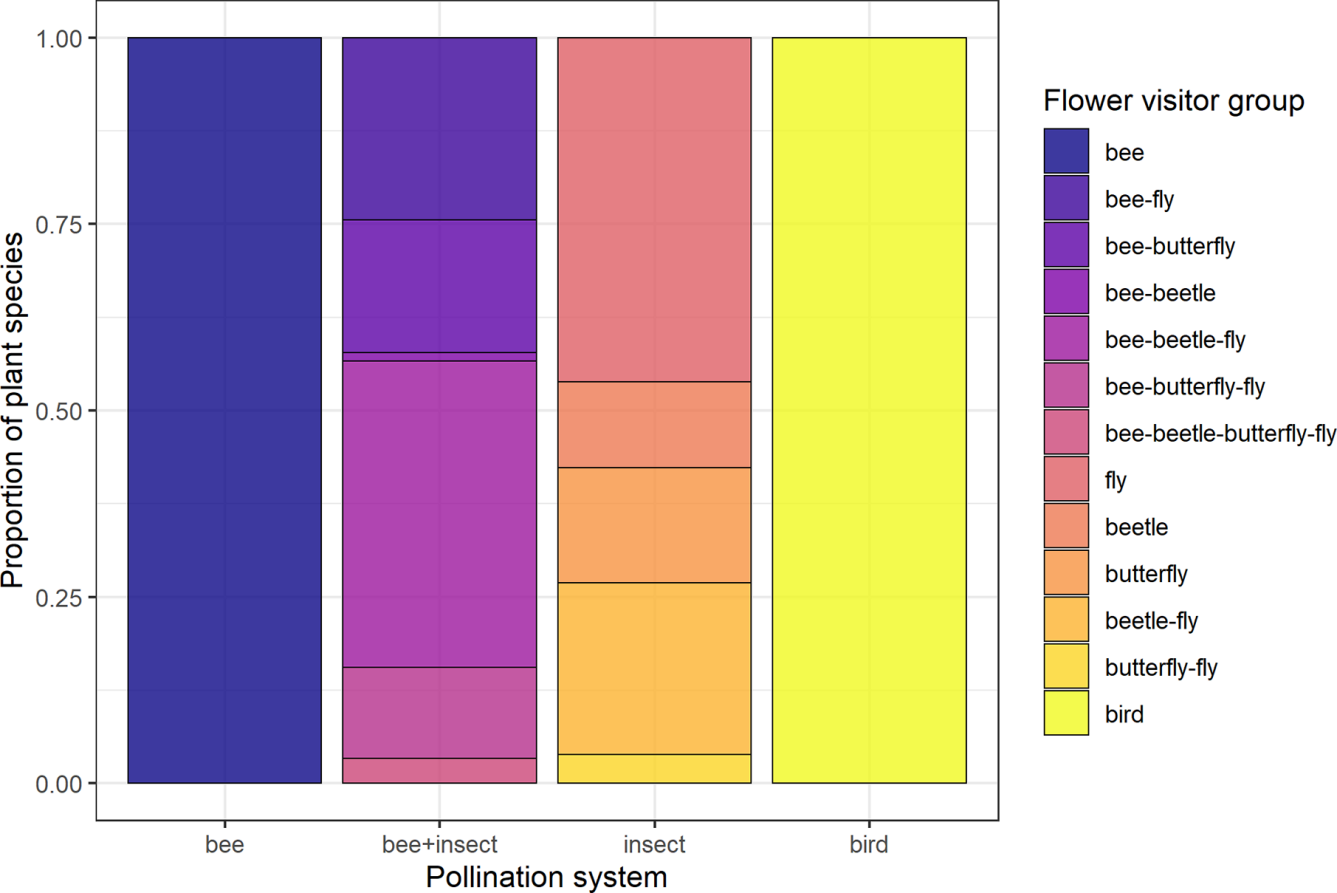
Composition of the four groups of pollination systems designated according to flower visitor identity of each species. Groups are: “Bee” (species visited solely by bees, *N* = 137), “bee+insect” (species visited by both bees and other insects, *N* = 90), “insect” (non-bee insect flowers, i.e., species visited by potentially red-sensitive insects, but not by bees; *N* = 26) and “bird” (species visited solely by birds, *N* = 32).

We classified flower reflectance spectra of each species into color categories based on their average reflectances in the UV, blue, green, and red wavebands, following Camargo et al. (2019) and Chittka et al. (1994) but modifying the thresholds between absorbance and reflectance according to the distribution of our data (**Figure 1**, see **Table S2** for mean intensity values in each band and **Figure S1** for the mean reflectance curve of the whole dataset). In order to discriminate absorbance (−) from reflectance (+) in a given waveband, we used as thresholds 10% for the UV, 30% for the blue, 40% for the green, and 60% for the red bands. Additionally, we classified flowers reflecting in the green band with a difference ≥ 50% in relation to the mean reflection in the blue/red bands as green-absorbing [adapted from Camargo et al. (2019), see **Figure S2** for the mean reflectance curves of each color category].

### Flower Depth Measurements

The total flower depth of on average five (but at least three) flowers of the species sampled at the Botanical Garden of Rio de Janeiro was measured with a digital caliper. For all other species, data were extracted directly from FReD [Arnold et al. (2008); accessed in August 2019]. We included in our dataset for flower depth analysis species for which flower visitors were unknown. When flower visitors’ identity was available, however, we excluded species whose visitors did not fall into the categories established for comparisons across pollination systems (see *Pollination Systems*). Since we aimed at investigating the interplay between two bee-avoiding traits, namely long flower tubes and low conspicuousness, we excluded dish-shaped flowers from this analysis, in practice considering only species with flower *depth* ≥ 1 mm. We then applied log scale for normalization of flower depth data.

### Pollination Systems

**Figure 2:**
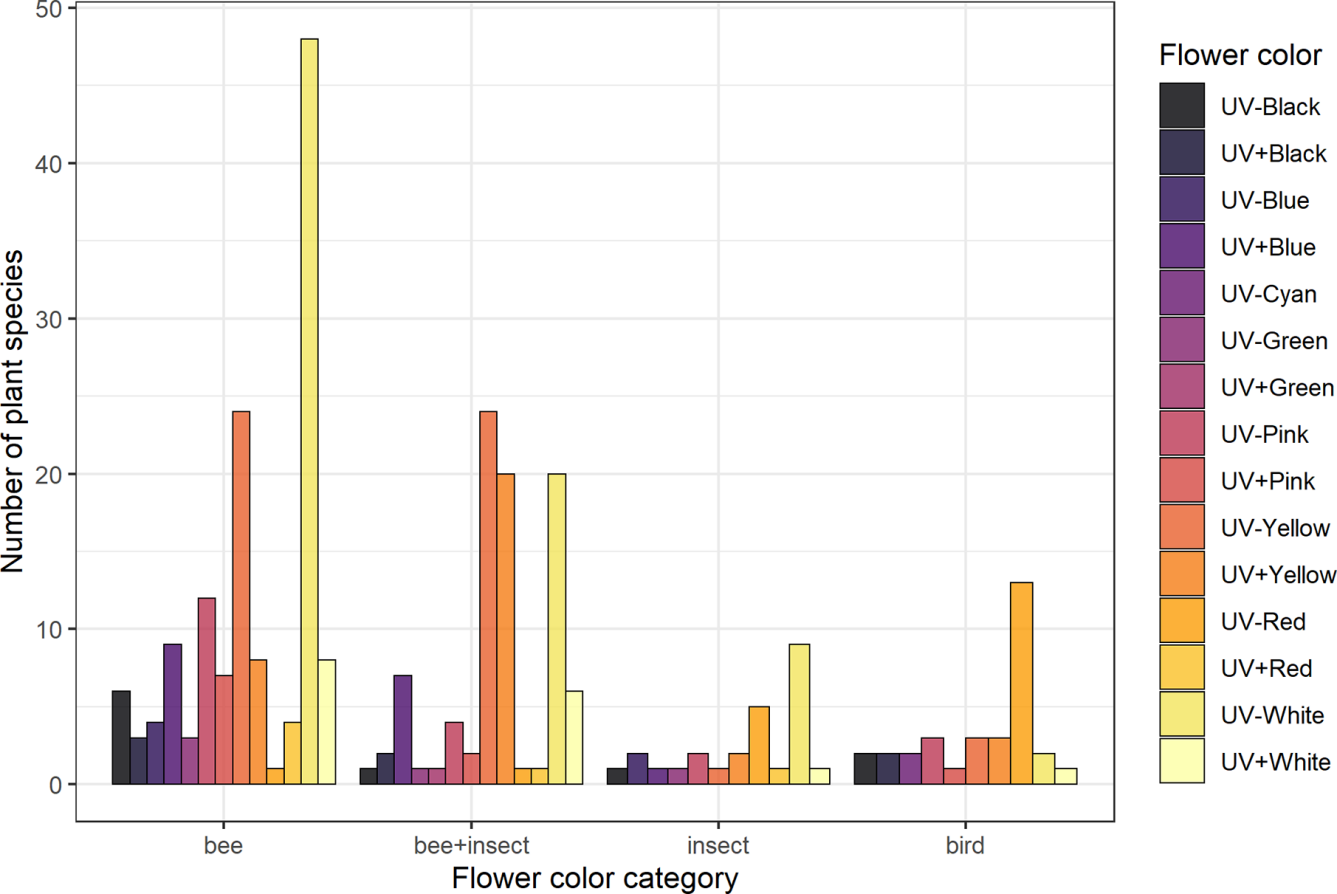
Frequency of flower color categories for each pollination system considered in the analyses. Groups are: “Bee” (species visited solely by bees, *N* = 137), “bee+insect” (species visited by both bees and other insects, *N* = 90), “insect” (non-bee insect flowers, i.e., species visited by potentially red-sensitive insects, but not by bees; *N* = 26) and “bird” (species visited solely by birds, *N* = 32). Flower color categorization was done by averaging reflectance intensity across four bands of the spectrum: UV (from 201 to 300 nm), blue (301–400 nm), green (401–500 nm), and red (501–600 nm) and then assigning either “absorbing” (−) or “reflecting” (+) for each band according to thresholds selected based on the distribution of the data. See *Flower Color Categories* and **Table S2** for details.

Data for floral visitors of each species sampled at JBRJ were obtained from the literature using as key words “pollinat* + [name of the species]”. Since these sources followed different methodologies, we considered these data at the visitor level as potential pollinators. We did not take into account any morphological features (i.e., pollination syndromes). We acknowledge the limitations of using visitors’ data rather than pollination effectiveness for defining groups. However, since our main focus was on the role of antagonists in trait selection, we believe this is unlikely to affect our results. Thus, four groups of species were designated according to their visitors’ identity as distinct pollination systems (**Figure 2**, see **Table S3**): bee flowers (137 species visited solely by bees), bee+insect flowers (90 species visited by bees and other insects), non-bee insect flowers (26 species visited by potentially red-sensitive insects, especially flies but also beetles and butterflies or any combination between them; see **Figure 2**) and bird flowers (32 species visited solely by birds). Thus, we have included both functionally specialized (pollinated solely by one animal group) and generalized pollination systems [*sensu* Ollerton et al. (2007)].

### Color Conspicuousness to Bee Vision

Using the mean reflectance curves of each species, three parameters of color conspicuousness to bees were calculated: achromatic contrast against the background (*ACB* hereafter), chromatic contrast against the background (*CCB*) and spectral purity (*SP*). These were computed according to photoreceptor spectral sensitivities available for the model bee species *Apis mellifera* L. and *Bombus terrestris* L. (Peitsch et al., 1992). We chose those species because honeybees and bumblebees are important pollinators in several ecosystems (Michener, 2000).

We defined *CCB* as the distance between the loci of the flower and the background (Rohde et al., 2013) in the color hexagon of Chittka (1992) and *ACB* (or “green contrast”) as the contrast produced by the green photoreceptor between the stimulus and the background (Rohde et al., 2013). Both of these metrics are of importance because bees use chromatic cues at shorter distances and achromatic cues at longer ones, depending on visual angle (Spaethe et al., 2001). Finally, *SP* refers to the saturation of a given color and is relevant because bees have been shown to prefer colors of high spectral purity when foraging [Lunau et al. (1996); Rohde et al. (2013)]. We used the mean reflectance of all leaves in our sample as the standard leaf background for all species [following Renoult et al. (2015), see **Figure S1** for the mean leaf reflectance used and **Table S4** for all contrast values] and a standard daylight function (D65 irradiance function) as illuminant in the vision models. Using an alternative forest-shade illuminance function did not qualitatively affect our results because we used von Kries correction, which assumes that bee receptors adapt to these changes in illuminant. Even though we recognize the limitations of our standardized approach for species that evolved in diverse illuminants backgrounds, we note that common parameters are necessary for broad-scale comparisons since no specific background and illuminance reflectances were available for each plant species in our dataset. All visual modeling was done with the “pavo” package (Maia et al., 2019) in R software (R Core Team, 2013).

### Statistical Analyses

In order to assess the sensorial exclusion of bees in non-bee flowers, we compared mean contrasts (*ACB* and *CCB*) of bee flowers to those of flowers visited by other vectors, i.e., different pollination systems (bee+insect; non-bee insect, and bird flowers). We ran separate ANOVA tests each using a contrast measurement as the response variable and pollination systems as the explanatory one (four models in total: *ACB* and *CCB* for both *Apis* and *Bombus* visual systems). Then, we computed *post-hoc* Tukey HSD tests to identify the pairs of significant differences between the four kinds of pollination system.

For the analysis of association between flower depth and bee contrast, we fitted four separate linear regressions using flower depth as the explanatory variable and each of the contrast measurements as response variables (Bergamo et al., 2019). Log scale was applied to flower depth for normalization.

To verify if bee contrasts against the background (*ACB* and *CCB*) would be meaningful even against a background of high purity, we verified their relationship with spectral purity (*SP*) in a linear regression, which showed a positive association (**Figure S3**).

In order to account for phylogenetic signal in our sample, we built a phylogenetic hypothesis using the PhytoPhylo megaphylogeny [Qian and Jin (2016) modified from Zanne et al. (2014)] in R for the full dataset (comprising all 389 species). Three different trees for different scenarios were generated, according to choices as to how to insert the branches not found in the megaphylogeny [see Qian and Jin (2016) for details]. The phylogenetic signal of all contrast variables and of flower depth were calculated as Blomberg’s K (Blomberg et al., 2003) using the *phylosig()* function of the “phytools” R-package (Revell, 2012). Values of *K* < 1 indicate that there is no or little phylogenetic signal for that trait (Blomberg et al., 2003). None of the contrasts nor flower depth showed evidence for phylogenetic signal (K close to 0 in all cases, range 0.01–0.09; see **Table S5**).

## Results

Visual modeling of spectral reflectance data yielded *ACB* values ranging from 0.01 to 0.43 for *Apis* (0.26 ± 0.11; mean ± SD hereafter) and from 0.01 to 0.42 for *Bombus* models (0.25 ± 0.11); and *CCB* values ranging from 0.01 to 0.25 in the *Apis* model (0.14 ± 0.06) and from 0.01 to 0.34 in the *Bombus* model (0.18 ± 0.08). Flower depth values ranged from 0.00 mm to 89.37 mm (12.49 ± 16.71 mm; see **Table S4**).

### Conspicuousness Across Pollination Systems

We found a significant effect of pollination system (*p* < 0.01 in all models, see **Table S6**) for both visual models in the achromatic (*ACB_Apis_*: *F* = 12.25; *ACB_Bombus_*: *F* = 12.44), chromatic channels (*CCB_Apis_*: *F* = 23.83; *CCB_Bombus_*: *F* = 21.62) and spectral purity (*SP_Apis_*: *F* = 19.95; *SP_Bombus_*: *F* = 17.75).

**Figure 3:**
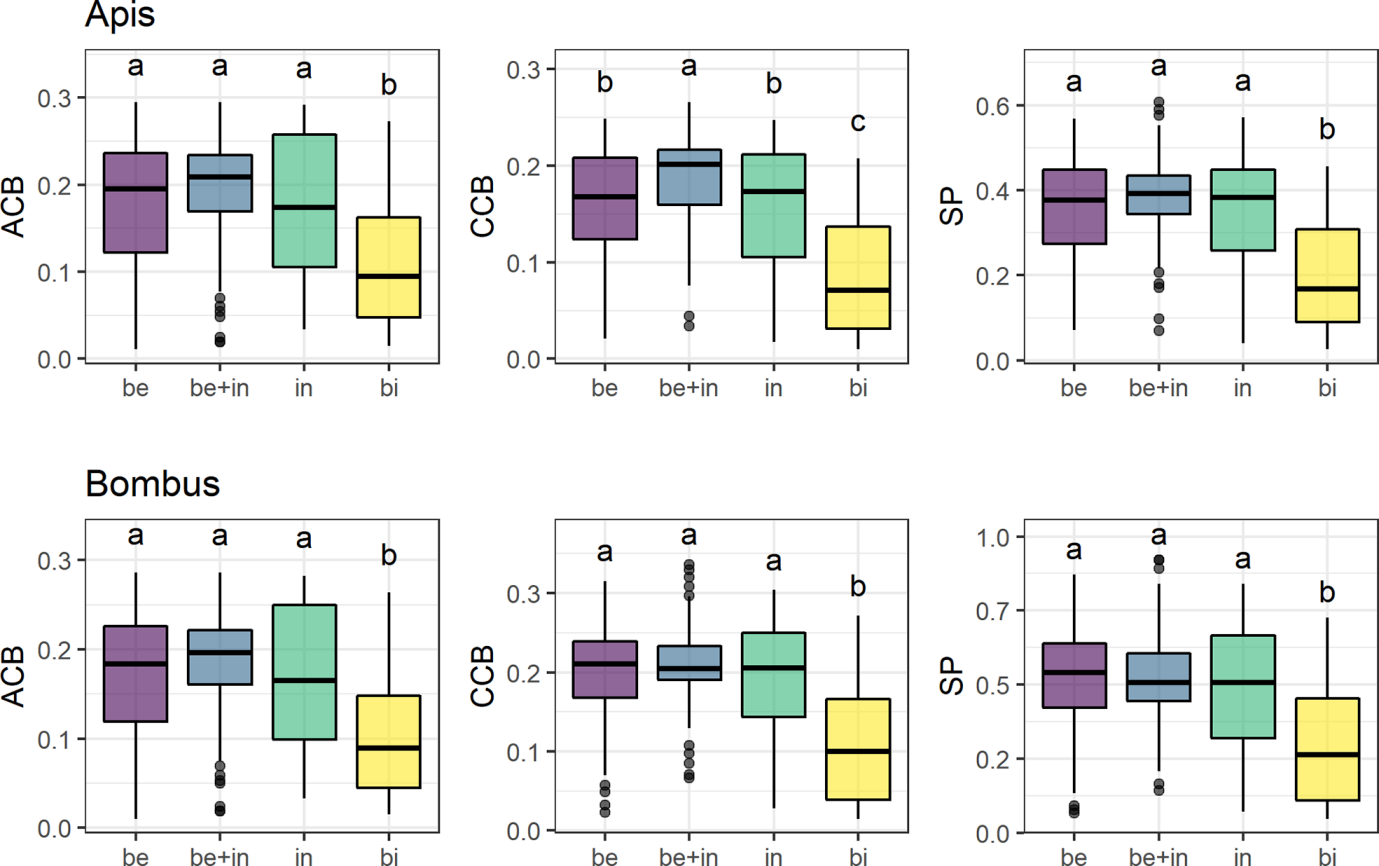
Mean achromatic (*ACB*) and chromatic (*CCB*) contrast against the background and spectral purity (*SP*) comparisons between flowers of 285 species belonging to four distinct pollination systems in the visual models for *Apis mellifera* and *Bombus terrestris*. Groups are: “Be” (species visited solely by bees, *N* = 137), “be+in” (species visited by both bees and other insects, *N* = 90), “in” (non-bee insect flowers, i.e., species visited by potentially red-sensitive insects, but not by bees; *N* = 26) and “bi” (species visited solely by birds, *N* = 32). Box-plots show the distribution of contrast and purity values for each pollination system: the thick horizontal line indicates the median; lower and upper hinges correspond to the first and third quartiles; whiskers extend from the hinge to the smallest/largest value at most 1.5 * *IQR* of the hinge and individual dots indicate outliers. Different letters represent significant differences between means after ANOVA and *post-hoc* Tukey’s HSD tests.

Overall, only the bird-flower group differed from the others in mean contrast against the background (**Figure 3**, see **Table S7**). Insect-flower groups (bee, non-bee insect, and bee+insect flowers) did not differ between them in most models, except in *CCB _Apis_* between bee and bee+insect flowers (**Figure 3**, third panel). We found significantly lower *ACB*, *CCB*, and *SP* for bird flowers in relation to all groups of insect flowers in both visual models (*p* < 0.01 for all models).

### Conspicuousness Comparisons Across Flower Color Categories

**Figure 4:**
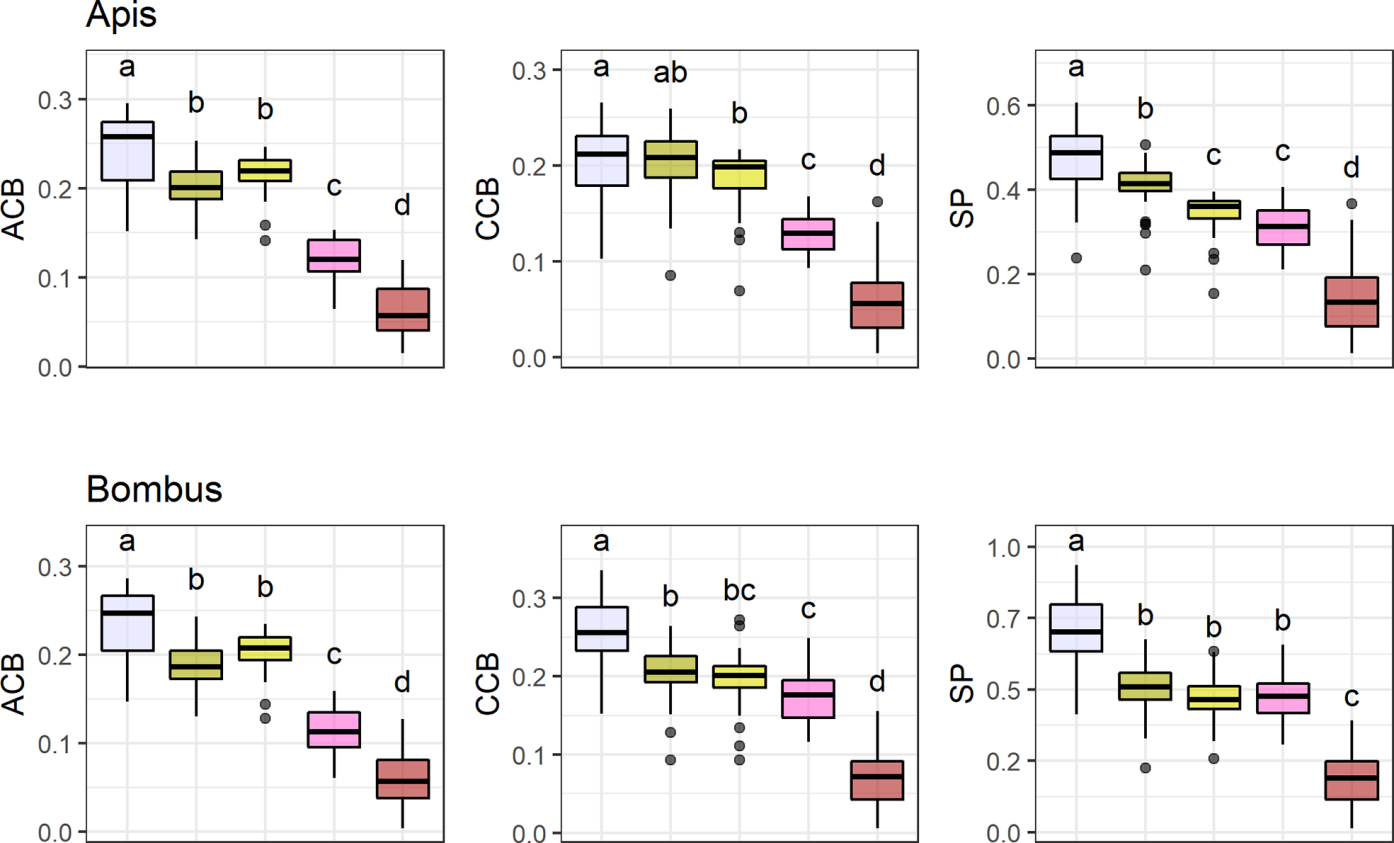
Mean achromatic (*ACB*) and chromatic (*CCB*) contrast against the background and spectral purity (*SP*) comparisons between the most frequent flower color categories. Color categories are UV-absorbing white (*N* = 9 7 species), UV-absorbing yellow (*N* = 63), UV-reflecting yellow (*N* = 37), UV-absorbing pink (*N* = 31), and UV-absorbing red (*N* = 55). Only colors with *N* > 30 were considered (totaling 288 species). Flower contrasts and spectral purity were computed for *Apis mellifera* and *Bombus terrestris*. Box-plots show the distribution of contrast and purity values for each pollination system: the thick horizontal line indicates the median; lower and upper hinges correspond to the first and third quartiles; whiskers extend from the hinge to the smallest/largest value at most 1.5 * *IQR* of the hinge and individual dots indicate outliers. Different letters represent significant differences between means after ANOVA and *post-hoc* Tukey’s HSD tests.

UV-absorbing red flowers presented the lowest contrast and purity values, while UV-absorbing white flowers were the most conspicuous ones in the eyes of bees (**Figure 4**; see **Table S8**). UV-reflecting and UV-absorbing yellow flowers did not differ in bee contrast in any of the models (but differed in one model for spectral purity), and presented lower contrasts and purity than UV-absorbing white flowers in most models, but higher ones than UV-absorbing red flowers as did UV-absorbing pink flowers. Not surprisingly, UV-absorbing red flowers were the most frequent in the bird pollination system, whereas most bee and non-bee insect flowers were UV-absorbing white (**Figure 4**).

### Association Between Conspicuousness and Flower Depth

**Figure 5:**
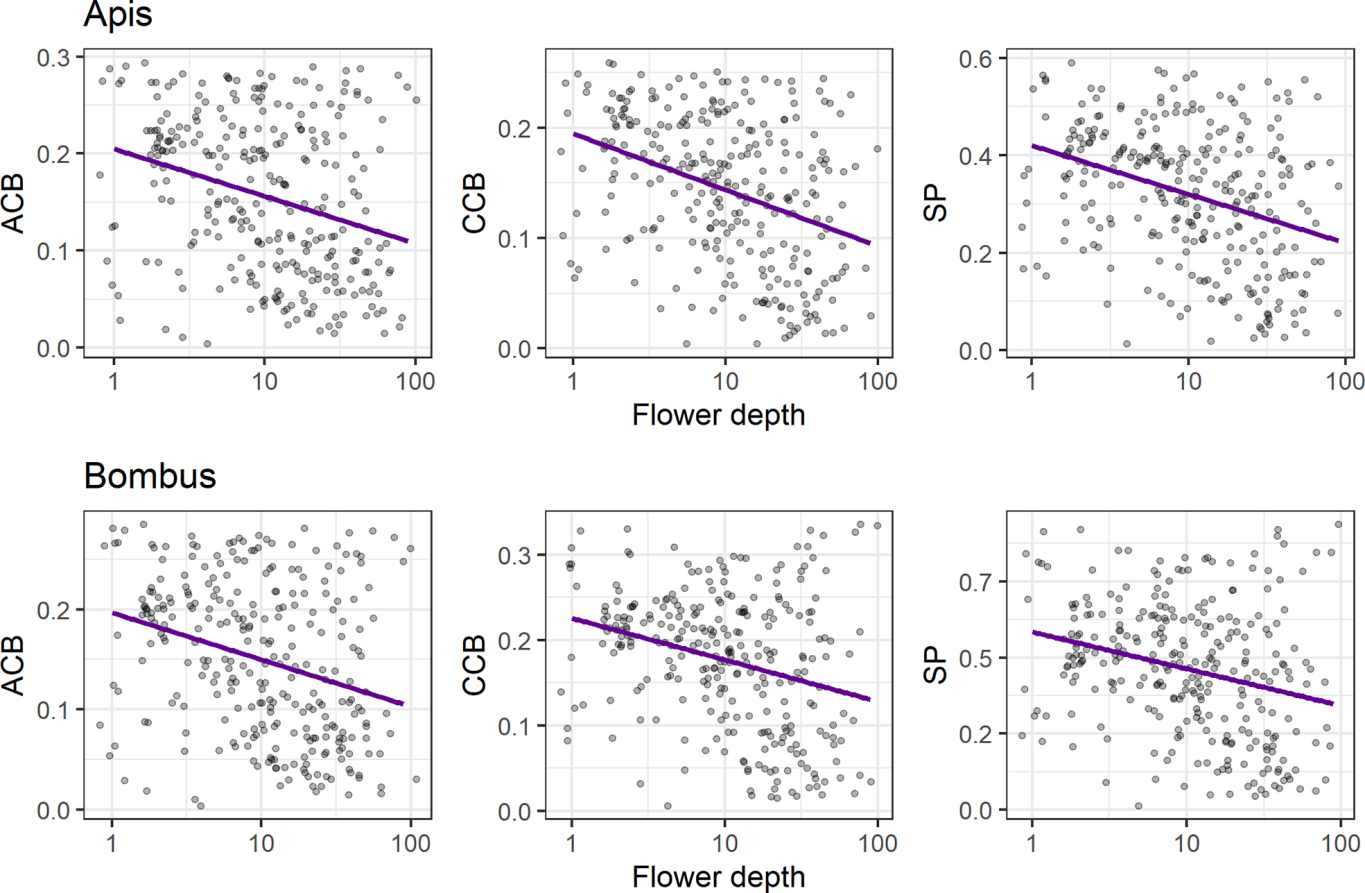
Linear regression analyses of bee contrasts against the background (achromatic, *ACB*; chromatic, *CCB*) and spectral purity (*SP*) with flower depth (mm), for *Apis mellifera* (upper panels) and *Bombus terrestris (lower panels*). Left panels: negative associations between flower depth and *ACB* (*R* ^2^ = 0.09 and *p* < 0.01 for both *Apis* and *Bombus* models); central panels: negative associations between flower depth and *CCB* for the *Apis* model (upper panel; *R* ^2^ = 0.15 and *p* < 0.01) and for the *Bombus* model (lower panel; *R* ^2^ = 0.08 and *p* < 0.01); right panels: negative associations between flower depth and *SP* for the *Apis* model (upper panel; *R* ^2^ = 0.12 and *p* < 0.01) and for the *Bombus* model (lower panel; *R* ^2^ = 0.07 and *p* < 0.01). Only flowers with flower depth ≥ 1 mm were considered (*N* = 286 species), regardless of pollination system. We applied log scale to flower depth for normalization and horizontal jittering in order to prevent overplotting.

We found a negative relationship between conspicuousness to bees and flower depth (*p* < 0.01) for all models (**Figure 5**, see **Table S9**), indicating that flower contrasts against the background decrease with flower depth in both achromatic (*ACB_Apis_* and *ACB_Bombus_*: *R* ^2^ = 0.09) and chromatic channels (*CCB_Apis_*: *R* ^2^ = 0.15; *CCB_Bombus_*: *R* ^2^ = 0.09), as does spectral purity (*SP_Apis_*: *R* ^2^ = 0.12; *SP_Bombus_*: *R* ^2^ = 0.07).

## Discussion

Overall, the three pollination systems composed by insect visitors here studied seem to be hardly distinguishable in detectability properties to bee vision, for all three conspicuousness metrics used (i.e., *ACB*, *CCB*, and *SP*). This suggests that insect flowers use similar strategies in their intensity of visual signals detectable by bees. Two processes could account for these results. First, in spite of notable red-sensitive exceptions, an overall conservative visual system in flower-visiting insects with similar points of optimal discriminability in the spectrum [i.e., overall similar visual systems; Chittka (1996)], selecting similar patterns of reflectance. Second, bee visits could have positive effects (or at least not incur in costs) in insect flowers in general (Sampson et al., 2004). In any case, our results did not support the bee-avoidance hypothesis (Raven, 1972) through color signals for non-bee insect-pollinated species. Thus, if some kind of bee avoidance does occur in insect flowers, it may happen either only for specific systems with red flowers and based on specific guilds that were underrepresented in our sample, or in other sensory channels rather than the visual one. Unlike birds, insects use a great number of olfactory cues when foraging for floral resources, not relying solely on spectral signals (Andersson et al., 2015). Hence, plants that specialize in different insect pollinator groups dispose of a large spectrum of olfactory and even tactile, heat and electric signals, besides visual ones, which may be combined to create exclusive channels with their most effective pollinators [Wedzony and Filek (1998); Schiestl and Dötterl (2012); Telles et al. (2017)]. In this way, visual bee avoidance does not seem to be such a widespread phenomenon in insect-pollinated flowers as it is for bird-pollinated ones.

Nevertheless, bird flowers differed from bee flowers in our database with significantly lower mean contrasts and spectral purity, which gives additional support to the bee-avoidance hypothesis [Bergamo et al. (2019); Camargo et al. (2019)]. This is probably due to two factors. First, the high frequency of UV-absorbing red flowers, the least conspicuous ones, in the bird group, which was to be expected since most bird-specialized flowers are red [Grant (1966); Wilson et al. (2006)]. Moreover, the predominance of UV-absorbing white flowers in the bee group, which were the most conspicuous ones in our models. These results reinforce the role of reddish flowers and bracts in bee-avoiding bird-specialized species as a general pattern rather than a phenomenon restricted to specific communities.

We found a negative relationship between conspicuousness to bees and flower depth, similarly to the results of *CCB* in hummingbird flowers by Bergamo et al. (2019) and of *ACB* in insect flowers by Binkenstein et al. (2017). Our results indicate that the low conspicuousness of long-tubed flowers to bees might not be a phenomenon exclusive to bird-pollinated systems. Moreover, long-tubed flowers in our study presented low conspicuousness in both achromatic and chromatic channels, expanding previous results found for the chromatic channel in hummingbird flowers of the Brazilian Atlantic forest (Bergamo et al., 2019). Thereby, our results support that flower depth and detectability to bees are two of a set of key traits mediating bee avoidance in flowers (Gegear et al., 2017). These two traits might be under similar selective pressures, since long flower tubes are associated with increased nectar robbing by bees [Navarro and Medel (2009); Rojas-Nossa et al. (2016)], and bee nectar robbers have a strong negative effect on the reproductive success of hummingbird-pollinated flowers [Irwin et al. (2001); Bergamo and Sazima (2018)]. On the other hand, for long-tubed bee-pollinated flowers, lower contrasts and purity might signal nectar inaccessibility to short-tongued bees (and even to other insects with short mouthparts), decreasing foraging energy losses for the potential visitors and enhancing reproductive success for the plant through reduced interference of these with legitimate pollinators (Binkenstein et al., 2017). Moreover, total flower depth might be regarded as a proxy for area of attraction, since it is one way of determining overall flower size (Wolf et al., 1976). In theory, a species under selection for increased floral area of attraction may have smaller investment in pigments, which might cause a decrease in its contrast (Blarer et al., 2002). Such a trade-off might be at play in tubular flowers, yielding a negative association between flower size and bee contrast regardless of pollination system.

## Conclusion

Our results corroborate the bee-avoidance hypothesis for bird flowers. However, for insect flowers, all groups of flowers presented a similar intensity of color signals to bees, giving no support to visual bee avoidance. Therefore, our results suggest that sensory exclusion of bees *via* color signals is a mechanism exclusive to bird-pollinated species. We also found a negative association between flower depth and conspicuousness in the visual models, regardless of pollination system, suggesting a general correlation between two bee-avoiding traits that may act synergistically. Further studies could enlighten the role of long corolla tubes in insect-bird pollinator shifts, investigating whether long-tubed species are more likely to shift to bird pollination. The possibility of visual bee-avoidance mechanisms should also be investigated for pollination systems based on specific non-bee insect guilds, especially those with long tubes, in communities where bees are also present. Overall, our results reinforce the importance of spectral signaling in bird-pollinated systems and its interplay with flower depth.

## Data Availability Statement

All datasets presented in this study are included in the article/**Supplementary Material**.

## Author Contributions

LF and MR-G devised the initial research project. GC and CA collected the data supervised by LF. GC conducted data analysis with support from PB and interpreted its findings along with LF. GC wrote the manuscript with assistance from LF, PB, and MR-G.

## Funding

The National Council for Scientific and Technological Development (CNPq) provided support for the project through funding and in the form of undergraduate scholarships (PIBIC) to GC and CA and research scholarships to PB (PDJ), LF (PQ) and MR-G (PVE). The Rio de Janeiro Carlos Chagas Filho Research Foundation (FAPERJ) provided a grant to LF (CNE). The Coordination for the Improvement of Higher Education Personnel (CAPES) provided partial funding (Finance Code 001).

## Conflict of Interest

The authors declare that the research was conducted in the absence of any commercial or financial relationships that could be construed as a potential conflict of interest.
