# Supplementary material for: Flower Conspicuousness to Bees Across Pollination Systems: A Generalized Test of the Bee-Avoidance Hypothesis
Source: Front Plant Sci. 2020 Sep 24;11:558684. doi: 10.3389/fpls.2020.558684 (PMC7542005; doi:10.3389/fpls.2020.558684)
Supplement: Supplementary file 1 [file DataSheet_1.pdf]

Supplementary Material

1 SUPPLEMENTARY DATA

REFERENCES

Abdullahi, G., Sule, H., Chimoya, I., Isah, M., et al. (2011). Diversity and relative distribution of honeybees foraging plants in some selected reserves in Mubi Region, Sudan Savannah ecological zone of Nigeria. *Advances in Applied Science Research* 2, 388–395

Arnold, S., Savolainen, V., and Chittka, L. (2008). FReD: the floral reflectance spectra database. *Nature Precedings* , 1–1

Azevedo, I. F. P. d. et al. (2014). Sistemas reprodutivos em espécies não-arbóreas de fragmento de Floresta Atlântica do sudeste brasileiro: diversidade, frequência e condições derivadas

Azmi, G. R., Wahizatul A and Mohamed, N. Z. (2012). The importance of carpenter Bee, Xylocopa varipuncta (Hymenoptera: Apidae) as pollination agent for mangrove community of Setiu Wetland, Terengganu. *Sains Malaysiana* 41, 1057–1062

Bhalchandra, W., Baviskar, R., and Nikam, T. (2014). Diversity of nectariferous and polleniferous bee flora at Anjaneri and Dugarwadi hills of Western Ghats of Nasik district (MS) India. *Journal of Entomology and Zoology Studies* 2, 244–249

Blomberg, S. P., Garland Jr, T., and Ives, A. R. (2003). Testing for phylogenetic signal in comparative data: behavioral traits are more labile. *Evolution* 57, 717–745

Bolstad, P. and Bawa, K. (1982). Self incompatibility in Gmelina arborea L.(Verbenaceae). *Silvae Genet* 31, 19–21

Borba, E. L. and Braga, P. I. S. (2003). Biologia reprodutiva de Pseudolaelia corcovadensis (Orchidaceae): melitofilia e autocompatibilidade em uma Laeliinae basal. *Brazilian Journal of Botany* 26, 541–549

Bruneau, A. (1997). Evolution and homology of bird pollination syndromes in Erythrina (Leguminosae). *American Journal of Botany* 84, 54–71

Canela, M. B. F. and Sazima, M. (2003). Aechmea pectinata: A Hummingbird-dependent Bromeliad with inconspicuous flowers from the rainforest in South-eastern Brazil. *Annals of Botany* 92, 731–737

Capucho, L. C., Dalcolmo, S., de Holanda Formigoni, T., and da Silva, A. G. (2007). Bromélias e beija-flores: um modelo observacional para testar hipóteses sobre correlações e adaptações morfológicas recíprocas. *Rodriguesia* , 49–58

Cate, C. A. (2007). *Monitoring, assessing and evaluating the pollinator species (Hymenoptera: apoidea) found on a native brush site, a revegetated site and an urban garden*. Ph.D. thesis, Texas A & M University

Cestari, C. (2009). Epiphyte plants use by birds in Brazil. *Oecologia Brasiliensis* , 689–712

Cingel, N. A. v. d. (2001). *An atlas of orchid pollination: European orchids* (CRC Press)

Coser, T. d. S. et al. (2008). Bromeliaceae Juss. dos campos rupestres do Parque Estadual do Itacolomi, Minas Gerais, Brasil: florística e aspectos fenológicos

Costa, V., Pimentel, R., Chagas, M., Alves, G., and Castro, C. (2017). Petal micromorphology and its relationship to pollination. *Plant Biology* 19, 115–122

Etcheverry, A. V. and Alemán, C. E. T. (2005). Reproductive Biology of Erythrina falcata (Fabaceae: Papilionoideae). *Biotropica: The Journal of Biology and Conservation* 37, 54–63

Feinsinger, P. (1983). Variable nectar secretion in a Heliconia species pollinated by hermit hummingbirds. *Biotropica* , 48–52

Feinsinger, P., Wolfe, J. A., and Swarm, L. A. (1982). Island ecology: reduced hummingbird diversity and the pollination biology of plants, Trinidad and Tobago, West Indies. *Ecology* 63, 494–506

Fohouo, F., Ngakou, A., and Kengni, B. S. (2009). Pollination and yield responses of cowpea (Vigna unguiculata L. Walp.) to the foraging activity of Apis mellifera adansonii (Hymenoptera: Apidae) at Ngaoundéré (Cameroon). *African Journal of Biotechnology* 8

Fonseca, L. C., Vizentin-Bugoni, J., Rech, A. R., and Alves, M. A. S. (2015). Plant-hummingbird interactions and temporal nectar availability in arestinga from Brazil. *Anais da Academia Brasileira de Ciências* 87, 2163–2175

Franceschinelli, E. V. (2005). The pollination biology of two species of Helicteres (Malvaceae) with different mechanisms of pollen deposition. *Flora-Morphology, Distribution, Functional Ecology of Plants* 200, 65–73

Franceschinelli, E. V. and Bawa, K. S. (2000). The effect of ecological factors on the mating system of a South American shrub species (Helicteres brevispira). *Heredity* 84, 116–123

Freeman, C. E., Worthington, R. D., and Jackson, M. S. (1991). Floral nectar sugar compositions of some South and Southeast Asian species. *Biotropica* , 568–574

Galetti, M. (1993). Diet of the scaly-headed parrot (Pionus maximiliani) in a semideciduous forest in southeastern Brazil. *Biotropica* , 419–425

Glinos, E. and Cocucci, A. (2011). Pollination biology of Canna indica (Cannaceae) with particular reference to the functional morphology of the style. *Plant systematics and evolution* 291, 49–58

Gomiz, N. E., Torretta, J. P., and Aliscioni, S. S. (2013). Comparative anatomy of elaiophores and oil secretion in the genus Gomesa (Orchidaceae). *Turkish Journal of Botany* 37, 859–871

Ige, O., Olotuah, O., and Akerele, V. (2011). Floral biology and pollination ecology of cowpea (Vigna unguiculata L. Walp). *Modern Applied Science* 5, 74

Jaarsveld, E. v. (2012). Gasteria pulchra (Aiton) Haw.(Asphodelaceae)

Kato, M., Shibata, A., Yasui, T., and Nagamasu, H. (1999). Impact of introduced honeybees, Apis mellifera, upon native bee communities in the Bonin (Ogasawara) Islands. *Population Ecology* 41, 217–228

Kiill, L. H. P. and Drumond, M. A. (2001). Biologia floral e sistema reprodutivo de Gliricidia sepium (Jacq.) Steud.(Fabaceae-Papilionoidae) na região de Petrolina, Pernambuco. *Ciência Rural* 31, 597–601

Kimmel, T. M., do Nascimento, L. M., Piechowski, D., Sampaio, E. V., Rodal, M. J. N., and Gottsberger, G. (2010). Pollination and seed dispersal modes of woody species of 12-year-old secondary forest in the Atlantic Forest region of Pernambuco, NE Brazil. *Flora-Morphology, Distribution, Functional Ecology of Plants* 205, 540–547

Layek, U., Bhakat, R., and Karmakar, P. (2015). Foraging behavior of Apis florea Fabricius during winter and spring-summer in Bankura and Paschim Medinipur districts, West Bengal. *Global Journal of Bio Sciences and Biotechnology* 4, 255–263

Lopes, A. V. et al. (2002). Polinização por beija-flores em remanescente da Mata Atlântica Pernambucana, Nordeste do Brasil

Magalhães, A. F. P., Maruyama, P. K., Tavares, L. A. F., and Martins, R. L. (2018). The relative importance of hummingbirds as pollinators in two bromeliads with contrasting floral specializations and breeding systems. *Botanical Journal of the Linnean Society* 188, 316–326

Martinelli, G. (1995). *Reproductive biology of Bromeliaceae in the Atlantic rainforest of southeastern Brazil*. Ph.D. thesis, University of St Andrews

McMullen, C. K. (1989). The Galápagos carpenter bee, just how important is it? *Noticias de Galápagos* 48, 16–18

Molgo, I. E., De Dijn, B. P., et al. (2007). Orchids and orchid bees of the Brownsberg, Nassau and Lely ranges. In *RAP Bulletin of Biological Assessment: A Rapid Biological Assessment of the Lely and Nassau Plateaus, Suriname (with additional information on the Brownsberg Plateau)* (BioOne)

Morton, E. S. (1979). Effective pollination of Erythrina fusca by the Orchard Oriole (Icterus spurius): Coevolved behavioral manipulation? *Annals of the Missouri Botanical Garden* , 482–489

Murali, S., Dhananjaya, P., Reddy, G., Tavaragondi, V., et al. (2013). Study of insect pollinator's diversity in Asystasia gangetica (L.). *Environment and Ecology* 31, 804–806

Nicolson, S. W. (2002). Pollination by passerine birds: why are the nectars so dilute? *Comparative Biochemistry and Physiology Part B: Biochemistry and Molecular Biology* 131, 645–652

Pemberton, R. and Liu, H. (2011). Naturalized yellow cowhorn orchid, Cyrtopodium flavum (Orchidaceae), spreading in Florida. *Journal of the Botanical Research Institute of Texas* , 331–335

Pinheiro, M., Brito, V. L. G. d., and Sazima, M. (2018). Pollination biology of melittophilous legume tree species in the Atlantic Forest in Southeast Brazil. *Acta Botanica Brasilica* 32, 410–425

Qian, H. and Jin, Y. (2016). An updated megaphylogeny of plants, a tool for generating plant phylogenies and an analysis of phylogenetic community structure. *Journal of Plant Ecology* 9, 233–239

Raju, A. S. and Reddi, C. S. (2000). Foraging behaviour of carpenter bees (genus Xylocopa: Xylocopidae: Hymenoptera) and the pollination of Indian plants. *JOURNAL-BOMBAY NATURAL HISTORY SOCIETY* 97, 381–389

Rasoloarijao, T. M., Ramavovololona, P., Ramamonjisoa, R., Clemencet, J., Lebreton, G., and Delatte, H. (2019). Pollen morphology of melliferous plants for Apis mellifera unicolor in the tropical rainforest of Ranomafana National Park, Madagascar. *Palynology* 43, 292–320

Rose, M.-J. and Barthlott, W. (1994). Coloured Pollen in Cactaceae: a Mimetic Adaptation to Hummingbird-Pollination? *Botanica Acta* 107, 402–406

Sazima, I., Buzato, S., and Sazima, M. (1995). The Saw-billed Hermit Ramphodon naevius and its flowers in southeastern Brazil. *Journal f"ur ornithologie* 136, 195–206

Sazima, M. and Sazima, I. (1999). The perching bird Coereba flaveola as a co-pollinator of bromeliad flowers in southeastern Brazil. *Canadian Journal of Zoology* 77, 47–51

Schleuning, M., Templin, M., Huamán, V., Vadillo, G. P., Becker, T., Durka, W., et al. (2011). Effects of inbreeding, outbreeding, and supplemental pollen on the reproduction of a hummingbird-pollinated clonal Amazonian herb. *Biotropica* 43, 183–191

Silva, A. L. G. d. and Pinheiro, M. C. B. (2009). Reproductive success of four species of Eugenia L.(Myrtaceae). *Acta Botanica Brasilica* 23, 526–534

Singh, A., Jaiswal, D. K., Singh, H., and Thakur, R. (2016). Diversity of bees' flora and floral calendar of native honeybees in Nagaland, India. *Advances in Life Sciences* 5, 2285–2292

Siqueira Filho, J. A. d. and Machado, I. C. S. (2001). Biologia reprodutiva de Canistrum aurantiacum E. Morren (Bromeliaceae) em remanescente da Floresta Atlântica, nordeste do Brasil. *Acta Botanica Brasilica* 15, 427–443

Soper, J. and Beggs, J. (2013). Assessing the impact of an introduced bee, Anthidium manicatum, on pollinator communities in New Zealand. *New Zealand journal of botany* 51, 213–228

Sugiura, N. (2013). Specialized pollination by carpenter bees in Calanthe striata (Orchidaceae), with a review of carpenter bee pollination in orchids. *Botanical Journal of the Linnean Society* 171, 730–743

Tripp, E. A. and McDade, L. A. (2013). Time-calibrated phylogenies of hummingbirds and hummingbird-pollinated plants reject a hypothesis of diffuse co-evolution. *Aliso: A Journal of Systematic and Evolutionary Botany* 31, 89–103

Varalakshmi, P. and Raju, A. (2013). Psychophilous and melittophilous pollination syndrome in Tridax procumbens L.(Asteraceae). *TAPROBANICA: The Journal of Asian Biodiversity* 5

Viana, B. F. and Alves-dos Santos, I. (2002). Bee diversity of the coastal sand dunes of Brazil. *Pollinating bees: the conservation link between agriculture and nature* , 135–153

Wilms, W., Imperatriz-Fonseca, V. L., and Engels, W. (1996). Resource partitioning between highly eusocial bees and possible impact of the introduced Africanized honey bee on native stingless bees in the Brazilian Atlantic rainforest. *Studies on Neotropical Fauna and Environment* 31, 137–151

Wolowski, M., Saad, C. F., Ashman, T.-L., and Freitas, L. (2013). Predominance of self-compatibility in hummingbird-pollinated plants in the Neotropics. *Naturwissenschaften* 100, 69–79

Zanne, A. E., Tank, D. C., Cornwell, W. K., Eastman, J. M., Smith, S. A., FitzJohn, R. G., et al. (2014). Three keys to the radiation of angiosperms into freezing environments. *Nature* 506, 89–92

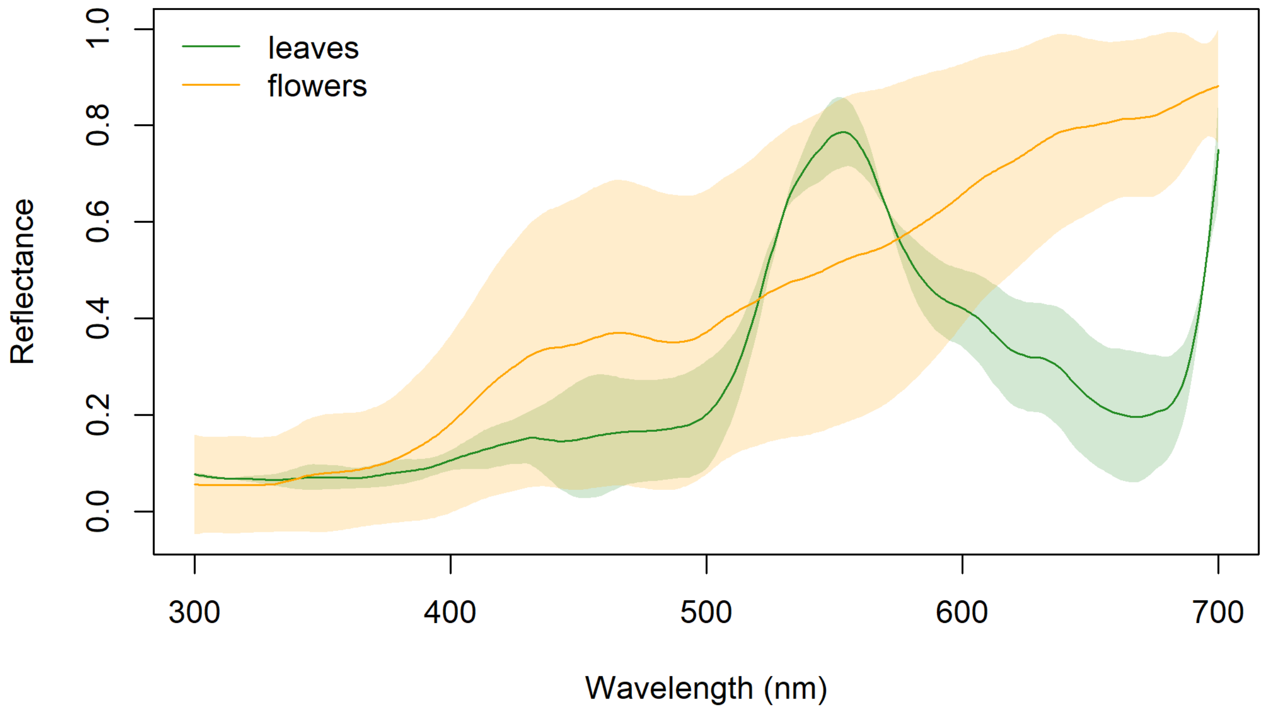

Figure S1. Mean flower and leaf reflectance curves of the whole dataset used for analyses. Shaded areas indicate standard deviation, which for flowers was calculated for all species ($N = 389$) and for leaves between mean reflectance of leaves collected at JBRJ and the mean reflectance of leaves extracted from FReD. The mean leaf reflectance curve was used as the standard background for computation of bee contrasts for all species.

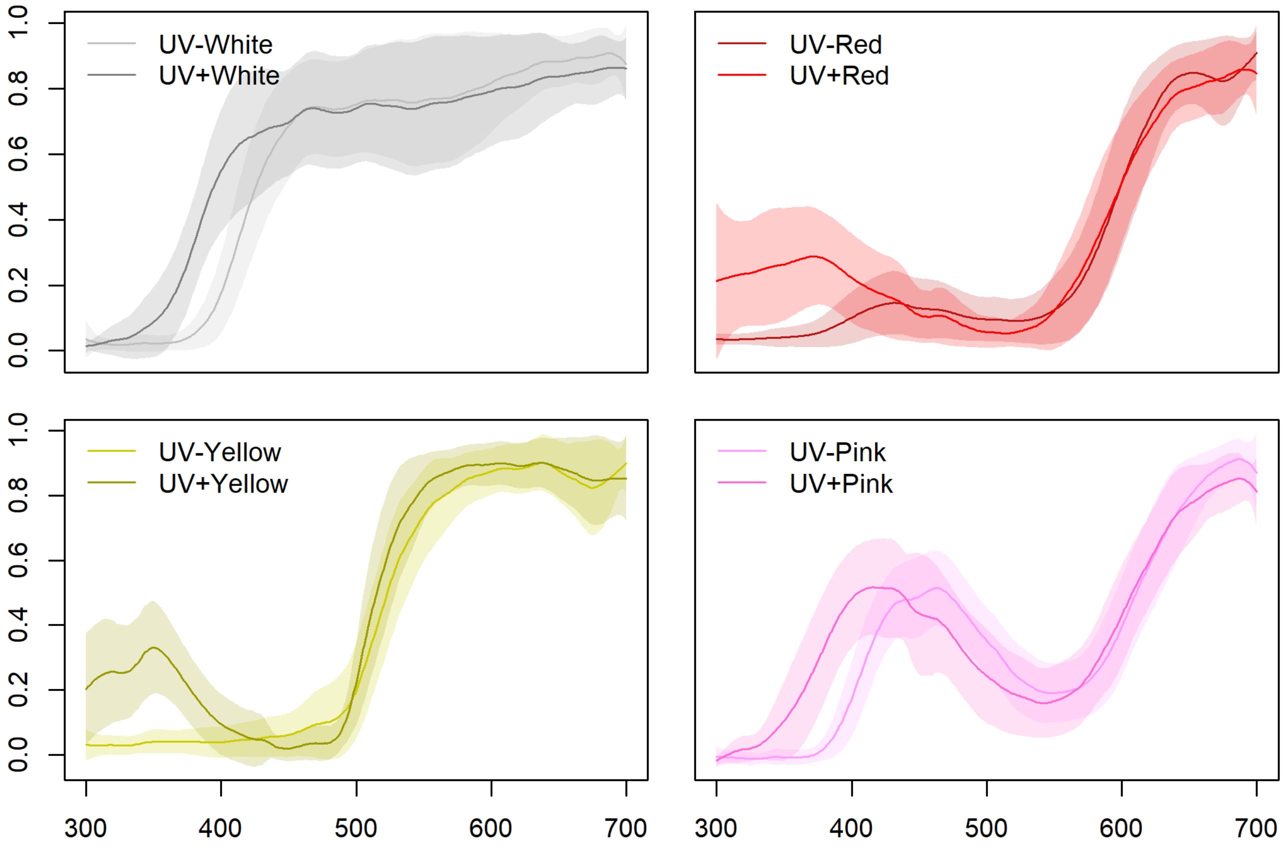

Figure S2. Mean flower reflectance curves for the most frequent color categories in the dataset: UV-White ("$u-b+g+r+$", $N = 97$ plant species), UV+White ("$u+b+g+r+$", $N = 21$), UV-Red ("$u-b-g-r+$", $N = 55$), UV+Red ("$u+b-g-r+$", $N = 11$), UV+Yellow ("$u+b-g+r+$", $N = 37$), UV-Yellow ("$u-b-g+r+$", $N = 63$) UV-Pink ("$u-b+g-r+$", $N = 31$) and UV+Pink ("$u+b+g-r+$", $N = 13$).

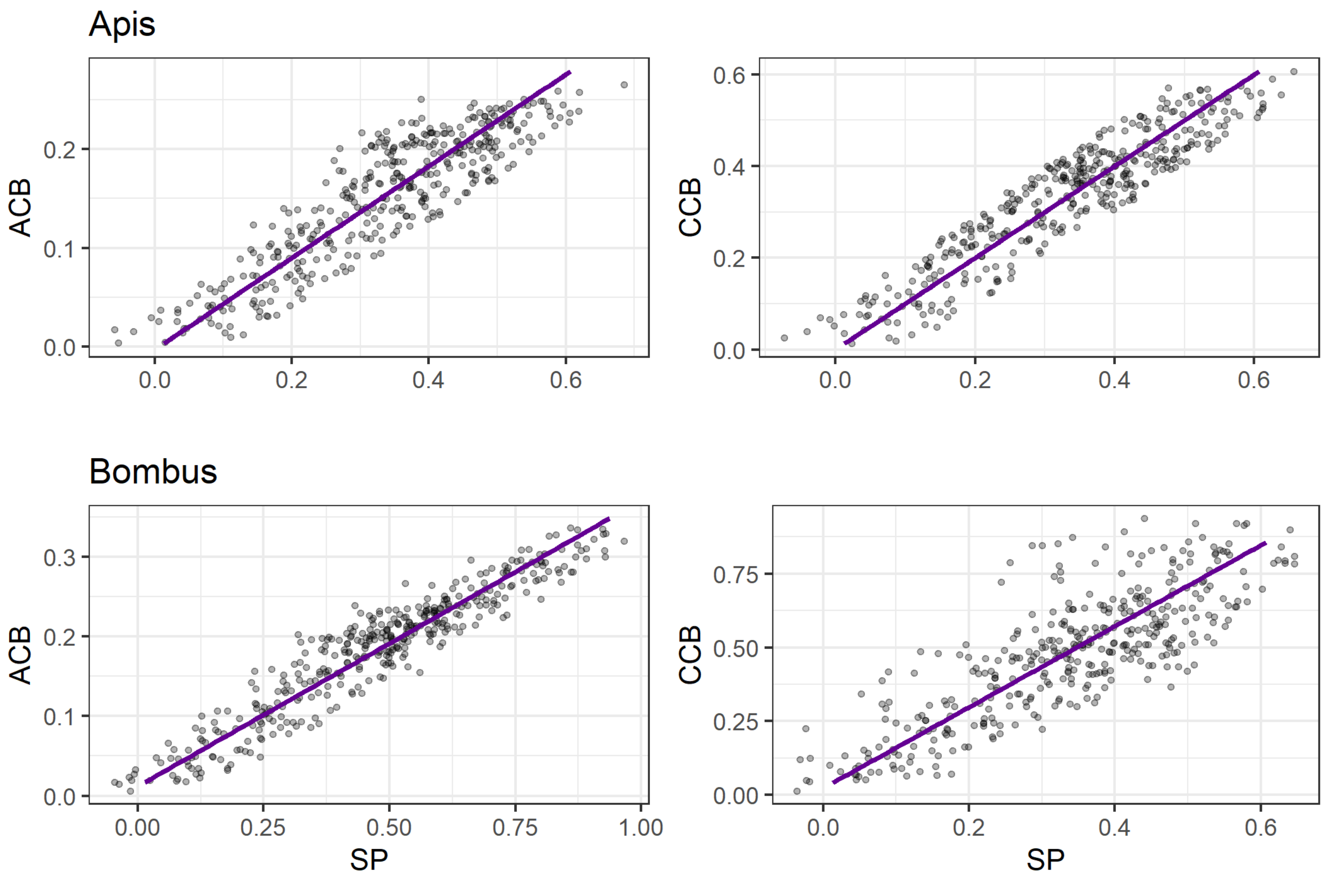

Figure S3. Linear regression analyses between bee contrasts against the background (achromatic: ACB, chromatic: CCB) and spectral purity (SP), for the visual models of *Apis mellifera* (upper panels) and *Bombus terrestris* (lower panels).

Table S1: Complete list of species used for analyses and their respective taxonomic groups.

Order	Family	Genus	Species
Apiales	Apiaceae	Ainsworthia	*Ainsworthia trachycarpa* Boiss.
Apiales	Apiaceae	Scandix	*Scandix pecten-veneris* L.
Asparagales	Amaryllidaceae	Allium	*Allium neapolitanum* Cirillo
Asparagales	Amaryllidaceae	Allium	*Allium nigrum* L.
Asparagales	Amaryllidaceae	Allium	*Allium trifoliatum* Cirillo
Asparagales	Amaryllidaceae	Crinum	*Crinum asiaticum* L.
Asparagales	Amaryllidaceae	Crinum	*Crinum latifolium* L.
Asparagales	Amaryllidaceae	Habranthus	*Habranthus robustus* Herb. ex Sweet
Asparagales	Amaryllidaceae	Leopoldia	*Leopoldia comosa* Parl.
Asparagales	Amaryllidaceae	Leopoldia	*Leopoldia longipes* (Boiss.) Losinsk.
Asparagales	Asparagaceae	Bellevalia	*Bellevalia flexuosa* Boiss.
Asparagales	Asparagaceae	Dracaena	*Dracaena reflexa* Lam.
Asparagales	Asparagaceae	Sansevieria	*Sansevieria cylindrica* Bojer ex Hook.
Asparagales	Asparagaceae	Scilla	*Scilla hyacinthoides* L.
Asparagales	Asparagaceae	Yucca	*Yucca aloifolia* L.
Asparagales	Hypoxidaceae	Hypoxis	*Hypoxis decumbens* L.
Asparagales	Iridaceae	Dietes	*Dietes bicolor* (Steud.) Sweet ex Klatt
Asparagales	Iridaceae	Neomarica	*Neomarica candida* (Hassl.) Sprague
Asparagales	Orchidaceae	Cyrtopodium	*Cyrtopodium flavum* (Nees) Link & Otto ex Rchb.
Asparagales	Orchidaceae	Encyclia	*Encyclia cordigera* (Kunth) Dressler
Asparagales	Orchidaceae	Gomesa	*Gomesa flexuosa* (Lodd.) M.W.Chase & N.H.Williams
Asparagales	Orchidaceae	Heterotaxis	*Heterotaxis sessilis* (Sw.) F.Barros
Asparagales	Orchidaceae	Limodorum	*Limodorum abortivum* (L.) Sw.
Asparagales	Orchidaceae	Orchis	*Orchis italica* Poir.
Asparagales	Orchidaceae	Phalaenopsis	*Phalaenopsis amabilis* (L.) Blume
Asparagales	Orchidaceae	Platanthera	*Platanthera clavellata* (Michx.) Luer
Asparagales	Orchidaceae	Pseudolaelia	*Pseudolaelia corcovadensis* Porto & Brade
Asparagales	Orchidaceae	Renanthera	*Renanthera coccinea* Lour.
Asparagales	Orchidaceae	Sobralia	*Sobralia yauaperyensis* Barb.Rodr.
Asparagales	Orchidaceae	Spathoglottis	*Spathoglottis unguiculata* (Labill.) Rchb.f.
Asparagales	Xanthorrhoeaceae	Aloe	*Aloe arborescens* Mill.
Asparagales	Xanthorrhoeaceae	Aloe	*Aloe ciliaris* Haw.
Asparagales	Xanthorrhoeaceae	Aloe	*Aloe glauca* Mill.

Asparagales	Xanthorrhoeaceae	Aloe	*Aloe* sp.
Asparagales	Xanthorrhoeaceae	Asphodelus	*Asphodelus aestivus* Brot.
Asparagales	Xanthorrhoeaceae	Gasteria	*Gasteria pulchra* (Aiton) Haw.
Asparagales	Xanthorrhoeaceae	Haworthia	*Haworthia attenuata* (Haw.) Haw.
Asterales	Asteraceae	Aaronsohnia	*Aaronsohnia factorovskyi* Warb. & Eig
Asterales	Asteraceae	Achillea	*Achillea santolina* Sibth. & Sm.
Asterales	Asteraceae	Anthemis	*Anthemis maris-mortui* Eig
Asterales	Asteraceae	Anthemis	*Anthemis melampodina* Delile
Asterales	Asteraceae	Anthemis	*Anthemis pseudocotula* Boiss.
Asterales	Asteraceae	Asteriscus	*Asteriscus graveolens* Less.
Asterales	Asteraceae	Calea	*Calea phyllolepis* Baker
Asterales	Asteraceae	Calendula	*Calendula arvensis* L.
Asterales	Asteraceae	Centaurea	*Centaurea aegyptiaca* L.
Asterales	Asteraceae	Centaurea	*Centaurea ammocyanus* Boiss.
Asterales	Asteraceae	Centaurea	*Centaurea pallescens* Delile
Asterales	Asteraceae	Cirsium	*Cirsium oleraceum* Scop.
Asterales	Asteraceae	Crepis	*Crepis aspera* L.
Asterales	Asteraceae	Crepis	*Crepis hierosolymitana* Boiss.
Asterales	Asteraceae	Crepis	*Crepis palaestina* Bornm.
Asterales	Asteraceae	Crepis	*Crepis sancta* (L.) Babc.
Asterales	Asteraceae	Crupina	*Crupina crupinastrum* Vis.
Asterales	Asteraceae	Emilia	*Emilia sonchifolia* (L.) DC. ex Wight
Asterales	Asteraceae	Erigeron	*Erigeron canadensis* L.
Asterales	Asteraceae	Gazania	*Gazania heterochaeta* DC.
Asterales	Asteraceae	Hedypnois	*Hedypnois rhagadioloides* (L.) F.W.Schmidt
Asterales	Asteraceae	Hieracium	*Hieracium laevigatum* Willd.
Asterales	Asteraceae	Hieracium	*Hieracium sabaudum* L.
Asterales	Asteraceae	Jacobaea	*Jacobaea vulgaris* Gaertn.
Asterales	Asteraceae	Lapsana	*Lapsana communis* L.
Asterales	Asteraceae	Launaea	*Launaea angustifolia* (Desf.) O.Kuntze
Asterales	Asteraceae	Launaea	*Launaea mucronata* Muschl.
Asterales	Asteraceae	Launaea	*Launaea nudicaulis* Hook.f.
Asterales	Asteraceae	Leontodon	*Leontodon laciniatus* (Bertol.) Widder
Asterales	Asteraceae	Leontodon	*Leontodon tuberosus* L.
Asterales	Asteraceae	Liatris	*Liatris spicata* Willd.
Asterales	Asteraceae	Matricaria	*Matricaria aurea* Sch.Bip.

Asterales	Asteraceae	Picris	*Picris longirostris* Sch.Bip.
Asterales	Asteraceae	Hieracium	*Pilosella officinarum* Vaill.
Asterales	Asteraceae	Pulicaria	*Pulicaria incisa* DC.
Asterales	Asteraceae	Rhagadiolus	*Rhagadiolus stellatus* Gaertn.
Asterales	Asteraceae	Scorzonera	*Scorzonera papposa* DC.
Asterales	Asteraceae	Scorzoneroides	*Scorzoneroides autumnalis* (L.) Moench
Asterales	Asteraceae	Senecio	*Senecio glaucus* L.
Asterales	Asteraceae	Senecio	*Senecio vernalis* Waldst. & Kit.
Asterales	Asteraceae	Solidago	*Solidago canadensis* L.
Asterales	Asteraceae	Sonchus	*Sonchus oleraceus* L.
Asterales	Asteraceae	Stifftia	*Stifftia chrysantha* J.C.Mikan
Asterales	Asteraceae	Taraxacum	*Taraxacum officinale* F.H.Wigg.
Asterales	Asteraceae	Tridax	*Tridax procumbens* (L.) L.
Asterales	Asteraceae	Tripleurospermum	*Tripleurospermum auriculatum* (Boiss.) Rech.fil.
Asterales	Asteraceae	Tussilago	*Tussilago farfara* L.
Asterales	Asteraceae	Urospermum	*Urospermum picroides* (L.) F.W.Schmidt
Asterales	Asteraceae	Ursinia	*Ursinia cakilefolia* DC.
Asterales	Asteraceae	Youngia	*Youngia japonica* (L.) DC.
Asterales	Campanulaceae	Campanula	*Campanula latifolia* L.
Asterales	Campanulaceae	Campanula	*Campanula rapunculoides* L.
Asterales	Campanulaceae	Campanula	*Campanula trachelium* L.
Asterales	Campanulaceae	Lobelia	*Lobelia anceps* L.f.
Asterales	Campanulaceae	Phyteuma	*Phyteuma betonicifolium* Vill.
Asterales	Campanulaceae	Phyteuma	*Phyteuma hedraianthifolium* R.Schulz
Asterales	Campanulaceae	Phyteuma	*Phyteuma hemisphaericum* L.
Asterales	Campanulaceae	Phyteuma	*Phyteuma nigrum* F.W.Schmidt
Boraginales	Boraginaceae	Alkanna	*Alkanna strigosa* Boiss. & Hohen.
Boraginales	Boraginaceae	Anchusa	*Anchusa officinalis* L.
Boraginales	Boraginaceae	Anchusa	*Anchusa strigosa* Banks & Sol.
Boraginales	Boraginaceae	Buglossoides	*Buglossoides incrassata* subsp. *incrassata*
Boraginales	Boraginaceae	Cynoglossum	*Cynoglossum officinale* L.
Boraginales	Boraginaceae	Echium	*Echium angustifolium* Mill.
Boraginales	Boraginaceae	Echium	*Echium rauwolfii* Delile
Boraginales	Boraginaceae	Myosotis	*Myosotis alpestris* F.W.Schmidt
Boraginales	Boraginaceae	Myosotis	*Myosotis decumbens* Host
Boraginales	Boraginaceae	Myosotis	*Myosotis stricta* Link ex Roem. & Schult.

Boraginales	Boraginaceae	Myosotis	*Myosotis vestergrenii* Stroh
Boraginales	Boraginaceae	Pulmonaria	*Pulmonaria mollis* Hornem.
Boraginales	Boraginaceae	Pulmonaria	*Pulmonaria obscura* Dumort.
Boraginales	Boraginaceae	Symphytum	*Symphytum brachycalyx* Boiss.
Boraginales	Brassicaceae	Cardaminopsis	*Arabidopsis arenosa* subsp. *arenosa* (L.) Lawalrée
Boraginales	Brassicaceae	Arabidopsis	*Arabidopsis thaliana* (L.) Heynh.
Boraginales	Brassicaceae	Capsella	*Capsella bursa-pastoris* (L.) Medik.
Boraginales	Brassicaceae	Cardamine	*Cardamine pratensis* L.
Boraginales	Brassicaceae	Diplotaxis	*Diplotaxis harra* Boiss.
Boraginales	Brassicaceae	Erucaria	*Erucaria pinnata* (Viv.) Tackholm & Boulos
Boraginales	Brassicaceae	Erysimum	*Erysimum cheiranthoides* L.
Boraginales	Brassicaceae	Hesperis	*Hesperis pendula* DC.
Boraginales	Brassicaceae	Isatis	*Isatis lusitanica* L.
Boraginales	Brassicaceae	Cardaria	*Lepidium draba* L.
Boraginales	Brassicaceae	Moricandia	*Moricandia nitens* E.Durand & Barratte
Boraginales	Brassicaceae	Sinapis	*Sinapis arvensis* O.F.Muell.
Boraginales	Brassicaceae	Sinapis	*Sinapis incana* L.
Brassicales	Brassicaceae	Zilla	*Zilla spinosa* Forssk.
Caryophyllales	Aizoaceae	Mesembryanthemum	*Mesembryanthemum cryptanthum* Hook.f.
Caryophyllales	Aizoaceae	Mesembryanthemum	*Mesembryanthemum nodiflorum* L.
Caryophyllales	Cactaceae	Cleistocactus	*Cleistocactus straussii* (Heese) Backeb.
Caryophyllales	Cactaceae	Mammillaria	*Mammillaria bombycina* Quehl
Caryophyllales	Cactaceae	Mammillaria	*Mammillaria prolifera* (Mill.) Haw.
Caryophyllales	Cactaceae	Nopalea	*Nopalea cochenillifera* (L.) Salm-Dyck
Caryophyllales	Cactaceae	Tacinga	*Tacinga palmadora* (Britton & Rose) N.P.Taylor & Stuppy
Caryophyllales	Caryophyllaceae	Arenaria	*Arenaria serpyllifolia* L.
Caryophyllales	Caryophyllaceae	Cerastium	*Cerastium holosteoides* Fries
Caryophyllales	Caryophyllaceae	Dianthus	*Dianthus carthusianorum* L.
Caryophyllales	Caryophyllaceae	Gymnocarpos	*Gymnocarpos decandrus* Forssk.
Caryophyllales	Caryophyllaceae	Gypsophila	*Gypsophila arabica* Barkoudah
Caryophyllales	Caryophyllaceae	Holosteum	*Holosteum umbellatum* L.
Caryophyllales	Caryophyllaceae	Moehringia	*Moehringia trinervia* (L.) Clairv.
Caryophyllales	Caryophyllaceae	Silene	*Silene acaulis* (L.) Jacq.
Caryophyllales	Caryophyllaceae	Silene	*Silene aegyptiaca* L.f.
Caryophyllales	Caryophyllaceae	Silene	*Silene flos-cuculi* (L.) Greuter & Burdet
Caryophyllales	Caryophyllaceae	Silene	*Silene nutans* L.

Caryophyllales	Caryophyllaceae	Stellaria	*Stellaria holostea* L.
Caryophyllales	Caryophyllaceae	Stellaria	*Stellaria palustris* Retz.
Caryophyllales	Nyctaginaceae	Bougainvillea	*Bougainvillea spectabilis* Willd.
Caryophyllales	Phytolaccaceae	Rivina	*Rivina humilis* L.
Caryophyllales	Polygonaceae	Bistorta	*Bistorta officinalis* subsp. *officinalis*
Caryophyllales	Tamaricaceae	Tamarix	*Tamarix nilotica* (Ehrenb.) Bunge
Celastrales	Celastraceae	Parnassia	*Parnassia palustris* L.
Dipsacales	Adoxaceae	Viburnum	*Viburnum opulus* L.
Dipsacales	Caprifoliaceae	Knautia	*Knautia arvensis* Coult.
Dipsacales	Caprifoliaceae	Knautia	*Knautia dipsacifolia* (Host) Kreutzer
Dipsacales	Caprifoliaceae	Symphoricarpos	*Symphoricarpos albus* (L.) C.Koch
Ericales	Balsaminaceae	Impatiens	*Impatiens walleriana* Hook.f.
Ericales	Ericaceae	Arbutus	*Arbutus andrachne* L.
Ericales	Ericaceae	Arctostaphylos	*Arctostaphylos uva-ursi* (L.) Spreng.
Ericales	Ericaceae	Calluna	*Calluna vulgaris* (L.) Hull
Ericales	Ericaceae	Phyllodoce	*Phyllodoce caerulea* (L.) Bab.
Ericales	Ericaceae	Vaccinium	*Vaccinium vitis-idaea* L.
Ericales	Lecythidaceae	Gustavia	*Gustavia augusta* L.
Ericales	Primulaceae	Cyclamen	*Cyclamen persicum* Mill.
Ericales	Primulaceae	Primula	*Primula elatior* (L.) L.
Ericales	Theaceae	Camellia	*Camellia japonica* L.
Ericales	Theaceae	Camellia	*Camellia sinensis* (L.) Kuntze
Fabales	Fabaceae	Amherstia	*Amherstia nobilis* Wall.
Fabales	Fabaceae	Anagyris	*Anagyris foetida* L.
Fabales	Fabaceae	Andira	*Andira legalis* (Vell.) Toledo
Fabales	Fabaceae	Astragalus	*Astragalus amalecitanus* Boiss.
Fabales	Fabaceae	Astragalus	*Astragalus glycyphyllos* L.
Fabales	Fabaceae	Astragalus	*Astragalus sanctus* Boiss.
Fabales	Fabaceae	Brownea	*Brownea ariza* Benth.
Fabales	Fabaceae	Calicotome	*Calicotome villosa* (Poir.) Link
Fabales	Fabaceae	Calliandra	*Calliandra harrisii* (Lindl.) Benth.
Fabales	Fabaceae	Camoensia	*Camoensia scandens* (Welw.) J.B.Gillett
Fabales	Fabaceae	Cercis	*Cercis siliquastrum* L.
Fabales	Fabaceae	Colutea	*Colutea istria* Mill.
Fabales	Fabaceae	Dahlstedtia	*Dahlstedtia pinnata* (Benth.) Malme
Fabales	Fabaceae	Erythrina	*Erythrina falcata* Benth.

Fabales	Fabaceae	Erythrina	*Erythrina fusca* Lour.
Fabales	Fabaceae	Erythrina	*Erythrina speciosa* Andrews
Fabales	Fabaceae	Glircidia	*Gliricidia sepium* (Jacq.) Walp.
Fabales	Fabaceae	Lathyrus	*Lathyrus blepharicarpus* Boiss.
Fabales	Fabaceae	Lathyrus	*Lathyrus gorgoni* Parl.
Fabales	Fabaceae	Lathyrus	*Lathyrus pratensis* L.
Fabales	Fabaceae	Lathyrus	*Lathyrus vernus* Bernh.
Fabales	Fabaceae	Lebeckia	*Lebeckia halenbergensis* Merxm. & A.Schreiber
Fabales	Fabaceae	Lotus	*Lotus longesiliquosus* R.Roem.
Fabales	Fabaceae	Mucuna	*Mucuna bennettii* F.Muell.
Fabales	Fabaceae	Onobrychis	*Onobrychis crista-galli* Lam.
Fabales	Fabaceae	Ononis	*Ononis natrix* L.
Fabales	Fabaceae	Oxytropis	*Oxytropis jacquinii* Bunge
Fabales	Fabaceae	Oxytropis	*Oxytropis neglecta* J.Gay ex Ten.
Fabales	Fabaceae	Retama	*Retama raetam* Webb & Berthel.
Fabales	Fabaceae	Saraca	*Saraca thaipingensis* Prain
Fabales	Fabaceae	Coronilla	*Securigera varia* (L.) Lassen
Fabales	Fabaceae	Swartzia	*Swartzia simplex* var. *grandiflora* (Raddi) Cowan
Fabales	Fabaceae	Trifolium	*Trifolium clypeatum* L.
Fabales	Fabaceae	Trifolium	*Trifolium repens* L.
Fabales	Fabaceae	Trifolium	*Trifolium resupinatum* L.
Fabales	Fabaceae	Trifolium	*Trifolium stellatum* L.
Fabales	Fabaceae	Trigonella	*Trigonella caelesyriaca* Boiss.
Fabales	Fabaceae	Trigonella	*Trigonella kotschyi* Benth.
Fabales	Fabaceae	Vicia	*Vicia hybrida* L.
Fabales	Fabaceae	Vicia	*Vicia sativa* L.
Fabales	Fabaceae	Vigna	*Vigna unguiculata* (L.) Walp.
Fagales	Betulaceae	Corylus	*Corylus avellana* L.
Gentianales	Apocynaceae	Allamanda	*Allamanda cathartica* L.
Gentianales	Apocynaceae	Cascabela	*Cascabela thevetia* (L.) Lippold
Gentianales	Apocynaceae	Kopsia	*Kopsia fruticosa* (Roxb.) A.DC.
Gentianales	Apocynaceae	Malouetia	*Malouetia arborea* (Vell.) Miers
Gentianales	Apocynaceae	Vincetoxicum	*Vincetoxicum hirundinaria* subsp. *hirundinaria* Medik.
Gentianales	Rubiaceae	Galium	*Galium verum* L.
Gentianales	Rubiaceae	Ixora	*Ixora coccinea* L.
Gentianales	Rubiaceae	Mussaenda	*Mussaenda philippica* A.Rich.

Gentianales	Rubiaceae	Randia	*Randia* sp.
Geraniales	Geraniaceae	Geranium	*Geranium sylvaticum* L.
Lamiales	Acanthaceae	Acanthus	*Acanthus montanus* (Nees) T.Anderson
Lamiales	Acanthaceae	Alectis	*Alectis ciliaris* (Bloch, 1787)
Lamiales	Acanthaceae	Asystasia	*Asystasia gangetica* (L.) T.Anderson
Lamiales	Acanthaceae	Barleria	*Barleria cristata* L.
Lamiales	Acanthaceae	Barleria	*Barleria repens* Nees
Lamiales	Acanthaceae	Justicia	*Justicia brandegeeana* Wassh. & L.B.Sm.
Lamiales	Acanthaceae	Justicia	*Justicia gendarussa* Burm.f.
Lamiales	Acanthaceae	Megaskepasma	*Megaskepasma erythrochlamys* Lindau
Lamiales	Acanthaceae	Odontonema	*Odontonema tubaeforme* (Bertol.) Kuntze
Lamiales	Acanthaceae	Pachystachys	*Pachystachys lutea* Nees
Lamiales	Acanthaceae	Pachystachys	*Pachystachys spicata* (Ruiz & Pav.) Wassh.
Lamiales	Acanthaceae	Sanchezia	*Sanchezia oblonga* Ruiz & Pav.
Lamiales	Acanthaceae	Sanchezia	*Sanchezia speciosa* Leonard
Lamiales	Acanthaceae	Thunbergia	*Thunbergia erecta* (Benth.) T.Anderson
Lamiales	Acanthaceae	Thunbergia	*Thunbergia mysorensis* (Wight) T.Anderson
Lamiales	Bignoniaceae	Handroanthus	*Handroanthus heptaphyllus* (Vell.) Mattos
Lamiales	Bignoniaceae	Tanaecium	*Tanaecium pyramidatum* (Rich.) L.G.Lohmann
Lamiales	Gesneriaceae	Episcia	*Episcia cupreata* (Hook.) Hanst.
Lamiales	Lamiaceae	Ajuga	*Ajuga chamaepitys* subsp. *chia* (Schreb.) Arcang.
Lamiales	Lamiaceae	Ajuga	*Ajuga genevensis* L.
Lamiales	Lamiaceae	Ajuga	*Ajuga pyramidalis* L.
Lamiales	Lamiaceae	Ajuga	*Ajuga reptans* L.
Lamiales	Lamiaceae	Clerodendrum	*Clerodendrum quadriloculare* (Blanco) Merr.
Lamiales	Lamiaceae	Clerodendrum	*Clerodendrum thomsoniae* Balf.f.
Lamiales	Lamiaceae	Clinopodium	*Clinopodium vulgare* L.
Lamiales	Lamiaceae	Congea	*Congea tomentosa* Roxb.
Lamiales	Lamiaceae	Galeopsis	*Galeopsis bifida* Boenn.
Lamiales	Lamiaceae	Galeopsis	*Galeopsis pubescens* Bess.
Lamiales	Lamiaceae	Galeopsis	*Galeopsis tetrahit* L.
Lamiales	Lamiaceae	Glechoma	*Glechoma hederacea* L.
Lamiales	Lamiaceae	Gmelina	*Gmelina arborea* Roxb. ex Sm.
Lamiales	Lamiaceae	Gmelina	*Gmelina asiatica* L.
Lamiales	Lamiaceae	Lamium	*Lamium album* L.
Lamiales	Lamiaceae	Lamium	*Lamium galeobdolon* (L.) L.

Lamiales	Lamiaceae	Lamium	*Lamium garganicum* L.
Lamiales	Lamiaceae	Mentha	*Mentha aquatica* L.
Lamiales	Lamiaceae	Ocimum	*Ocimum basilicum* L.
Lamiales	Lamiaceae	Origanum	*Origanum vulgare* L.
Lamiales	Lamiaceae	Phlomoides	*Phlomoides laciniata* (L.) Kamelin & Makhm.
Lamiales	Lamiaceae	Premna	*Premna cordifolia* Roxb.
Lamiales	Lamiaceae	Salvia	*Salvia dominica* L.
Lamiales	Lamiaceae	Salvia	*Salvia fruticosa* Mill.
Lamiales	Lamiaceae	Salvia	*Salvia hierosolymitana* Boiss.
Lamiales	Lamiaceae	Salvia	*Salvia lanigera* Poir.
Lamiales	Lamiaceae	Salvia	*Salvia splendens* Sellow ex Schult.
Lamiales	Lamiaceae	Satureja	*Satureja thymbra* L.
Lamiales	Lamiaceae	Stachys	*Stachys recta* L.
Lamiales	Lamiaceae	Stachys	*Stachys sylvatica* L.
Lamiales	Lamiaceae	Thymus	*Thymus serpyllum* L.
Lamiales	Lentibulariaceae	Pinguicula	*Pinguicula alpina* L.
Lamiales	Orobanchaceae	Bartsia	*Bartsia alpina* L.
Lamiales	Orobanchaceae	Melampyrum	*Melampyrum pratense* L.
Lamiales	Orobanchaceae	Melampyrum	*Melampyrum sylvaticum* L.
Lamiales	Orobanchaceae	Pedicularis	*Pedicularis comosa* L.
Lamiales	Orobanchaceae	Pedicularis	*Pedicularis recutita* L.
Lamiales	Plantaginaceae	Linaria	*Linaria vulgaris* Mill.
Lamiales	Plantaginaceae	Nanorrhinum	*Nanorrhinum scoparium* (Brouss. ex Spreng.) Yousefi & Zarre
Lamiales	Plantaginaceae	Russelia	*Russelia equisetiformis* Schltdl. & Cham.
Lamiales	Plantaginaceae	Veronica	*Veronica arvensis* L.
Lamiales	Plantaginaceae	Veronica	*Veronica chamaedrys* L.
Lamiales	Plantaginaceae	Veronica	*Veronica prostrata* L.
Lamiales	Plantaginaceae	Veronica	*Veronica spicata* L.
Lamiales	Scrophulariaceae	Scrophularia	*Scrophularia xanthoglossa* Boiss.
Lamiales	Scrophulariaceae	Verbascum	*Verbascum densiflorum* Bertol.
Lamiales	Scrophulariaceae	Verbascum	*Verbascum lychnitis* L.
Lamiales	Verbenaceae	Duranta	*Duranta erecta* L.
Lamiales	Verbenaceae	Lantana	*Lantana camara* L.
Lamiales	Verbenaceae	Stachytarpheta	*Stachytarpheta Vahl* Pohl ex Schauer
Liliales	Smilacaceae	Smilax	*Smilax* sp.
Malpighiales	Euphorbiaceae	Euphorbia	*Euphorbia hierosolymitana* Boiss.

Malpighiales	Linaceae	Linum	*Linum pubescens* Banks & Solander
Malvales	Cistaceae	Cistus	*Cistus incanus* L.
Malvales	Cistaceae	Cistus	*Cistus salviifolius* L.
Malvales	Cistaceae	Helianthemum	*Helianthemum ventosum* Boiss.
Malvales	Cistaceae	Helianthemum	*Helianthemum vesicarium* Boiss.
Malvales	Malvaceae	Alcea	*Alcea acaulis* Alef.
Malvales	Malvaceae	Alcea	*Alcea dissecta* (Baker f.) Zohary
Malvales	Malvaceae	Guazuma	*Guazuma ulmifolia* Lam.
Malvales	Malvaceae	Helicteres	*Helicteres brevispira* A.Juss.
Malvales	Malvaceae	Hibiscus	*Hibiscus rosa-sinensis* L.
Malvales	Malvaceae	Hibiscus	*Hibiscus schizopetalus* (Dyer) Hook.f
Malvales	Malvaceae	Hibiscus	*Hibiscus tiliaceus* L.
Malvales	Malvaceae	Malvaviscus	*Malvaviscus arboreus* Cav.
Malvales	Malvaceae	Pavonia	*Pavonia alnifolia* A.St.-Hil.
Myrtales	Combretaceae	Combretum	*Combretum indicum* (L.) DeFilipps
Myrtales	Combretaceae	Combretum	*Combretum paniculatum* Vent.
Myrtales	Combretaceae	Combretum	*Combretum rotundifolium* Rich.
Myrtales	Myrtaceae	Eugenia	*Eugenia brasiliensis* Lam.
Myrtales	Myrtaceae	Eugenia	*Eugenia uniflora* L.
Myrtales	Myrtaceae	Syzygium	*Syzygium malaccense* (L.) Merr. & L.M.Perry
Myrtales	Onagraceae	Epilobium	*Epilobium angustifolium* subsp. *angustifolium* L.
Myrtales	Onagraceae	Epilobium	*Epilobium parviflorum* (Schreb.) Schreb.
Mytales	Lythraceae	Cuphea	*Cuphea racemosa* (L.f.) Spreng.
Oxalidales	Oxalidaceae	Oxalis	*Oxalis debilis* Kunth
Pandanales	Velloziaceae	Vellozia	*Vellozia candida* J.C.Mikan
Poales	Bromeliaceae	Aechmea	*Aechmea aquilega* (Salisb.) Griseb.
Poales	Bromeliaceae	Aechmea	*Aechmea bambusoides* L.B.Sm. & Reitz
Poales	Bromeliaceae	Aechmea	*Aechmea blanchetiana* (Baker) L.B.Sm.
Poales	Bromeliaceae	Aechmea	*Aechmea bromeliifolia* Baker ex Benth. & Hook.f.
Poales	Bromeliaceae	Aechmea	*Aechmea eurycorymbus* Harms
Poales	Bromeliaceae	Aechmea	*Aechmea floribunda* Mart. ex Schult. & Schult.f.
Poales	Bromeliaceae	Aechmea	*Aechmea nudicaulis* (L.) Griseb
Poales	Bromeliaceae	Aechmea	*Aechmea pectinata* Baker
Poales	Bromeliaceae	Aechmea	*Aechmea purpureorosea* (Hook.) Wawra
Poales	Bromeliaceae	Aechmea	*Aechmea tomentosa* Mez
Poales	Bromeliaceae	Aechmea	*Aechmea vallerandii* (Carrière) Erhardt, Götz & Seybold.

Poales	Bromeliaceae	Aechmea	*Aechmea* sp.
Poales	Bromeliaceae	Alcantarea	*Alcantarea extensa* (L.B.Sm.) J.R.Grant
Poales	Bromeliaceae	Ananas	*Ananas bracteatus* (Lindl.) Schult. & Schult.f.
Poales	Bromeliaceae	Billbergia	*Billbergia amoena* Lindl.
Poales	Bromeliaceae	Billbergia	*Billbergia pyramidalis* Lindl.
Poales	Bromeliaceae	Canistrum	*Canistrum aurantiacum* E.Morren
Poales	Bromeliaceae	Canistrum	*Canistrum fragrans(Linden)* Mabb.
Poales	Bromeliaceae	Canistrum	*Canistrum superbum* (Lindm.) Mez
Poales	Bromeliaceae	Deuterocohnia	*Deuterocohnia meziana* Kuntze ex Mez
Poales	Bromeliaceae	Dyckia	*Dyckia brevifolia* hort. ex Baker
Poales	Bromeliaceae	Dyckia	*Dyckia encholirioides* (Gaudich.) Mez
Poales	Bromeliaceae	Hohenbergia	*Hohenbergia stellata* Schult. & Schult.f.
Poales	Bromeliaceae	Neoregelia	*Neoregelia cruenta* (Graham) L.B.Sm
Poales	Bromeliaceae	Pitcairnia	*Pitcairnia staminea* Lodd.
Poales	Bromeliaceae	Quesnelia	*Quesnelia arvensis* (Vell.) Mez
Poales	Bromeliaceae	Quesnelia	*Quesnelia liboniana* (De Jonghe) Mez
Poales	Bromeliaceae	Quesnelia	*Quesnelia* sp.
Poales	Bromeliaceae	Tillandsia	*Tillandsia polystachia* (L.) L.
Poales	Bromeliaceae	Tillandsia	*Tillandsia stricta* Sol. ex Ker Gawl.
Poales	Bromeliaceae	Tillandsia	*Tillandsia tenuifolia* L.
Poales	Bromeliaceae	Vriesea	*Vriesea neoglutinosa* Mez
Ranunculales	Papaveraceae	Chelidonium	*Chelidonium majus* L.
Ranunculales	Papaveraceae	Papaver	*Papaver hybridum* L.
Ranunculales	Papaveraceae	Papaver	*Papaver rhoeas* L.
Ranunculales	Papaveraceae	Papaver	*Papaver umbonatum* Boiss.
Ranunculales	Ranunculaceae	Aconitum	*Aconitum septentrionale* Koelle
Ranunculales	Ranunculaceae	Anemone	*Anemone coronaria* L.
Ranunculales	Ranunculaceae	Anemone	*Anemone nemorosa* L.
Ranunculales	Ranunculaceae	Anemone	*Anemone ranunculoides* L.
Ranunculales	Ranunculaceae	Ficaria	*Ficaria verna* subsp. *verna*
Ranunculales	Ranunculaceae	Hepatica	*Hepatica nobilis* Schreb.
Ranunculales	Ranunculaceae	Ranunculus	*Ranunculus acris* L.
Ranunculales	Ranunculaceae	Ranunculus	*Ranunculus asiaticus* L.
Ranunculales	Ranunculaceae	Ranunculus	*Ranunculus marginatus* d'Urv.
Ranunculales	Ranunculaceae	Ranunculus	*Ranunculus millefolius* Banks & Solander
Rosales	Rosaceae	Agrimonia	*Agrimonia eupatoria* L.

Rosales	Rosaceae	Crataegus	*Crataegus azarolus* L.
Rosales	Rosaceae	Filipendula	*Filipendula ulmaria* (L.) Maxim.
Rosales	Rosaceae	Fragaria	*Fragaria viridis* Duchesne
Rosales	Rosaceae	Geum	*Geum rivale* L.
Rosales	Rosaceae	Potentilla	*Potentilla heptaphylla* L.
Rosales	Rosaceae	Potentilla	*Potentilla reptans* L.
Rosales	Rosaceae	Prunus	*Prunus padus* L.
Sapindales	Meliaceae	Carapa	*Carapa guianensis* Aubl.
Sapindales	Meliaceae	Guarea	*Guarea guidonia* (L.) Sleumer
Sapindales	Rutaceae	Ruta	*Ruta chalepensis* L.
Saxifragales	Crassulaceae	Bryophyllum	*Bryophyllum fedtschenkoi* (Raym.-Hamet & H.Perrier) Lauz.-March.
Saxifragales	Crassulaceae	Bryophyllum	*Bryophyllum laetivirens* (Desc.) V.V.Byalt
Saxifragales	Crassulaceae	Echeveria	*Echeveria* sp.
Saxifragales	Crassulaceae	Sedum	*Hylotelephium maximum* (L.) J.Holub
Solanales	Convolvulaceae	Ipomoea	*Ipomoea purpurea* (L.) Roth
Solanales	Convolvulaceae	Ipomoea	*Ipomoea squamosa* Choisy
Solanales	Convolvulaceae	Jacquemontia	*Jacquemontia* sp.
Solanales	Convolvulaceae	Merremia	*Merremia dissecta* (Jacq.) Hallier f.
Solanales	Solanaceae	Brunfelsia	*Brunfelsia uniflora* (Pohl) D.Don
Solanales	Solanaceae	Hyoscyamus	*Hyoscyamus aureus* L.
Solanales	Solanaceae	Lycium	*Lycium shawii* Roem. & Schult.
Zingiberales	Cannaceae	Canna	*Canna indica* L.
Zingiberales	Heliconiaceae	Heliconia	*Heliconia metallica* Planch. & Linden ex Hook.
Zingiberales	Heliconiaceae	Heliconia	*Heliconia pendula* Wawra
Zingiberales	Heliconiaceae	Heliconia	*Heliconia psittacorum* L.f.
Zingiberales	Marantaceae	Maranta	*Maranta leuconeura* E.Morren
Zingiberales	Marantaceae	Stromanthe	*Stromanthe* sp.
Zingiberales	Marantaceae	Thalia	*Thalia geniculata* L.
Zingiberales	Musaceae	Musa	*Musa ornata* Roxb.
Zingiberales	Zingiberaceae	Alpinia	*Alpinia zerumbet* (Pers.) B.L.Burtt & R.M.Sm.
Zingiberales	Zingiberaceae	Etlingera	*Etlingera elatior* (Jack) R.M.Sm.
Zingiberales	Zingiberaceae	Hedychium	*Hedychium coccineum* Buch.-Ham. ex Sm.

Table S2: Flower color categories and mean reflectance intensity at each waveband considered (**UV**: from 201 to 300 nm; **Blue**: 301 – 400 nm; **Green**: 401 – 500 nm and **Red**: 501 – 600 nm) for each species of the dataset used for analyses. **Source** indicates whether data were extracted from the Floral Reflectance Database (FReD) or collected at the Botanical Garden of Rio de Janeiro (JBRJ) whereas **Structure** indicates which plant structure was considered for spectral analysis.

**Species**	**Source**	**Structure**	**Color**	**Category**	**UV**	**Blue**	**Green**	**Red**
Aaronsohnia factorovskyi	FReD	flower	UV-Yellow	u-b-g+r+	0.021	0.065	0.649	0.815
Acanthus montanus	JBRJ	flower	UV+White	u+b+g+r+	0.148	0.800	0.837	0.911
Achillea santolina	FReD	flower	UV-Yellow	u-b-g+r+	0.021	0.065	0.649	0.815
Aconitum septentrionale	FReD	flower	UV-Pink	u-b+g-r+	0.054	0.407	0.277	0.649
Aechmea aquilega	JBRJ	bract	UV-Red	u-b-g-r+	0.011	0.218	0.320	0.809
Aechmea bambusoides	JBRJ	bract	UV-Red	u-b-g-r+	0.004	0.139	0.136	0.702
Aechmea blanchetiana	JBRJ	bract	UV-Red	u-b-g-r+	0.049	0.087	0.214	0.658
Aechmea bromeliifolia	JBRJ	bract	UV-Red	u-b-g-r+	0.008	0.077	0.127	0.907
Aechmea eurycorymbus	JBRJ	flower	UV-Yellow	u-b-g+r+	0.038	0.073	0.440	0.782
Aechmea floribunda	JBRJ	flower	UV-White	u-b+g+r+	0.008	0.694	0.786	0.775
Aechmea nudicaulis	JBRJ	bract	UV-Red	u-b-g-r+	0.003	0.044	0.161	0.776
Aechmea pectinata	JBRJ	flower	UV+White	u+b+g+r+	0.112	0.468	0.700	0.617
Aechmea purpureorosea	JBRJ	flower	UV-Pink	u-b+g-r+	0.029	0.398	0.227	0.693
Aechmea sp.	JBRJ	flower	UV+Yellow	u+b-g+r+	0.101	0.096	0.614	0.719
Aechmea tomentosa	JBRJ	bract	UV-Red	u-b-g-r+	0.036	0.143	0.255	0.736
Aechmea vallerandii	JBRJ	bract	UV-Red	u-b-g-r+	0.011	0.065	0.092	0.903
Agrimonia eupatoria	FReD	calyx	UV+Yellow	u+b-g+r+	0.346	0.024	0.648	0.879
Ainsworthia trachycarpa	FReD	flower	UV-White	u-b+g+r+	0.020	0.634	0.931	0.904
Ajuga chamaepitys	FReD	flower	UV-Yellow	u-b-g+r+	0.029	0.060	0.734	0.941
Ajuga genevensis	FReD	flower	UV+Blue	u+b+g-r-	0.140	0.465	0.388	0.496
Ajuga pyramidalis	FReD	flower	UV-White	u-b+g+r+	0.082	0.545	0.587	0.771
Ajuga reptans	FReD	flower	UV+Blue	u+b+g-r-	0.100	0.665	0.212	0.526
Alcantarea extensa	JBRJ	calyx	UV+Black	u+b-g-r-	0.213	0.085	0.138	0.408
Alcea acaulis	FReD	calyx	UV-White	u-b+g+r+	0.025	0.499	0.631	0.831
Alcea dissecta	FReD	flower	UV+Pink	u+b+g-r+	0.104	0.363	0.161	0.741
Alectis ciliaris	FReD	flower	UV-Blue	u-b+g-r-	0.086	0.561	0.307	0.566
Alkanna strigosa	FReD	flower	UV+Blue	u+b+g-r-	0.149	0.428	0.204	0.578
Allamanda cathartica	JBRJ	flower	UV+Yellow	u+b-g+r+	0.198	0.026	0.821	0.981

Allium neapolitanum	FReD	flower	UV+White	u+b+g+r+	0.217	0.836	0.870	0.861
Allium nigrum	FReD	flower	UV-White	u-b+g+r+	0.032	0.690	0.882	0.835
Allium trifoliatum	FReD	flower	UV+White	u+b+g+r+	0.108	0.821	0.887	0.882
Aloe arborescens	JBRJ	flower	UV-White	u-b+g+r+	0.060	0.534	0.919	0.846
Aloe ciliaris	JBRJ	flower	UV-Yellow	u-b-g+r+	0.090	0.159	0.430	0.923
Aloe glauca	JBRJ	flower	UV-Red	u-b-g-r+	0.029	0.147	0.341	0.761
Aloe sp.	JBRJ	flower	UV-Yellow	u-b-g+r+	0.008	0.229	0.449	0.929
Alpinia zerumbet	JBRJ	calyx	UV+Cyan	u+b+g+r-	0.189	0.819	0.491	0.542
Amherstia nobilis	JBRJ	flower	UV-Red	u-b-g-r+	0.097	0.068	0.341	0.890
Anagyris foetida	FReD	flower	UV-Green	u-b-g+r-	0.079	0.112	0.657	0.567
Ananas bracteatus	JBRJ	bract	UV-Red	u-b-g-r+	0.035	0.059	0.092	0.751
Anchusa officinalis	FReD	flower	UV+Blue	u+b+g-r-	0.107	0.334	0.116	0.434
Anchusa strigosa	FReD	flower	UV+Blue	u+b+g-r-	0.103	0.338	0.063	0.410
Andira legalis	JBRJ	flower	UV+Red	u+b-g-r+	0.158	0.196	0.017	0.725
Anemone coronaria	FReD	flower	UV-Red	u-b-g-r+	0.022	0.062	0.075	0.644
Anemone nemorosa	FReD	flower	UV-White	u-b+g+r+	0.025	0.734	0.891	0.931
Anemone ranunculoides	FReD	flower	UV+Yellow	u+b-g+r+	0.177	0.017	0.748	0.985
Anthemis maris-mortui	FReD	calyx	UV-Yellow	u-b-g+r+	0.028	0.063	0.653	0.870
Anthemis melampodina	FReD	calyx	UV-Yellow	u-b-g+r+	0.018	0.015	0.623	0.896
Anthemis pseudocotula	FReD	calyx	UV-Yellow	u-b-g+r+	0.023	0.010	0.618	0.873
Arabidopsis arenosa	FReD	flower	UV-White	u-b+g+r+	0.009	0.816	0.982	0.982
Arabidopsis thaliana	FReD	flower	UV-White	u-b+g+r+	0.025	0.721	0.983	0.983
Arbutus andrachne	FReD	flower	UV-White	u-b+g+r+	0.016	0.430	0.844	0.897
Arctostaphylos uva-ursi	FReD	flower	UV-White	u-b+g+r+	0.070	0.568	0.649	0.844
Arenaria serpyllifolia	FReD	flower	UV-White	u-b+g+r+	0.062	0.630	0.851	0.939
Asphodelus aestivus	FReD	flower	UV+White	u+b+g+r+	0.253	0.830	0.857	0.872
Asteriscus graveolens	FReD	calyx	UV-Yellow	u-b-g+r+	0.027	0.032	0.598	0.913
Astragalus amalecitanus	FReD	flower	UV-White	u-b+g+r+	0.051	0.564	0.684	0.814
Astragalus glycyphyllos	FReD	flower	UV+White	u+b+g+r+	0.162	0.464	0.947	0.894
Astragalus sanctus	FReD	flower	UV-White	u-b+g+r+	0.051	0.564	0.684	0.814
Asystasia gangetica	JBRJ	flower	UV-White	u-b+g+r+	0.035	0.687	0.876	0.937
Barleria cristata	JBRJ	flower	UV-Pink	u-b+g-r+	0.048	0.664	0.398	0.769
Barleria repens	JBRJ	flower	UV+Red	u+b-g-r+	0.144	0.067	0.092	0.867
Bartsia alpina	FReD	flower	UV+Black	u+b-g-r-	0.115	0.252	0.170	0.429
Bellevalia flexuosa	FReD	flower	UV-White	u-b+g+r+	0.044	0.538	0.837	0.875
Billbergia amoena	JBRJ	flower	UV-Cyan	u-b+g+r-	0.020	0.303	0.693	0.551

Billbergia pyramidalis	JBRJ	bract	UV-Red	u-b-g-r+	0.007	0.101	0.198	0.849
Bistorta officinalis	FReD	flower	UV-White	u-b+g+r+	0.041	0.519	0.604	0.898
Bougainvillea spectabilis	JBRJ	bract	UV+Pink	u+b+g-r+	0.225	0.507	0.015	0.749
Brownea ariza	JBRJ	flower	UV-Red	u-b-g-r+	0.000	0.000	0.018	0.780
Brunfelsia uniflora	JBRJ	flower	UV-Pink	u-b+g-r+	0.041	0.456	0.204	0.630
Bryophyllum fedtschenkoi	JBRJ	flower	UV-Red	u-b-g-r+	0.004	0.013	0.144	0.668
Bryophyllum laetivirens	JBRJ	flower	UV+Red	u+b-g-r+	0.104	0.204	0.136	0.724
Buglossoides incrassata	FReD	flower	UV-White	u-b+g+r+	0.027	0.560	0.833	0.866
Calea phyllolepis	JBRJ	flower	UV-Yellow	u-b-g+r+	0.006	0.010	0.674	0.950
Calendula arvensis	FReD	flower	UV-Yellow	u-b-g+r+	0.073	0.007	0.562	0.917
Calicotome villosa	FReD	flower	UV+Yellow	u+b-g+r+	0.108	0.015	0.752	0.916
Calliandra harrisii	JBRJ	flower	UV+Red	u+b-g-r+	0.335	0.250	0.174	0.883
Calluna vulgaris	FReD	flower	UV-Pink	u-b+g-r+	0.061	0.433	0.367	0.664
Camellia japonica	JBRJ	flower	UV+Pink	u+b+g-r+	0.390	0.344	0.111	0.878
Camellia sinensis	JBRJ	calyx	UV-White	u-b+g+r+	0.023	0.628	0.920	0.854
Camoensia scandens	JBRJ	flower	UV+White	u+b+g+r+	0.114	0.874	0.790	0.817
Campanula latifolia	FReD	flower	UV+Blue	u+b+g-r-	0.274	0.600	0.232	0.510
Campanula rapunculoides	FReD	flower	UV+White	u+b+g+r+	0.217	0.666	0.439	0.690
Campanula trachelium	FReD	flower	UV+Blue	u+b+g-r-	0.206	0.692	0.222	0.501
Canistrum aurantiacum	JBRJ	bract	UV-Red	u-b-g-r+	0.012	0.036	0.028	0.853
Canistrum fragrans	JBRJ	bract	UV-Black	u-b-g-r-	0.008	0.144	0.211	0.599
Canistrum superbum	JBRJ	bract	UV+Black	u+b-g-r-	0.112	0.172	0.211	0.542
Canna indica	JBRJ	flower	UV+Yellow	u+b-g+r+	0.139	0.156	0.444	0.832
Capsella bursa-pastoris	FReD	flower	UV+White	u+b+g+r+	0.123	0.794	0.869	0.869
Carapa guianensis	JBRJ	flower	UV-White	u-b+g+r+	0.039	0.347	0.731	0.736
Cardamine pratensis	FReD	flower	UV-White	u-b+g+r+	0.058	0.770	0.709	0.908
Cascabela thevetia	JBRJ	flower	UV-Red	u-b-g-r+	0.060	0.106	0.231	0.899
Centaurea aegyptiaca	FReD	flower	UV+White	u+b+g+r+	0.109	0.493	0.717	0.867
Centaurea ammocyanus	FReD	flower	UV+Pink	u+b+g-r+	0.118	0.571	0.386	0.827
Centaurea pallescens	FReD	flower	UV-White	u-b+g+r+	0.011	0.314	0.816	0.932
Cerastium holosteoides	FReD	flower	UV-White	u-b+g+r+	0.060	0.875	0.979	0.984
Cercis siliquastrum	FReD	flower	UV-Pink	u-b+g-r+	0.022	0.543	0.317	0.814
Chelidonium majus	FReD	flower	UV+Yellow	u+b-g+r+	0.451	0.013	0.681	0.985
Cirsium oleraceum	FReD	flower	UV-White	u-b+g+r+	0.039	0.403	0.810	0.963
Cistus incanus	FReD	calyx	UV-Yellow	u-b-g+r+	0.016	0.055	0.719	0.933
Cistus salviifolius	FReD	calyx	UV-Yellow	u-b-g+r+	0.017	0.067	0.676	0.899

Cleistocactus straussii	JBRJ	flower	UV-Red	u-b-g-r+	0.057	0.182	0.021	0.694
Clerodendrum quadriloculare	JBRJ	calyx	UV-Black	u-b-g-r-	0.003	0.061	0.069	0.449
Clerodendrum thomsoniae	JBRJ	calyx	UV-White	u-b+g+r+	0.002	0.441	0.771	0.849
Clinopodium vulgare	FReD	flower	UV-White	u-b+g+r+	0.023	0.661	0.930	0.945
Colutea istria	FReD	flower	UV+Yellow	u+b-g+r+	0.218	0.022	0.636	0.870
Combretum indicum	JBRJ	flower	UV-Red	u-b-g-r+	0.005	0.172	0.175	0.817
Combretum paniculatum	JBRJ	flower	UV-Red	u-b-g-r+	0.060	0.060	0.194	0.833
Combretum rotundifolium	JBRJ	flower	UV-Red	u-b-g-r+	0.005	0.091	0.325	0.769
Congea tomentosa	JBRJ	bract	UV-White	u-b+g+r+	0.037	0.600	0.577	0.841
Corylus avellana	FReD	flower	UV-Yellow	u-b-g+r+	0.015	0.194	0.551	0.675
Crataegus azarolus	FReD	flower	UV-White	u-b+g+r+	0.024	0.661	0.891	0.906
Crepis aspera	FReD	calyx	UV-Yellow	u-b-g+r+	0.049	0.008	0.695	0.931
Crepis hierosolymitana	FReD	calyx	UV+Yellow	u+b-g+r+	0.137	0.025	0.777	0.885
Crepis palaestina	FReD	calyx	UV+Yellow	u+b-g+r+	0.181	0.025	0.820	0.898
Crepis sancta	FReD	calyx	UV-Yellow	u-b-g+r+	0.048	0.095	0.762	0.911
Crinum asiaticum	JBRJ	flower	UV-White	u-b+g+r+	0.041	0.879	0.909	0.897
Crinum latifolium	JBRJ	flower	UV+White	u+b+g+r+	0.170	0.925	0.842	0.840
Crupina crupinastrum	FReD	calyx	UV-White	u-b+g+r+	0.034	0.458	0.459	0.759
Cuphea racemosa	JBRJ	flower	UV-Red	u-b-g-r+	0.084	0.271	0.137	0.607
Cyclamen persicum	FReD	flower	UV-White	u-b+g+r+	0.016	0.632	0.592	0.867
Cynoglossum officinale	FReD	flower	UV+Black	u+b-g-r-	0.203	0.101	0.031	0.366
Cyrtopodium flavum	JBRJ	flower	UV+Yellow	u+b-g+r+	0.369	0.075	0.777	0.843
Dahlstedtia pinnata	JBRJ	flower	UV+Pink	u+b+g-r+	0.113	0.318	0.254	0.725
Deuterocohnia meziana	JBRJ	bract	UV-Red	u-b-g-r+	0.006	0.084	0.203	0.858
Dianthus carthusianorum	FReD	flower	UV-Red	u-b-g-r+	0.016	0.254	0.102	0.749
Dietes bicolor	JBRJ	flower	UV-White	u-b+g+r+	0.005	0.464	0.704	0.921
Diplotaxis harra	FReD	flower	UV-Yellow	u-b-g+r+	0.044	0.143	0.893	0.924
Dracaena reflexa	JBRJ	flower	UV-Black	u-b-g-r-	0.019	0.173	0.295	0.576
Duranta erecta	JBRJ	flower	UV-Pink	u-b+g-r+	0.036	0.642	0.333	0.695
Dyckia brevifolia	JBRJ	flower	UV-Yellow	u-b-g+r+	0.030	0.047	0.630	0.725
Dyckia encholirioides	JBRJ	flower	UV-Yellow	u-b-g+r+	0.024	0.063	0.530	0.847
Echeveria sp.	JBRJ	flower	UV-Yellow	u-b-g+r+	0.072	0.193	0.889	0.967
Echium angustifolium	FReD	flower	UV-Black	u-b-g-r-	0.071	0.030	0.021	0.423
Echium rauwolfii	FReD	flower	UV+Pink	u+b+g-r+	0.253	0.394	0.302	0.727
Emilia sonchifolia	FReD	flower	UV-Red	u-b-g-r+	0.034	0.151	0.122	0.774
Encyclia cordigera	JBRJ	flower	UV+Black	u+b-g-r-	0.331	0.187	0.069	0.159

Epilobium angustifolium	FReD	flower	UV-Pink	u-b+g-r+	0.037	0.340	0.134	0.732
Epilobium parviflorum	FReD	flower	UV-White	u-b+g+r+	0.056	0.632	0.474	0.854
Episcia cupreata	JBRJ	flower	UV-Red	u-b-g-r+	0.016	0.023	0.080	0.897
Erigeron canadensis	FReD	flower	UV-White	u-b+g+r+	0.097	0.885	0.980	0.980
Erucaria pinnata	FReD	flower	UV-White	u-b+g+r+	0.039	0.763	0.930	0.935
Erysimum cheiranthoides	FReD	flower	UV+Yellow	u+b-g+r+	0.128	0.084	0.804	0.817
Erythrina falcata	JBRJ	flower	UV-Red	u-b-g-r+	0.041	0.067	0.186	0.845
Erythrina fusca	JBRJ	flower	UV-Yellow	u-b-g+r+	0.096	0.268	0.651	0.668
Erythrina speciosa	JBRJ	flower	UV-Red	u-b-g-r+	0.032	0.033	0.119	0.878
Etlingera elatior	JBRJ	flower	UV-Red	u-b-g-r+	0.079	0.199	0.218	0.839
Eugenia brasiliensis	JBRJ	flower	UV-White	u-b+g+r+	0.011	0.497	0.813	0.758
Eugenia uniflora	JBRJ	flower	UV-Yellow	u-b-g+r+	0.000	0.117	0.446	0.857
Euphorbia hierosolymitana	FReD	flower	UV-Yellow	u-b-g+r+	0.020	0.041	0.637	0.618
Ficaria verna	FReD	flower	UV+Yellow	u+b-g+r+	0.496	0.038	0.852	0.938
Filipendula ulmaria	FReD	flower	UV-White	u-b+g+r+	0.060	0.656	0.988	0.976
Fragaria viridis	FReD	flower	UV-White	u-b+g+r+	0.016	0.586	0.902	0.940
Galeopsis bifida	FReD	flower	UV-Pink	u-b+g-r+	0.028	0.303	0.129	0.670
Galeopsis pubescens	FReD	flower	UV+Blue	u+b+g-r-	0.125	0.397	0.196	0.445
Galeopsis tetrahit	FReD	flower	UV-White	u-b+g+r+	0.057	0.623	0.916	0.956
Galium verum	FReD	flower	UV-Yellow	u-b-g+r+	0.024	0.070	0.686	0.858
Gasteria pulchra	JBRJ	flower	UV-White	u-b+g+r+	0.016	0.422	0.668	0.878
Gazania heterochaeta	FReD	flower	UV-Red	u-b-g-r+	0.012	0.100	0.300	0.658
Geranium sylvaticum	FReD	flower	UV+Blue	u+b+g-r-	0.305	0.382	0.051	0.561
Geum rivale	FReD	flower	UV+White	u+b+g+r+	0.310	0.390	0.583	0.789
Glechoma hederacea	FReD	flower	UV-Blue	u-b+g-r-	0.025	0.439	0.301	0.583
Gliricidia sepium	JBRJ	flower	UV-White	u-b+g+r+	0.018	0.422	0.941	0.886
Gmelina arborea	JBRJ	flower	UV-Black	u-b-g-r-	0.009	0.058	0.121	0.576
Gmelina asiatica	JBRJ	flower	UV-Yellow	u-b-g+r+	0.052	0.099	0.745	0.893
Gomesa flexuosa	JBRJ	flower	UV+Yellow	u+b-g+r+	0.283	0.049	0.873	0.980
Guarea guidonia	JBRJ	flower	UV-White	u-b+g+r+	0.080	0.514	0.838	0.879
Guazuma ulmifolia	JBRJ	flower	UV-Red	u-b-g-r+	0.002	0.000	0.360	0.767
Gustavia augusta	JBRJ	flower	UV-Pink	u-b+g-r+	0.008	0.414	0.402	0.925
Gymnocarpos decandrus	FReD	flower	UV-Black	u-b-g-r-	0.032	0.165	0.367	0.492
Gypsophila arabica	FReD	flower	UV+White	u+b+g+r+	0.314	0.584	0.766	0.811
Habranthus robustus	JBRJ	flower	UV-White	u-b+g+r+	0.016	0.621	0.819	0.921
Handroanthus heptaphyllus	JBRJ	flower	UV-Pink	u-b+g-r+	0.029	0.342	0.203	0.839

Haworthia attenuata	JBRJ	flower	UV-Cyan	u-b+g+r-	0.095	0.363	0.647	0.530
Hedychium coccineum	JBRJ	flower	UV-Red	u-b-g-r+	0.005	0.092	0.306	0.947
Hedypnois rhagadioloides	FReD	flower	UV-Yellow	u-b-g+r+	0.055	0.014	0.666	0.884
Helianthemum ventosum	FReD	flower	UV+Yellow	u+b-g+r+	0.305	0.047	0.800	0.879
Helianthemum vesicarium	FReD	flower	UV-White	u-b+g+r+	0.038	0.574	0.402	0.862
Heliconia metallica	JBRJ	flower	UV-Red	u-b-g-r+	0.099	0.139	0.151	0.876
Heliconia pendula	JBRJ	bract	UV-Red	u-b-g-r+	0.023	0.065	0.163	0.824
Heliconia psittacorum	JBRJ	bract	UV-Red	u-b-g-r+	0.015	0.063	0.261	0.773
Helicteres brevispira	JBRJ	flower	UV-Black	u-b-g-r-	0.008	0.038	0.116	0.465
Hepatica nobilis	FReD	flower	UV-Pink	u-b+g-r+	0.088	0.572	0.224	0.821
Hesperis pendula	FReD	flower	UV-Green	u-b-g+r-	0.017	0.101	0.470	0.494
Heterotaxis sessilis	JBRJ	flower	UV-Yellow	u-b-g+r+	0.000	0.000	0.549	0.720
Hibiscus rosa-sinensis	FReD	flower	UV-Red	u-b-g-r+	0.098	0.133	0.104	0.774
Hibiscus schizopetalus	JBRJ	flower	UV-Red	u-b-g-r+	0.037	0.048	0.048	0.900
Hibiscus tiliaceus	JBRJ	flower	UV-Yellow	u-b-g+r+	0.006	0.222	0.645	0.951
Hieracium laevigatum	FReD	flower	UV+Yellow	u+b-g+r+	0.267	0.027	0.806	0.971
Hieracium sabaudum	FReD	flower	UV+Yellow	u+b-g+r+	0.144	0.029	0.726	0.921
Hohenbergia stellata	JBRJ	bract	UV-Red	u-b-g-r+	0.030	0.190	0.137	0.926
Holosteum umbellatum	FReD	flower	UV-White	u-b+g+r+	0.051	0.788	0.886	0.923
Hylotelephium maximum	FReD	flower	UV-White	u-b+g+r+	0.076	0.538	0.905	0.965
Hyoscyamus aureus	FReD	flower	UV-Yellow	u-b-g+r+	0.010	0.128	0.761	0.675
Hypoxis decumbens	JBRJ	flower	UV+Yellow	u+b-g+r+	0.406	0.033	0.789	0.942
Impatiens walleriana	JBRJ	flower	UV+Red	u+b-g-r+	0.133	0.136	0.281	0.961
Ipomoea purpurea	JBRJ	flower	UV-Pink	u-b+g-r+	0.032	0.380	0.155	0.715
Ipomoea squamosa	FReD	flower	UV+Pink	u+b+g-r+	0.104	0.539	0.244	0.704
Isatis lusitanica	FReD	flower	UV+Yellow	u+b-g+r+	0.194	0.041	0.796	0.869
Ixora coccinea	JBRJ	flower	UV-Yellow	u-b-g+r+	0.043	0.186	0.637	0.924
Jacobaea vulgaris	FReD	flower	UV+Yellow	u+b-g+r+	0.125	0.018	0.712	0.811
Jacquemontia sp.	JBRJ	flower	UV+Blue	u+b+g-r-	0.186	0.698	0.087	0.335
Justicia brandegeeana	FReD	bract	UV+Yellow	u+b-g+r+	0.163	0.028	0.497	0.604
Justicia gendarussa	JBRJ	flower	UV-Red	u-b-g-r+	0.003	0.221	0.211	0.691
Knautia arvensis	FReD	flower	UV-White	u-b+g+r+	0.040	0.524	0.459	0.802
Knautia dipsacifolia	FReD	flower	UV-White	u-b+g+r+	0.031	0.460	0.435	0.726
Kopsia fruticosa	JBRJ	flower	UV-Pink	u-b+g-r+	0.039	0.423	0.309	0.909
Lamium album	FReD	flower	UV-White	u-b+g+r+	0.021	0.576	0.940	0.964
Lamium galeobdolon	FReD	flower	UV+Yellow	u+b-g+r+	0.164	0.178	0.847	0.964

Lamium garganicum	FReD	flower	UV-White	u-b+g+r+	0.074	0.558	0.573	0.779
Lantana camara	JBRJ	flower	UV-Red	u-b-g-r+	0.003	0.021	0.427	0.959
Lapsana communis	FReD	flower	UV-Yellow	u-b-g+r+	0.053	0.079	0.797	0.799
Lathyrus blepharicarpus	FReD	flower	UV+Red	u+b-g-r+	0.381	0.049	0.228	0.728
Lathyrus gorgoni	FReD	flower	UV+Red	u+b-g-r+	0.387	0.035	0.324	0.765
Lathyrus pratensis	FReD	flower	UV-Yellow	u-b-g+r+	0.038	0.055	0.648	0.831
Lathyrus vernus	FReD	flower	UV-Pink	u-b+g-r+	0.074	0.403	0.253	0.655
Launaea angustifolia	FReD	flower	UV-Yellow	u-b-g+r+	0.082	0.011	0.658	0.908
Launaea mucronata	FReD	flower	UV+Yellow	u+b-g+r+	0.238	0.023	0.720	0.866
Launaea nudicaulis	FReD	calyx	UV-Yellow	u-b-g+r+	0.016	0.020	0.607	0.930
Lebeckia halenbergensis	FReD	flower	UV-Yellow	u-b-g+r+	0.046	0.015	0.568	0.702
Leontodon laciniatus	FReD	flower	UV-Yellow	u-b-g+r+	0.044	0.015	0.623	0.947
Leontodon tuberosus	FReD	flower	UV+Yellow	u+b-g+r+	0.167	0.024	0.753	0.920
Leopoldia comosa	FReD	flower	UV+Blue	u+b+g-r-	0.156	0.482	0.155	0.474
Leopoldia longipes	FReD	flower	UV-Green	u-b-g+r-	0.066	0.236	0.538	0.534
Lepidium draba	FReD	flower	UV-White	u-b+g+r+	0.056	0.774	0.963	0.948
Liatris spicata	FReD	flower	UV-Pink	u-b+g-r+	0.045	0.474	0.136	0.756
Limodorum abortivum	FReD	flower	UV+Pink	u+b+g-r+	0.154	0.380	0.277	0.608
Linaria vulgaris	FReD	flower	UV-White	u-b+g+r+	0.019	0.359	0.714	0.743
Linum pubescens	FReD	flower	UV+Red	u+b-g-r+	0.485	0.225	0.133	0.665
Lobelia anceps	JBRJ	flower	UV-Pink	u-b+g-r+	0.006	0.494	0.389	0.716
Lotus longesiliquosus	FReD	flower	UV-Yellow	u-b-g+r+	0.046	0.010	0.509	0.887
Lycium shawii	FReD	flower	UV-White	u-b+g+r+	0.032	0.372	0.625	0.806
Malouetia arborea	JBRJ	flower	UV-White	u-b+g+r+	0.011	0.610	0.955	0.967
Malvaviscus arboreus	JBRJ	flower	UV-Red	u-b-g-r+	0.018	0.097	0.224	0.946
Mammillaria bombycina	JBRJ	flower	UV-Pink	u-b+g-r+	0.097	0.578	0.265	0.767
Mammillaria elongata	JBRJ	flower	UV-White	u-b+g+r+	0.015	0.354	0.842	0.878
Maranta leuconeura	JBRJ	flower	UV-White	u-b+g+r+	0.017	0.592	0.768	0.960
Matricaria aurea	FReD	flower	UV-Green	u-b-g+r-	0.022	0.042	0.588	0.569
Megaskepasma erythrochlamys	JBRJ	bract	UV-Red	u-b-g-r+	0.002	0.102	0.074	0.708
Melampyrum pratense	FReD	flower	UV-Yellow	u-b-g+r+	0.038	0.097	0.636	0.840
Melampyrum sylvaticum	FReD	flower	UV-Yellow	u-b-g+r+	0.014	0.020	0.578	0.816
Mentha aquatica	FReD	flower	UV-White	u-b+g+r+	0.051	0.671	0.548	0.823
Merremia dissecta	JBRJ	flower	UV+White	u+b+g+r+	0.174	0.793	0.960	0.965
Mesembryanthemum cryptanthum	FReD	flower	UV-White	u-b+g+r+	0.046	0.543	0.912	0.959
Mesembryanthemum nodiflorum	FReD	flower	UV-White	u-b+g+r+	0.060	0.474	0.933	0.935

Moehringia trinervia	FReD	flower	UV-White	u-b+g+r+	0.056	0.734	0.975	0.957
Moricandia nitens	FReD	flower	UV+Pink	u+b+g-r+	0.270	0.572	0.338	0.661
Mucuna bennettii	JBRJ	flower	UV-Red	u-b-g-r+	0.055	0.097	0.306	0.829
Musa ornata	JBRJ	bract	UV-Pink	u-b+g-r+	0.007	0.530	0.232	0.823
Mussaenda philippica	JBRJ	calyx	UV-Yellow	u-b-g+r+	0.002	0.040	0.723	0.934
Myosotis alpestris	FReD	flower	UV-Blue	u-b+g-r-	0.058	0.698	0.353	0.555
Myosotis decumbens	FReD	flower	UV-White	u-b+g+r+	0.044	0.669	0.656	0.802
Myosotis stricta	FReD	flower	UV+White	u+b+g+r+	0.130	0.701	0.426	0.609
Myosotis vestergrenii	FReD	flower	UV-Blue	u-b+g-r-	0.037	0.600	0.319	0.541
Nanorrhinum scoparium	FReD	flower	UV-White	u-b+g+r+	0.056	0.324	0.842	0.871
Neomarica candida	JBRJ	flower	UV-White	u-b+g+r+	0.015	0.821	0.964	0.963
Neoregelia cruenta	JBRJ	flower	UV-Cyan	u-b+g+r-	0.097	0.421	0.571	0.597
Nopalea cochenillifera	JBRJ	flower	UV-Red	u-b-g-r+	0.071	0.121	0.191	0.821
Ocimum basilicum	JBRJ	flower	UV+White	u+b+g+r+	0.114	0.798	0.811	0.903
Odontonema tubaeforme	JBRJ	flower	UV-Red	u-b-g-r+	0.067	0.182	0.132	0.791
Onobrychis crista-galli	FReD	flower	UV-Pink	u-b+g-r+	0.039	0.501	0.315	0.795
Ononis natrix	FReD	flower	UV-Yellow	u-b-g+r+	0.056	0.011	0.686	0.933
Orchis italica	FReD	flower	UV-White	u-b+g+r+	0.023	0.534	0.473	0.751
Origanum vulgare	FReD	calyx	UV-Red	u-b-g-r+	0.093	0.285	0.310	0.614
Oxalis debilis	JBRJ	flower	UV-Pink	u-b+g-r+	0.016	0.456	0.073	0.857
Oxytropis jacquinii	FReD	flower	UV-Blue	u-b+g-r-	0.061	0.412	0.224	0.491
Oxytropis neglecta	FReD	flower	UV+Pink	u+b+g-r+	0.219	0.662	0.355	0.725
Pachystachys lutea	JBRJ	bract	UV-Yellow	u-b-g+r+	0.058	0.051	0.608	0.820
Pachystachys spicata	JBRJ	flower	UV-Red	u-b-g-r+	0.007	0.020	0.046	0.821
Papaver hybridum	FReD	flower	UV+Black	u+b-g-r-	0.145	0.039	0.040	0.573
Papaver rhoeas	FReD	flower	UV+Red	u+b-g-r+	0.164	0.021	0.079	0.787
Papaver umbonatum	FReD	flower	UV+Red	u+b-g-r+	0.112	0.047	0.052	0.644
Parnassia palustris	FReD	flower	UV-White	u-b+g+r+	0.024	0.635	0.904	0.910
Pavonia alnifolia	JBRJ	flower	UV-Black	u-b-g-r-	0.071	0.174	0.351	0.600
Pedicularis comosa	FReD	flower	UV-Yellow	u-b-g+r+	0.028	0.294	0.669	0.753
Pedicularis recutita	FReD	flower	UV-Black	u-b-g-r-	0.066	0.226	0.301	0.517
Phalaenopsis amabilis	JBRJ	flower	UV+White	u+b+g+r+	0.117	0.673	0.857	0.865
Phlomoides laciniata	FReD	flower	UV-White	u-b+g+r+	0.044	0.433	0.826	0.902
Phyllodoce caerulea	FReD	flower	UV-White	u-b+g+r+	0.031	0.460	0.435	0.726
Phyteuma betonicifolium	FReD	flower	UV+Blue	u+b+g-r-	0.126	0.307	0.028	0.361
Phyteuma hedraianthifolium	FReD	flower	UV+Blue	u+b+g-r-	0.160	0.543	0.259	0.555

Phyteuma hemisphaericum	FReD	flower	UV+Pink	u+b+g-r+	0.104	0.337	0.254	0.608
Phyteuma nigrum	FReD	flower	UV+Black	u+b-g-r-	0.115	0.144	0.119	0.251
Picris longirostris	FReD	flower	UV-Yellow	u-b-g+r+	0.046	0.020	0.741	0.924
Pilosella officinarum	FReD	flower	UV+Yellow	u+b-g+r+	0.235	0.021	0.774	0.963
Pinguicula alpina	FReD	flower	UV-White	u-b+g+r+	0.025	0.554	0.939	0.961
Pitcairnia staminea	JBRJ	flower	UV-Red	u-b-g-r+	0.024	0.175	0.216	0.930
Platanthera clavellata	FReD	flower	UV-Black	u-b-g-r-	0.013	0.220	0.099	0.549
Potentilla heptaphylla	FReD	flower	UV+Yellow	u+b-g+r+	0.142	0.020	0.734	0.867
Potentilla reptans	FReD	flower	UV+Yellow	u+b-g+r+	0.172	0.026	0.648	0.727
Premna cordifolia	JBRJ	flower	UV+Green	u+b-g+r-	0.183	0.289	0.659	0.520
Primula elatior	FReD	flower	UV-Yellow	u-b-g+r+	0.017	0.048	0.746	0.965
Prunus padus	FReD	flower	UV-White	u-b+g+r+	0.007	0.660	0.863	0.881
Pseudolaelia corcovadensis	JBRJ	flower	UV-White	u-b+g+r+	0.084	0.683	0.431	0.862
Pulicaria incisa	FReD	flower	UV-Yellow	u-b-g+r+	0.020	0.027	0.786	0.965
Pulmonaria mollis	FReD	flower	UV+Blue	u+b+g-r-	0.377	0.327	0.045	0.397
Pulmonaria obscura	FReD	flower	UV+Blue	u+b+g-r-	0.423	0.401	0.056	0.418
Quesnelia arvensis	JBRJ	bract	UV-Pink	u-b+g-r+	0.030	0.468	0.302	0.950
Quesnelia liboniana	JBRJ	flower	UV-Black	u-b-g-r-	0.094	0.117	0.080	0.567
Quesnelia sp.	JBRJ	flower	UV+Blue	u+b+g-r-	0.145	0.320	0.276	0.365
Randia sp.	JBRJ	flower	UV-White	u-b+g+r+	0.036	0.834	0.940	0.945
Ranunculus acris	FReD	flower	UV-Yellow	u-b-g+r+	0.090	0.014	0.657	0.945
Ranunculus asiaticus	FReD	flower	UV-Red	u-b-g-r+	0.065	0.292	0.107	0.678
Ranunculus marginatus	FReD	flower	UV+Green	u+b-g+r-	0.151	0.027	0.472	0.522
Ranunculus millefolius	FReD	flower	UV+Yellow	u+b-g+r+	0.272	0.037	0.752	0.740
Renanthera coccinea	JBRJ	flower	UV-Red	u-b-g-r+	0.006	0.000	0.000	0.687
Retama raetam	FReD	flower	UV-White	u-b+g+r+	0.053	0.730	0.834	0.873
Rhagadiolus stellatus	FReD	calyx	UV-Yellow	u-b-g+r+	0.098	0.018	0.770	0.929
Rivina humilis	JBRJ	flower	UV-White	u-b+g+r+	0.011	0.464	0.608	0.815
Russelia equisetiformis	JBRJ	flower	UV-Red	u-b-g-r+	0.001	0.032	0.122	0.915
Ruta chalepensis	FReD	flower	UV-Green	u-b-g+r-	0.019	0.032	0.602	0.585
Salvia dominica	FReD	flower	UV-White	u-b+g+r+	0.048	0.708	0.863	0.873
Salvia fruticosa	FReD	flower	UV-White	u-b+g+r+	0.082	0.626	0.509	0.758
Salvia hierosolymitana	FReD	flower	UV-White	u-b+g+r+	0.021	0.472	0.472	0.797
Salvia lanigera	FReD	flower	UV-Blue	u-b+g-r-	0.071	0.477	0.174	0.541
Salvia splendens	JBRJ	flower	UV-Red	u-b-g-r+	0.008	0.020	0.080	0.811
Sanchezia oblonga	FReD	calyx	UV+Black	u+b-g-r-	0.111	0.073	0.125	0.549

Sanchezia speciosa	JBRJ	bract	UV-Yellow	u-b-g+r+	0.020	0.075	0.553	0.930
Sansevieria cylindrica	JBRJ	flower	UV-Yellow	u-b-g+r+	0.015	0.162	0.823	0.774
Saraca thaipingensis	JBRJ	flower	UV+Yellow	u+b-g+r+	0.332	0.147	0.616	0.918
Satureja thymbra	FReD	flower	UV-Pink	u-b+g-r+	0.072	0.451	0.389	0.771
Scandix pecten-veneris	FReD	flower	UV-White	u-b+g+r+	0.031	0.469	0.692	0.644
Scilla hyacinthoides	FReD	flower	UV-White	u-b+g+r+	0.078	0.714	0.464	0.675
Scorzonera papposa	FReD	flower	UV+White	u+b+g+r+	0.136	0.519	0.401	0.705
Scorzoneroides autumnalis	FReD	flower	UV-Yellow	u-b-g+r+	0.080	0.104	0.782	0.981
Scrophularia xanthoglossa	FReD	flower	UV-Black	u-b-g-r-	0.030	0.105	0.245	0.429
Securigera varia	FReD	flower	UV-White	u-b+g+r+	0.030	0.699	0.861	0.931
Senecio glaucus	FReD	calyx	UV-Yellow	u-b-g+r+	0.020	0.040	0.628	0.792
Senecio vernalis	FReD	calyx	UV-Yellow	u-b-g+r+	0.034	0.095	0.752	0.919
Silene acaulis	FReD	flower	UV-Pink	u-b+g-r+	0.024	0.566	0.391	0.823
Silene aegyptiaca	FReD	flower	UV+White	u+b+g+r+	0.144	0.606	0.453	0.828
Silene flos-cuculi	FReD	flower	UV+Pink	u+b+g-r+	0.115	0.513	0.324	0.815
Silene nutans	FReD	flower	UV-White	u-b+g+r+	0.040	0.733	0.873	0.889
Sinapis arvensis	FReD	flower	UV+Yellow	u+b-g+r+	0.390	0.042	0.797	0.857
Sinapis incana	FReD	flower	UV+Yellow	u+b-g+r+	0.349	0.049	0.815	0.868
Smilax sp.	JBRJ	flower	UV-Green	u-b-g+r-	0.020	0.171	0.686	0.513
Sobralia yauaperyensis	JBRJ	flower	UV-White	u-b+g+r+	0.031	0.811	0.812	0.855
Solidago canadensis	FReD	flower	UV-Yellow	u-b-g+r+	0.031	0.049	0.693	0.947
Sonchus oleraceus	FReD	flower	UV+Yellow	u+b-g+r+	0.270	0.081	0.677	0.733
Spathoglottis unguiculata	JBRJ	flower	UV-Red	u-b-g-r+	0.025	0.266	0.025	0.709
Stachys recta	FReD	flower	UV-White	u-b+g+r+	0.033	0.427	0.859	0.895
Stachys sylvatica	FReD	flower	UV-Black	u-b-g-r-	0.084	0.209	0.185	0.584
Stachytarpheta speciosa	JBRJ	flower	UV-Blue	u-b+g-r-	0.029	0.473	0.204	0.391
Stellaria holostea	FReD	flower	UV+White	u+b+g+r+	0.112	0.869	0.970	0.979
Stellaria palustris	FReD	flower	UV-White	u-b+g+r+	0.084	0.832	0.950	0.953
Stifftia chrysantha	JBRJ	flower	UV-Yellow	u-b-g+r+	0.025	0.143	0.475	0.881
Stromanthe sp.	JBRJ	bract	UV-Red	u-b-g-r+	0.002	0.159	0.178	0.848
Swartzia simplex	JBRJ	flower	UV+Yellow	u+b-g+r+	0.437	0.049	0.590	0.921
Symphoricarpos albus	FReD	flower	UV-White	u-b+g+r+	0.079	0.408	0.411	0.877
Symphytum brachycalyx	FReD	flower	UV-White	u-b+g+r+	0.020	0.664	0.908	0.928
Syzygium malaccense	JBRJ	flower	UV+Pink	u+b+g-r+	0.207	0.325	0.190	0.874
Tacinga palmadora	JBRJ	flower	UV-Red	u-b-g-r+	0.016	0.026	0.246	0.926
Tamarix nilotica	FReD	flower	UV-White	u-b+g+r+	0.051	0.561	0.782	0.905

Tanaecium pyramidatum	JBRJ	flower	UV-White	u-b+g+r+	0.043	0.589	0.402	0.858
Taraxacum officinale	FReD	calyx	UV-Yellow	u-b-g+r+	0.048	0.012	0.473	0.935
Thalia geniculata	JBRJ	flower	UV-White	u-b+g+r+	0.035	0.545	0.566	0.755
Thunbergia erecta	JBRJ	flower	UV+Blue	u+b+g-r-	0.124	0.516	0.076	0.493
Thunbergia mysorensis	JBRJ	flower	UV+Yellow	u+b-g+r+	0.180	0.152	0.665	0.915
Thymus serpyllum	FReD	flower	UV-Pink	u-b+g-r+	0.048	0.397	0.243	0.702
Tillandsia polystachia	JBRJ	flower	UV-Pink	u-b+g-r+	0.013	0.314	0.399	0.667
Tillandsia stricta	JBRJ	bract	UV-Pink	u-b+g-r+	0.013	0.327	0.198	0.884
Tillandsia tenuifolia	JBRJ	flower	UV-Blue	u-b+g-r-	0.020	0.650	0.196	0.375
Tridax procumbens	JBRJ	flower	UV-White	u-b+g+r+	0.002	0.524	0.973	0.958
Trifolium clypeatum	FReD	flower	UV-White	u-b+g+r+	0.012	0.624	0.870	0.901
Trifolium repens	FReD	flower	UV-White	u-b+g+r+	0.072	0.604	0.907	0.916
Trifolium resupinatum	FReD	flower	UV-Pink	u-b+g-r+	0.045	0.374	0.260	0.747
Trifolium stellatum	FReD	flower	UV-Pink	u-b+g-r+	0.014	0.314	0.353	0.815
Trigonella caelesyriaca	FReD	flower	UV-Yellow	u-b-g+r+	0.038	0.020	0.791	0.888
Trigonella kotschyi	FReD	flower	UV-Yellow	u-b-g+r+	0.053	0.014	0.754	0.880
Tripleurospermum auriculatum	FReD	flower	UV-Yellow	u-b-g+r+	0.021	0.043	0.609	0.744
Tussilago farfara	FReD	calyx	UV-Yellow	u-b-g+r+	0.031	0.115	0.704	0.919
Urospermum picroides	FReD	flower	UV-Yellow	u-b-g+r+	0.013	0.093	0.844	0.941
Ursinia cakilefolia	FReD	flower	UV+Red	u+b-g-r+	0.283	0.008	0.320	0.758
Vaccinium vitis-idaea	FReD	flower	UV-White	u-b+g+r+	0.039	0.521	0.814	0.945
Vellozia candida	JBRJ	flower	UV-White	u-b+g+r+	0.022	0.781	0.952	0.933
Verbascum densiflorum	FReD	flower	UV+Yellow	u+b-g+r+	0.251	0.040	0.837	0.948
Verbascum lychnitis	FReD	flower	UV+Yellow	u+b-g+r+	0.134	0.045	0.726	0.799
Veronica arvensis	FReD	flower	UV-White	u-b+g+r+	0.085	0.733	0.652	0.788
Veronica chamaedrys	FReD	flower	UV+Blue	u+b+g-r-	0.232	0.596	0.158	0.432
Veronica prostrata	FReD	flower	UV+Blue	u+b+g-r-	0.279	0.628	0.276	0.540
Veronica spicata	FReD	flower	UV+Blue	u+b+g-r-	0.123	0.390	0.193	0.446
Viburnum opulus	FReD	flower	UV-White	u-b+g+r+	0.030	0.706	0.896	0.902
Vicia hybrida	FReD	flower	UV-Yellow	u-b-g+r+	0.016	0.190	0.800	0.897
Vicia sativa	FReD	flower	UV-Pink	u-b+g-r+	0.031	0.401	0.303	0.697
Vigna unguiculata	JBRJ	flower	UV-Pink	u-b+g-r+	0.006	0.433	0.238	0.673
Vincetoxicum hirundinaria	FReD	flower	UV+White	u+b+g+r+	0.119	0.548	0.915	0.945
Vriesea neoglutinosa	JBRJ	bract	UV-Red	u-b-g-r+	0.000	0.009	0.029	0.627
Youngia japonica	JBRJ	flower	UV+Yellow	u+b-g+r+	0.211	0.161	0.809	0.912
Yucca aloifolia	JBRJ	flower	UV-White	u-b+g+r+	0.075	0.764	0.960	0.968

Zilla spinosa	FReD	flower	UV-White	u-b+g+r+	0.088	0.654	0.453	0.803

Table S3: Visitors' data used for bee contrast differences analysis and their respective references. **System** refers to the groups assigned to each species accordingly.

**Species**	System	Visitors	Reference
Aaronsohnia factorovskyi	bee+insect	bee-beetle-fly	FReD [Arnold et al. (2008)]
Acanthus montanus	bee	bee	Abdullahi et al. (2011)
Achillea santolina	bee	bee	FReD [Arnold et al. (2008)]
Aconitum septentrionale	bee	bee	FReD [Arnold et al. (2008)]
Aechmea aquilega	bird	bird	pers. obs.*
Aechmea bromeliifolia	bird	bird	Sazima and Sazima (1999); pers. obs.*
Aechmea eurycorymbus	bird	bird	pers. obs.*
Aechmea pectinata	bird	bird	Canela and Sazima (2003); pers. obs.*
Aechmea purpureorosea	bird	bird	pers. obs.*
Aechmea sp.	bird	bird	pers. obs.*
Aechmea tomentosa	bird	bird	Lopes et al. (2002)
Agrimonia eupatoria	bee+insect	bee-butterfly-fly	FReD [Arnold et al. (2008)]
Ainsworthia trachycarpa	insect	beetle-fly	FReD [Arnold et al. (2008)]
Ajuga chamaepitys	bee	bee	FReD [Arnold et al. (2008)]
Ajuga genevensis	bee+insect	bee-butterfly	FReD [Arnold et al. (2008)]
Ajuga pyramidalis	bee	bee	FReD [Arnold et al. (2008)]
Ajuga reptans	bee	bee	FReD [Arnold et al. (2008)]
Alcea acaulis	bee	bee	FReD [Arnold et al. (2008)]
Alcea dissecta	bee	bee	FReD [Arnold et al. (2008)]
Alectis ciliaris	bee	bee	FReD [Arnold et al. (2008)]
Alkanna strigosa	bee	bee	FReD [Arnold et al. (2008)]
Allium neapolitanum	bee	bee	FReD [Arnold et al. (2008)]
Allium nigrum	bee	bee	FReD [Arnold et al. (2008)]
Allium trifoliatum	bee	bee	FReD [Arnold et al. (2008)]
Aloe arborescens	bird	bird	Nicolson (2002)
Anagyris foetida	bee	bee	FReD [Arnold et al. (2008)]
Ananas bracteatus	bird	bird	Cestari (2009)
Anchusa officinalis	bee+insect	bee-butterfly	FReD [Arnold et al. (2008)]
Anchusa strigosa	bee	bee	FReD [Arnold et al. (2008)]

Anemone coronaria	insect	beetle	FReD [Arnold et al. (2008)]
Anemone nemorosa	bee	bee	FReD [Arnold et al. (2008)]
Anemone ranunculoides	bee	bee	FReD [Arnold et al. (2008)]
Anthemis maris-mortui	bee+insect	bee-beetle-fly	FReD [Arnold et al. (2008)]
Anthemis melampodina	bee+insect	bee-beetle-fly	FReD [Arnold et al. (2008)]
Anthemis pseudocotula	bee+insect	bee-beetle-fly	FReD [Arnold et al. (2008)]
Arabidopsis arenosa	bee+insect	bee-fly	FReD [Arnold et al. (2008)]
Arabidopsis thaliana	insect	fly	FReD [Arnold et al. (2008)]
Arbutus andrachne	bee	bee	FReD [Arnold et al. (2008)]
Arctostaphylos uva-ursi	bee	bee	FReD [Arnold et al. (2008)]
Arenaria serpyllifolia	insect	fly	FReD [Arnold et al. (2008)]
Asphodelus aestivus	bee	bee	FReD [Arnold et al. (2008)]
Asteriscus graveolens	bee+insect	bee-beetle-fly	FReD [Arnold et al. (2008)]
Astragalus amalecitanus	bee	bee	FReD [Arnold et al. (2008)]
Astragalus glycyphyllos	bee+insect	bee-butterfly	FReD [Arnold et al. (2008)]
Astragalus sanctus	bee	bee	FReD [Arnold et al. (2008)]
Asystasia gangetica	bee+insect	bee-butterfly	Freeman et al. (1991); Murali et al. (2013)
Bartsia alpina	bee	bee	FReD [Arnold et al. (2008)]
Bellevalia flexuosa	bee	bee	FReD [Arnold et al. (2008)]
Billbergia amoena	bird	bird	Capucho et al. (2007)
Billbergia pyramidalis	bird	bird	Sazima et al. (1995); pers. obs.*
Bistorta officinalis	bee	bee	FReD [Arnold et al. (2008)]
Buglossoides incrassata	bee	bee	FReD [Arnold et al. (2008)]
Calendula arvensis	bee+insect	bee-beetle-fly	FReD [Arnold et al. (2008)]
Calicotome villosa	bee	bee	FReD [Arnold et al. (2008)]
Calluna vulgaris	bee+insect	bee-beetle-fly	FReD [Arnold et al. (2008)]
Campanula latifolia	bee+insect	bee-fly	FReD [Arnold et al. (2008)]
Campanula rapunculoides	bee	bee	FReD [Arnold et al. (2008)]
Campanula trachelium	bee	bee	FReD [Arnold et al. (2008)]
Canistrum aurantiacum	bird	bird	Siqueira Filho and Machado (2001); pers. obs.*
Canistrum superbum	bird	bird	pers. obs.*
Canna indica	bird	bird	Glinos and Cocucci (2011)
Capsella bursa-pastoris	bee+insect	bee-fly	FReD [Arnold et al. (2008)]
Cardamine pratensis	bee+insect	bee-butterfly	FReD [Arnold et al. (2008)]
Centaurea aegyptiaca	bee	bee	FReD [Arnold et al. (2008)]
Centaurea ammocyanus	bee	bee	FReD [Arnold et al. (2008)]

Centaurea pallescens	bee	bee	FReD [Arnold et al. (2008)]
Cerastium holosteoides	bee+insect	bee-fly	FReD [Arnold et al. (2008)]
Cercis siliquastrum	bee	bee	FReD [Arnold et al. (2008)]
Chelidonium majus	bee+insect	bee-fly	FReD [Arnold et al. (2008)]
Cirsium oleraceum	bee	bee	FReD [Arnold et al. (2008)]
Cistus incanus	bee	bee	FReD [Arnold et al. (2008)]
Cistus salviifolius	bee	bee	FReD [Arnold et al. (2008)]
Cleistocactus straussii	bird	bird	Rose and Barthlott (1994)
Clinopodium vulgare	bee+insect	bee-butterfly	FReD [Arnold et al. (2008)]
Colutea istria	bee	bee	FReD [Arnold et al. (2008)]
Corylus avellana	bee	bee	FReD [Arnold et al. (2008)]
Crataegus azarolus	bee+insect	bee-beetle-fly	FReD [Arnold et al. (2008)]
Crepis aspera	bee+insect	bee-beetle-fly	FReD [Arnold et al. (2008)]
Crepis hierosolymitana	bee+insect	bee-beetle-fly	FReD [Arnold et al. (2008)]
Crepis palaestina	bee+insect	bee-beetle-fly	FReD [Arnold et al. (2008)]
Crepis sancta	bee+insect	bee-beetle-fly	FReD [Arnold et al. (2008)]
Crupina crupinastrum	bee	bee	FReD [Arnold et al. (2008)]
Cyclamen persicum	bee	bee	FReD [Arnold et al. (2008)]
Cynoglossum officinale	bee+insect	bee-butterfly	FReD [Arnold et al. (2008)]
Cyrtopodium flavum	bee	bee	Pemberton and Liu (2011)
Dahlstedtia pinnata	bird	bird	Sazima et al. (1995)
Dianthus carthusianorum	bee+insect	bee-butterfly	FReD [Arnold et al. (2008)]
Diplotaxis harra	bee	bee	FReD [Arnold et al. (2008)]
Dracaena reflexa	bee	bee	Rasoloarijao et al. (2019)
Echium angustifolium	bee	bee	FReD [Arnold et al. (2008)]
Echium rauwolfii	bee	bee	FReD [Arnold et al. (2008)]
Emilia sonchifolia	insect	butterfly	FReD [Arnold et al. (2008)]
Encyclia cordigera	bee	bee	Cingel (2001); Sugiura (2013)
Epilobium angustifolium	bee+insect	bee-butterfly-fly	FReD [Arnold et al. (2008)]
Epilobium parviflorum	bee	bee	FReD [Arnold et al. (2008)]
Erigeron canadensis	bee	bee	FReD [Arnold et al. (2008)]
Erucaria pinnata	bee	bee	FReD [Arnold et al. (2008)]
Erysimum cheiranthoides	bee+insect	bee-fly	FReD [Arnold et al. (2008)]
Erythrina falcata	bird	bird	Galetti (1993); Bruneau (1997); Etcheverry and Alemán (2005)
Erythrina fusca	bird	bird	Morton (1979)
Erythrina speciosa	bird	bird	Sazima et al. (1995)

Eugenia brasiliensis	bee	bee	Wilms et al. (1996)
Eugenia uniflora	bee	bee	Silva and Pinheiro (2009)
Euphorbia hierosolymitana	insect	fly	FReD [Arnold et al. (2008)]
Ficaria verna	insect	fly	FReD [Arnold et al. (2008)]
Filipendula ulmaria	bee	bee	FReD [Arnold et al. (2008)]
Fragaria viridis	bee+insect	bee-butterfly	FReD [Arnold et al. (2008)]
Galeopsis bifida	bee	bee	FReD [Arnold et al. (2008)]
Galeopsis pubescens	bee	bee	FReD [Arnold et al. (2008)]
Galeopsis tetrahit	bee	bee	FReD [Arnold et al. (2008)]
Galium verum	bee	bee	FReD [Arnold et al. (2008)]
Gasteria pulchra	bird	bird	Jaarsveld (2012)
Gazania heterochaeta	insect	beetle	FReD [Arnold et al. (2008)]
Geranium sylvaticum	insect	fly	FReD [Arnold et al. (2008)]
Geum rivale	bee	bee	FReD [Arnold et al. (2008)]
Glechoma hederacea	bee	bee	FReD [Arnold et al. (2008)]
Gliricidia sepium	bee	bee	Kiill and Drumond (2001)
Gmelina arborea	bee	bee	Bolstad and Bawa (1982)
Gmelina asiatica	bee	bee	Raju and Reddi (2000)
Gomesa flexuosa	bee	bee	Gomiz et al. (2013)
Gustavia augusta	bee	bee	Kimmel et al. (2010)
Gymnocarpos decandrus	insect	beetle-fly	FReD [Arnold et al. (2008)]
Gypsophila arabica	insect	beetle-fly	FReD [Arnold et al. (2008)]
Hedypnois rhagadioloides	bee+insect	bee-beetle-fly	FReD [Arnold et al. (2008)]
Helianthemum ventosum	bee	bee	FReD [Arnold et al. (2008)]
Helianthemum vesicarium	bee	bee	FReD [Arnold et al. (2008)]
Heliconia metallica	bird	bird	Schleuning et al. (2011)
Heliconia psittacorum	bird	bird	Feinsinger et al. (1982); Feinsinger (1983)
Helicteres brevispira	bird	bird	Franceschinelli and Bawa (2000); Franceschinelli (2005); Wolowski et al. (2013)
Hepatica nobilis	bee	bee	FReD [Arnold et al. (2008)]
Hesperis pendula	bee	bee	FReD [Arnold et al. (2008)]
Heterotaxis sessilis	bee	bee	Molgo et al. (2007)
Hibiscus rosa-sinensis	bird	bird	FReD [Arnold et al. (2008)]
Hibiscus tiliaceus	bee	bee	McMullen (1989); Azmi and Mohamed (2012)
Hieracium laevigatum	bee+insect	bee-fly	FReD [Arnold et al. (2008)]
Hieracium sabaudum	bee+insect	bee-fly	FReD [Arnold et al. (2008)]
Holosteum umbellatum	insect	beetle-fly	FReD [Arnold et al. (2008)]

Hylotelephium maximum	bee	bee	FReD [Arnold et al. (2008)]
Hyoscyamus aureus	bee	bee	FReD [Arnold et al. (2008)]
Hypoxis decumbens	bee+insect	bee-fly	Viana and Alves-dos Santos (2002)
Ipomoea squamosa	bee+insect	bee-beetle	FReD [Arnold et al. (2008)]
Isatis lusitanica	bee+insect	bee-beetle-fly	FReD [Arnold et al. (2008)]
Jacobaea vulgaris	bee+insect	bee-fly	FReD [Arnold et al. (2008)]
Justicia brandegeeana	bird	bird	FReD [Arnold et al. (2008)]
Justicia gendarussa	bee	bee	Layek et al. (2015); Singh et al. (2016)
Knautia arvensis	bee+insect	bee-butterfly	FReD [Arnold et al. (2008)]
Knautia dipsacifolia	insect	butterfly	FReD [Arnold et al. (2008)]
Lamium album	bee	bee	FReD [Arnold et al. (2008)]
Lamium galeobdolon	bee	bee	FReD [Arnold et al. (2008)]
Lamium garganicum	bee	bee	FReD [Arnold et al. (2008)]
Lapsana communis	bee+insect	bee-fly	FReD [Arnold et al. (2008)]
Lathyrus blepharicarpus	bee	bee	FReD [Arnold et al. (2008)]
Lathyrus gorgoni	bee	bee	FReD [Arnold et al. (2008)]
Lathyrus pratensis	bee	bee	FReD [Arnold et al. (2008)]
Lathyrus vernus	bee	bee	FReD [Arnold et al. (2008)]
Launaea angustifolia	bee+insect	bee-beetle-fly	FReD [Arnold et al. (2008)]
Launaea mucronata	bee+insect	bee-beetle-fly	FReD [Arnold et al. (2008)]
Launaea nudicaulis	bee+insect	bee-beetle-fly	FReD [Arnold et al. (2008)]
Lebeckia halenbergensis	bee	bee	FReD [Arnold et al. (2008)]
Leontodon laciniatus	bee+insect	bee-beetle-fly	FReD [Arnold et al. (2008)]
Leontodon tuberosus	bee+insect	bee-beetle-fly	FReD [Arnold et al. (2008)]
Leopoldia comosa	bee	bee	FReD [Arnold et al. (2008)]
Leopoldia longipes	bee	bee	FReD [Arnold et al. (2008)]
Lepidium draba	bee+insect	bee-beetle-fly	FReD [Arnold et al. (2008)]
Liatris spicata	insect	fly	FReD [Arnold et al. (2008)]
Limodorum abortivum	bee	bee	FReD [Arnold et al. (2008)]
Linaria vulgaris	bee	bee	FReD [Arnold et al. (2008)]
Linum pubescens	insect	fly	FReD [Arnold et al. (2008)]
Lobelia anceps	bee	bee	Soper and Beggs (2013)
Lotus longesiliquosus	bee	bee	FReD [Arnold et al. (2008)]
Lycium shawii	bee	bee	FReD [Arnold et al. (2008)]
Maranta leuconeura	bee	bee	Azevedo et al. (2014)
Matricaria aurea	bee+insect	bee-beetle-fly	FReD [Arnold et al. (2008)]

Melampyrum pratense	bee	bee	FReD [Arnold et al. (2008)]
Melampyrum sylvaticum	bee	bee	FReD [Arnold et al. (2008)]
Mentha aquatica	bee+insect	bee-beetle-butterfly-fly	FReD [Arnold et al. (2008)]
Mesembryanthemum cryptanthum	insect	beetle-fly	FReD [Arnold et al. (2008)]
Mesembryanthemum nodiflorum	insect	beetle-fly	FReD [Arnold et al. (2008)]
Moehringia trinervia	bee	bee	FReD [Arnold et al. (2008)]
Moricandia nitens	bee	bee	FReD [Arnold et al. (2008)]
Myosotis alpestris	insect	fly	FReD [Arnold et al. (2008)]
Myosotis decumbens	insect	fly	FReD [Arnold et al. (2008)]
Myosotis stricta	bee+insect	bee-fly	FReD [Arnold et al. (2008)]
Myosotis vestergrenii	insect	fly	FReD [Arnold et al. (2008)]
Nanorrhinum scoparium	bee	bee	FReD [Arnold et al. (2008)]
Neoregelia cruenta	bird	bird	Fonseca et al. (2015); pers. obs.*
Onobrychis crista-galli	bee	bee	FReD [Arnold et al. (2008)]
Ononis natrix	bee	bee	FReD [Arnold et al. (2008)]
Orchis italica	bee	bee	FReD [Arnold et al. (2008)]
Origanum vulgare	insect	butterfly	FReD [Arnold et al. (2008)]
Oxytropis jacquinii	bee	bee	FReD [Arnold et al. (2008)]
Oxytropis neglecta	bee	bee	FReD [Arnold et al. (2008)]
Papaver hybridum	bee+insect	bee-beetle-fly	FReD [Arnold et al. (2008)]
Papaver rhoeas	bee	bee	FReD [Arnold et al. (2008)]
Papaver umbonatum	bee+insect	bee-beetle-fly	FReD [Arnold et al. (2008)]
Parnassia palustris	insect	butterfly-fly	FReD [Arnold et al. (2008)]
Pedicularis comosa	bee	bee	FReD [Arnold et al. (2008)]
Pedicularis recutita	bee	bee	FReD [Arnold et al. (2008)]
Phlomoides laciniata	bee	bee	FReD [Arnold et al. (2008)]
Phyllodoce caerulea	bee	bee	FReD [Arnold et al. (2008)]
Phyteuma betonicifolium	bee	bee	FReD [Arnold et al. (2008)]
Phyteuma hedraianthifolium	bee	bee	FReD [Arnold et al. (2008)]
Phyteuma hemisphaericum	bee	bee	FReD [Arnold et al. (2008)]
Phyteuma nigrum	bee	bee	FReD [Arnold et al. (2008)]
Picris longirostris	bee+insect	bee-beetle-fly	FReD [Arnold et al. (2008)]
Pilosella officinarum	insect	fly	FReD [Arnold et al. (2008)]
Pinguicula alpina	bee	bee	FReD [Arnold et al. (2008)]
Platanthera clavellata	bee	bee	FReD [Arnold et al. (2008)]
Potentilla heptaphylla	bee+insect	bee-fly	FReD [Arnold et al. (2008)]

Potentilla reptans	bee+insect	bee-fly	FReD [Arnold et al. (2008)]
Primula elatior	bee	bee	FReD [Arnold et al. (2008)]
Prunus padus	bee+insect	bee-butterfly-fly	FReD [Arnold et al. (2008)]
Pseudolaelia corcovadensis	bee	bee	Borba and Braga (2003)
Pulicaria incisa	bee+insect	bee-beetle-fly	FReD [Arnold et al. (2008)]
Pulmonaria mollis	bee	bee	FReD [Arnold et al. (2008)]
Pulmonaria obscura	bee+insect	bee-butterfly	FReD [Arnold et al. (2008)]
Quesnelia arvensis	bird	bird	Sazima et al. (1995)
Quesnelia liboniana	bird	bird	Martinelli (1995)
Ranunculus acris	bee+insect	bee-fly	FReD [Arnold et al. (2008)]
Ranunculus asiaticus	insect	beetle	FReD [Arnold et al. (2008)]
Ranunculus marginatus	bee+insect	bee-beetle-fly	FReD [Arnold et al. (2008)]
Ranunculus millefolius	bee+insect	bee-beetle-fly	FReD [Arnold et al. (2008)]
Retama raetam	bee	bee	FReD [Arnold et al. (2008)]
Rhagadiolus stellatus	bee+insect	bee-beetle-fly	FReD [Arnold et al. (2008)]
Rivina humilis	bee	bee	Kato et al. (1999); Cate (2007)
Ruta chalepensis	insect	fly	FReD [Arnold et al. (2008)]
Salvia dominica	bee	bee	FReD [Arnold et al. (2008)]
Salvia fruticosa	bee	bee	FReD [Arnold et al. (2008)]
Salvia hierosolymitana	bee	bee	FReD [Arnold et al. (2008)]
Salvia lanigera	bee	bee	FReD [Arnold et al. (2008)]
Sanchezia oblonga	bird	bird	FReD [Arnold et al. (2008)]
Sanchezia speciosa	bird	bird	Tripp and McDade (2013); Costa et al. (2017)
Satureja thymbra	bee	bee	FReD [Arnold et al. (2008)]
Scandix pecten-veneris	bee+insect	bee-fly	FReD [Arnold et al. (2008)]
Scilla hyacinthoides	bee	bee	FReD [Arnold et al. (2008)]
Scorzonera papposa	bee+insect	bee-beetle-fly	FReD [Arnold et al. (2008)]
Scorzoneroides autumnalis	bee	bee	FReD [Arnold et al. (2008)]
Scrophularia xanthoglossa	bee	bee	FReD [Arnold et al. (2008)]
Securigera varia	bee	bee	FReD [Arnold et al. (2008)]
Senecio glaucus	bee+insect	bee-beetle-fly	FReD [Arnold et al. (2008)]
Senecio vernalis	bee+insect	bee-beetle-fly	FReD [Arnold et al. (2008)]
Silene acaulis	bee+insect	bee-fly	FReD [Arnold et al. (2008)]
Silene aegyptiaca	bee	bee	FReD [Arnold et al. (2008)]
Silene flos-cuculi	bee+insect	bee-butterfly	FReD [Arnold et al. (2008)]
Silene nutans	bee+insect	bee-butterfly	FReD [Arnold et al. (2008)]

Sinapis arvensis	bee+insect	bee-beetle-butterfly-fly	FReD [Arnold et al. (2008)]
Sinapis incana	bee+insect	bee-beetle-fly	FReD [Arnold et al. (2008)]
Solidago canadensis	bee+insect	bee-fly	FReD [Arnold et al. (2008)]
Sonchus oleraceus	bee+insect	bee-beetle-fly	FReD [Arnold et al. (2008)]
Stachys recta	bee+insect	bee-butterfly	FReD [Arnold et al. (2008)]
Stachys sylvatica	bee+insect	bee-butterfly-fly	FReD [Arnold et al. (2008)]
Stellaria holostea	bee+insect	bee-beetle-butterfly-fly	FReD [Arnold et al. (2008)]
Stellaria palustris	bee+insect	bee-butterfly-fly	FReD [Arnold et al. (2008)]
Swartzia simplex	bee	bee	Pinheiro et al. (2018)
Symphoricarpos albus	bee+insect	bee-butterfly-fly	FReD [Arnold et al. (2008)]
Symphytum brachycalyx	bee	bee	FReD [Arnold et al. (2008)]
Tamarix nilotica	bee+insect	bee-beetle-fly	FReD [Arnold et al. (2008)]
Taraxacum officinale	bee+insect	bee-fly	FReD [Arnold et al. (2008)]
Thymus serpyllum	insect	butterfly	FReD [Arnold et al. (2008)]
Tillandsia polystachia	bird	bird	Coser et al. (2008)
Tridax procumbens	bee+insect	bee-butterfly	Varalakshmi and Raju (2013); Bhalchandra et al. (2014)
Trifolium clypeatum	bee	bee	FReD [Arnold et al. (2008)]
Trifolium repens	bee	bee	FReD [Arnold et al. (2008)]
Trifolium resupinatum	bee	bee	FReD [Arnold et al. (2008)]
Trifolium stellatum	bee	bee	FReD [Arnold et al. (2008)]
Trigonella caelesyriaca	bee	bee	FReD [Arnold et al. (2008)]
Trigonella kotschyi	bee	bee	FReD [Arnold et al. (2008)]
Tripleurospermum auriculatum	bee+insect	bee-beetle-fly	FReD [Arnold et al. (2008)]
Tussilago farfara	bee+insect	bee-beetle-fly	FReD [Arnold et al. (2008)]
Urospermum picroides	bee+insect	bee-beetle-fly	FReD [Arnold et al. (2008)]
Ursinia cakilefolia	bee	bee	FReD [Arnold et al. (2008)]
Vaccinium vitis-idaea	bee	bee	FReD [Arnold et al. (2008)]
Verbascum densiflorum	bee+insect	bee-butterfly-fly	FReD [Arnold et al. (2008)]
Verbascum lychnitis	bee+insect	bee-butterfly	FReD [Arnold et al. (2008)]
Veronica arvensis	bee+insect	bee-fly	FReD [Arnold et al. (2008)]
Veronica chamaedrys	bee+insect	bee-fly	FReD [Arnold et al. (2008)]
Veronica prostrata	bee+insect	bee-butterfly-fly	FReD [Arnold et al. (2008)]
Veronica spicata	bee+insect	bee-fly	FReD [Arnold et al. (2008)]
Viburnum opulus	bee+insect	bee-butterfly-fly	FReD [Arnold et al. (2008)]
Vicia hybrida	bee	bee	FReD [Arnold et al. (2008)]
Vicia sativa	bee	bee	FReD [Arnold et al. (2008)]

Vigna unguiculata	bee+insect	bee-butterfly-fly	Fohouo et al. (2009); Ige et al. (2011)
Vincetoxicum hirundinaria	bee+insect	bee-butterfly-fly	FReD [Arnold et al. (2008)]
Vriesea neoglutinosa	bird	bird	Fonseca et al. (2015); Magalhães et al. (2018)
Zilla spinosa	bee	bee	FReD [Arnold et al. (2008)]

Table S3: *personal observations made at the Botanical Garden of Rio de Janeiro for species native to the Atlantic Forest in a parallel research project

Table S4: Achromatic (**ACB**) and chromatic (**CCB**) contrasts against the background, spectral purity (**SP**, according to the visual systems of *Apis mellifera* L. and *Bombus terrestris* L.) and flower depth in millimeters for the whole dataset used for analysis. **Dataset** indicates whether each species was used for the analyses involving pollination systems (1) and flower depth (2).

**Species**	**Dataset**	**Depth**	ACB_{Apis}	ACB_{Bombus}	CCB_{Apis}	CCB_{Bombus}	SP_{Apis}	SP_{Bombus}
Aaronsohnia factorovskyi	1	0.00	0.292	0.276	0.195	0.191	0.408	0.510
Acanthus montanus	1, 2	34.20	0.395	0.387	0.127	0.306	0.338	0.841
Achillea santolina	1	0.00	0.292	0.276	0.195	0.191	0.408	0.510
Aconitum septentrionale	1, 2	12.00	0.198	0.195	0.098	0.131	0.262	0.361
Aechmea aquilega	1, 2	34.03	0.190	0.182	0.093	0.129	0.247	0.355
Aechmea bambusoides	2	27.35	0.102	0.100	0.056	0.076	0.148	0.209
Aechmea blanchetiana	2	28.10	0.129	0.122	0.048	0.049	0.127	0.135
Aechmea bromeliifolia	1, 2	16.69	0.087	0.082	0.037	0.056	0.099	0.154
Aechmea eurycorymbus	1, 2	26.17	0.221	0.203	0.129	0.123	0.320	0.330
Aechmea floribunda	2	25.91	0.381	0.374	0.217	0.288	0.552	0.794
Aechmea nudicaulis	2	15.39	0.097	0.092	0.055	0.059	0.145	0.162
Aechmea pectinata	1, 2	22.52	0.339	0.331	0.111	0.205	0.290	0.563
Aechmea purpureorosea	1, 2	20.72	0.178	0.176	0.114	0.157	0.305	0.433
Aechmea sp.	1, 2	23.44	0.282	0.265	0.151	0.141	0.363	0.378
Aechmea tomentosa	1, 2	46.22	0.151	0.142	0.056	0.089	0.149	0.244
Aechmea vallerandii	2	31.56	0.064	0.062	0.019	0.045	0.052	0.125
Agrimonia eupatoria	1	0.00	0.291	0.273	0.173	0.215	0.333	0.517
Ainsworthia trachycarpa	1	0.00	0.410	0.401	0.230	0.289	0.571	0.795
Ajuga chamaepitys	1, 2	3.00	0.316	0.300	0.213	0.209	0.440	0.560

Ajuga genevensis	1, 2	8.00	0.241	0.236	0.064	0.152	0.172	0.419
Ajuga pyramidalis	1, 2	8.00	0.314	0.308	0.121	0.211	0.323	0.581
Ajuga reptans	1, 2	8.00	0.195	0.195	0.149	0.262	0.386	0.670
Alcantarea extensa	2	41.36	0.091	0.085	0.061	0.092	0.162	0.211
Alcea acaulis	1, 2	2.00	0.326	0.320	0.168	0.233	0.436	0.640
Alcea dissecta	1, 2	3.00	0.142	0.142	0.081	0.155	0.211	0.397
Alectis ciliaris	1, 2	8.00	0.222	0.219	0.114	0.213	0.305	0.546
Alkanna strigosa	1, 2	7.00	0.164	0.161	0.090	0.159	0.235	0.406
Allamanda cathartica	2	62.72	0.338	0.322	0.205	0.202	0.378	0.483
Allium neapolitanum	1	0.00	0.405	0.398	0.100	0.282	0.267	0.776
Allium nigrum	1	0.00	0.400	0.392	0.207	0.289	0.525	0.796
Allium trifoliatum	1	0.00	0.407	0.400	0.157	0.317	0.416	0.872
Aloe arborescens	1, 2	30.43	0.399	0.390	0.184	0.264	0.456	0.726
Aloe ciliaris	2	18.21	0.224	0.214	0.080	0.084	0.211	0.226
Aloe glauca	2	25.21	0.186	0.174	0.086	0.096	0.224	0.264
Aloe sp.	2	27.78	0.238	0.225	0.125	0.153	0.317	0.422
Alpinia zerumbet	2	46.30	0.303	0.299	0.113	0.295	0.301	0.756
Amherstia nobilis	2	31.80	0.183	0.172	0.077	0.071	0.202	0.191
Anagyris foetida	1, 2	10.00	0.298	0.283	0.159	0.164	0.382	0.439
Ananas bracteatus	1, 2	20.96	0.064	0.062	0.010	0.019	0.026	0.053
Anchusa officinalis	1, 2	6.00	0.113	0.111	0.090	0.127	0.236	0.326
Anchusa strigosa	1, 2	9.00	0.087	0.088	0.108	0.154	0.277	0.394
Andira legalis	2	22.30	0.047	0.049	0.099	0.087	0.253	0.223
Anemone coronaria	1	0.00	0.060	0.059	0.015	0.025	0.040	0.070
Anemone nemorosa	1	0.00	0.406	0.398	0.217	0.300	0.550	0.826
Anemone ranunculoides	1	0.00	0.317	0.300	0.199	0.193	0.367	0.463
Anthemis maris-mortui	1, 2	4.50	0.292	0.276	0.192	0.187	0.402	0.500
Anthemis melampodina	1	0.00	0.279	0.262	0.204	0.194	0.423	0.518
Anthemis pseudocotula	1	0.00	0.277	0.258	0.203	0.190	0.419	0.509
Arabidopsis arenosa	1, 2	2.00	0.429	0.421	0.233	0.335	0.591	0.921
Arabidopsis thaliana	1	0.00	0.425	0.416	0.225	0.306	0.565	0.841
Arbutus andrachne	1	0.00	0.377	0.368	0.216	0.255	0.527	0.702
Arctostaphylos uva-ursi	1, 2	6.00	0.335	0.328	0.147	0.233	0.390	0.640
Arenaria serpyllifolia	1	0.00	0.390	0.381	0.172	0.261	0.436	0.717
Asphodelus aestivus	1, 2	2.00	0.401	0.393	0.084	0.286	0.224	0.788
Asteriscus graveolens	1	0.00	0.272	0.254	0.186	0.176	0.389	0.472

Astragalus amalecitanus	1, 2	13.00	0.344	0.337	0.157	0.230	0.412	0.633
Astragalus glycyphyllos	1, 2	7.00	0.403	0.393	0.149	0.153	0.368	0.410
Astragalus sanctus	1, 2	15.00	0.344	0.337	0.157	0.230	0.412	0.633
Asystasia gangetica	1, 2	23.57	0.399	0.391	0.200	0.295	0.509	0.812
Barleria cristata	2	47.26	0.269	0.268	0.146	0.257	0.391	0.657
Barleria repens	2	48.87	0.071	0.069	0.058	0.026	0.156	0.072
Bartsia alpina	1, 2	8.00	0.131	0.129	0.042	0.069	0.111	0.190
Bellevalia flexuosa	1, 2	8.00	0.383	0.374	0.203	0.230	0.503	0.633
Billbergia amoena	1, 2	35.54	0.327	0.318	0.179	0.215	0.437	0.576
Billbergia pyramidalis	1, 2	35.79	0.118	0.109	0.056	0.071	0.150	0.197
Bistorta officinalis	1, 2	2.00	0.323	0.317	0.162	0.210	0.426	0.579
Bougainvillea spectabilis	2	17.30	0.088	0.092	0.186	0.217	0.363	0.494
Brownea ariza	2	55.91	0.028	0.030	0.020	0.023	0.054	0.062
Brunfelsia uniflora	2	22.88	0.171	0.169	0.116	0.188	0.309	0.482
Bryophyllum fedtschenkoi	2	12.13	0.086	0.082	0.058	0.057	0.154	0.154
Bryophyllum laetivirens	2	23.62	0.111	0.110	0.038	0.032	0.101	0.089
Buglossoides incrassata	1, 2	2.00	0.385	0.377	0.217	0.253	0.538	0.697
Calea phyllolepis	2	5.10	0.295	0.276	0.222	0.213	0.455	0.571
Calendula arvensis	1	0.00	0.259	0.239	0.176	0.157	0.416	0.367
Calicotome villosa	1, 2	6.00	0.318	0.302	0.206	0.196	0.376	0.458
Calliandra harrisii	2	26.68	0.132	0.129	0.124	0.122	0.237	0.253
Calluna vulgaris	1	0.00	0.235	0.232	0.099	0.164	0.263	0.451
Camellia japonica	2	1.00	0.104	0.104	0.192	0.144	0.358	0.309
Camellia sinensis	2	13.32	0.405	0.396	0.209	0.297	0.524	0.816
Camoensia scandens	2	87.11	0.387	0.380	0.144	0.331	0.385	0.846
Campanula latifolia	1	0.00	0.194	0.193	0.131	0.181	0.331	0.463
Campanula rapunculoides	1	0.00	0.277	0.274	0.087	0.208	0.231	0.532
Campanula trachelium	1	0.00	0.199	0.199	0.148	0.236	0.372	0.603
Canistrum aurantiacum	1, 2	27.51	0.035	0.036	0.012	0.017	0.033	0.046
Canistrum fragrans	2	18.28	0.138	0.134	0.068	0.089	0.181	0.245
Canistrum superbum	1, 2	40.63	0.133	0.124	0.010	0.041	0.026	0.112
Canna indica	1, 2	48.75	0.221	0.203	0.058	0.093	0.154	0.257
Capsella bursa-pastoris	1	0.00	0.403	0.395	0.149	0.269	0.395	0.741
Carapa guianensis	2	3.67	0.339	0.329	0.166	0.221	0.412	0.609
Cardamine pratensis	1, 2	4.00	0.366	0.360	0.188	0.266	0.497	0.732
Cascabela thevetia	2	61.39	0.137	0.131	0.044	0.047	0.118	0.131

Centaurea aegyptiaca	1, 2	5.00	0.345	0.337	0.118	0.236	0.310	0.651
Centaurea ammocyanus	1, 2	3.00	0.252	0.247	0.100	0.199	0.268	0.510
Centaurea pallescens	1, 2	3.00	0.361	0.351	0.216	0.240	0.519	0.642
Cerastium holosteoides	1	0.00	0.431	0.423	0.197	0.324	0.512	0.891
Cercis siliquastrum	1, 2	7.00	0.229	0.227	0.145	0.221	0.388	0.565
Chelidonium majus	1	0.00	0.301	0.282	0.190	0.267	0.366	0.632
Cirsium oleraceum	1, 2	30.00	0.369	0.360	0.195	0.222	0.477	0.594
Cistus incanus	1, 2	2.00	0.312	0.296	0.219	0.216	0.453	0.579
Cistus salviifolius	1, 2	2.00	0.300	0.285	0.204	0.204	0.427	0.546
Cleistocactus straussii	1, 2	79.60	0.046	0.048	0.070	0.105	0.182	0.270
Clerodendrum quadriloculare	2	89.37	0.057	0.056	0.029	0.036	0.077	0.099
Clerodendrum thomsoniae	2	18.11	0.361	0.354	0.211	0.251	0.516	0.692
Clinopodium vulgare	1, 2	6.00	0.412	0.403	0.229	0.285	0.576	0.785
Colutea istria	1, 2	8.00	0.286	0.269	0.168	0.174	0.318	0.421
Combretum indicum	2	36.33	0.124	0.121	0.066	0.092	0.176	0.253
Combretum paniculatum	2	6.59	0.116	0.109	0.036	0.038	0.097	0.102
Combretum rotundifolium	2	10.15	0.179	0.168	0.105	0.109	0.264	0.292
Congea tomentosa	2	5.16	0.314	0.307	0.145	0.255	0.386	0.701
Corylus avellana	1	0.00	0.274	0.263	0.153	0.172	0.380	0.460
Crataegus azarolus	1, 2	1.00	0.402	0.394	0.220	0.294	0.553	0.808
Crepis aspera	1, 2	2.00	0.300	0.283	0.214	0.199	0.437	0.533
Crepis hierosolymitana	1, 2	2.00	0.325	0.311	0.211	0.201	0.383	0.470
Crepis palaestina	1, 2	2.00	0.337	0.322	0.212	0.208	0.388	0.500
Crepis sancta	1, 2	2.00	0.327	0.312	0.203	0.208	0.425	0.556
Crinum asiaticum	2	78.75	0.415	0.407	0.198	0.340	0.520	0.937
Crinum latifolium	2	86.45	0.403	0.396	0.126	0.330	0.337	0.845
Crupina crupinastrum	1	0.00	0.272	0.267	0.137	0.200	0.364	0.549
Cuphea racemosa	2	1.00	0.120	0.119	0.057	0.089	0.153	0.245
Cyclamen persicum	1, 2	4.00	0.324	0.319	0.178	0.258	0.470	0.709
Cynoglossum officinale	1, 2	2.00	0.037	0.037	0.102	0.097	0.263	0.208
Cyrtopodium flavum	1, 2	19.64	0.331	0.315	0.171	0.190	0.328	0.456
Dahlstedtia pinnata	1, 2	37.75	0.174	0.169	0.047	0.116	0.124	0.319
Deuterocohnia meziana	2	21.10	0.128	0.122	0.068	0.079	0.181	0.217
Dianthus carthusianorum	1	0.00	0.103	0.102	0.088	0.110	0.233	0.303
Dietes bicolor	2	8.64	0.344	0.336	0.195	0.242	0.489	0.665
Diplotaxis harra	1, 2	5.00	0.367	0.354	0.239	0.238	0.488	0.637

Dracaena reflexa	1, 2	22.16	0.176	0.170	0.080	0.109	0.210	0.301
Duranta erecta	2	6.74	0.245	0.244	0.149	0.246	0.400	0.629
Dyckia brevifolia	2	9.07	0.285	0.268	0.190	0.191	0.396	0.510
Dyckia encholirioides	2	12.38	0.254	0.235	0.163	0.163	0.391	0.435
Echeveria sp.	2	11.20	0.368	0.355	0.198	0.233	0.413	0.623
Echium angustifolium	1, 2	3.00	0.020	0.019	0.036	0.033	0.095	0.091
Echium rauwolfii	1, 2	3.00	0.201	0.195	0.065	0.092	0.169	0.234
Emilia sonchifolia	1	0.00	0.101	0.100	0.038	0.054	0.101	0.150
Encyclia cordigera	1, 2	16.07	0.069	0.069	0.128	0.150	0.244	0.310
Epilobium angustifolium	1, 2	18.00	0.130	0.129	0.096	0.134	0.255	0.344
Epilobium parviflorum	1	0.00	0.292	0.288	0.159	0.216	0.426	0.552
Episcia cupreata	2	26.07	0.058	0.056	0.027	0.028	0.072	0.078
Erigeron canadensis	1	0.00	0.431	0.423	0.177	0.312	0.465	0.859
Erucaria pinnata	1, 2	3.00	0.415	0.407	0.211	0.310	0.537	0.852
Erysimum cheiranthoides	1	0.00	0.338	0.322	0.186	0.177	0.340	0.413
Erythrina falcata	1, 2	41.88	0.111	0.103	0.037	0.049	0.099	0.134
Erythrina fusca	1, 2	22.31	0.310	0.300	0.119	0.169	0.298	0.453
Erythrina speciosa	1, 2	43.20	0.076	0.073	0.031	0.029	0.082	0.080
Etlingera elatior	2	19.89	0.146	0.142	0.025	0.082	0.067	0.227
Eugenia brasiliensis	1	0.00	0.372	0.363	0.202	0.262	0.501	0.721
Eugenia uniflora	1, 2	3.85	0.231	0.220	0.153	0.158	0.374	0.424
Euphorbia hierosolymitana	1	0.00	0.286	0.268	0.198	0.192	0.414	0.515
Ficaria verna	1	0.00	0.348	0.334	0.207	0.264	0.397	0.634
Filipendula ulmaria	1	0.00	0.427	0.419	0.217	0.253	0.538	0.696
Fragaria viridis	1	0.00	0.400	0.391	0.222	0.276	0.550	0.758
Galeopsis bifida	1, 2	7.00	0.120	0.119	0.091	0.120	0.242	0.307
Galeopsis pubescens	1	0.00	0.160	0.159	0.082	0.125	0.219	0.320
Galeopsis tetrahit	1, 2	6.00	0.405	0.396	0.194	0.257	0.486	0.707
Galium verum	1	0.00	0.304	0.286	0.204	0.198	0.425	0.530
Gasteria pulchra	1, 2	18.79	0.327	0.317	0.172	0.228	0.432	0.626
Gazania heterochaeta	1	0.00	0.170	0.159	0.092	0.100	0.242	0.268
Geranium sylvaticum	1	0.00	0.093	0.096	0.168	0.145	0.322	0.312
Geum rivale	1	0.00	0.301	0.293	0.027	0.024	0.071	0.066
Glechoma hederacea	1, 2	10.00	0.210	0.207	0.119	0.185	0.318	0.510
Gliricidia sepium	1, 2	18.20	0.400	0.390	0.229	0.270	0.541	0.722
Gmelina arborea	1, 2	14.48	0.082	0.079	0.039	0.049	0.104	0.134

Gmelina asiatica	1, 2	25.75	0.322	0.308	0.193	0.212	0.403	0.567
Gomesa flexuosa	1	0.00	0.352	0.338	0.197	0.214	0.372	0.517
Guarea guidonia	2	6.57	0.379	0.369	0.159	0.265	0.400	0.728
Guazuma ulmifolia	2	5.70	0.180	0.162	0.135	0.124	0.329	0.331
Gustavia augusta	1, 2	17.72	0.246	0.241	0.136	0.189	0.361	0.519
Gymnocarpos decandrus	1	0.00	0.204	0.196	0.098	0.110	0.258	0.303
Gypsophila arabica	1	0.00	0.366	0.359	0.022	0.114	0.059	0.315
Habranthus robustus	2	51.02	0.382	0.374	0.201	0.285	0.511	0.784
Handroanthus heptaphyllus	2	50.94	0.159	0.158	0.079	0.150	0.212	0.414
Haworthia attenuata	2	12.85	0.317	0.307	0.114	0.200	0.295	0.550
Hedychium coccineum	2	52.51	0.175	0.168	0.103	0.112	0.261	0.300
Hedypnois rhagadioloides	1, 2	2.00	0.292	0.276	0.201	0.190	0.415	0.509
Helianthemum ventosum	1	0.00	0.334	0.321	0.192	0.217	0.362	0.524
Helianthemum vesicarium	1	0.00	0.260	0.257	0.140	0.229	0.374	0.586
Heliconia metallica	1, 2	53.32	0.105	0.102	0.013	0.037	0.036	0.101
Heliconia pendula	2	48.15	0.103	0.098	0.043	0.060	0.115	0.164
Heliconia psittacorum	1, 2	32.61	0.148	0.137	0.081	0.088	0.212	0.236
Helicteres brevispira	1, 2	15.00	0.075	0.072	0.039	0.046	0.105	0.126
Hepatica nobilis	1	0.00	0.195	0.194	0.125	0.222	0.329	0.567
Hesperis pendula	1, 2	9.00	0.236	0.226	0.144	0.143	0.353	0.383
Heterotaxis sessilis	1, 2	14.53	0.252	0.231	0.192	0.180	0.401	0.483
Hibiscus rosa-sinensis	1	0.00	0.082	0.079	0.029	0.029	0.077	0.080
Hibiscus schizopetalus	2	14.97	0.044	0.045	0.007	0.018	0.019	0.050
Hibiscus tiliaceus	1, 2	2.00	0.312	0.305	0.200	0.214	0.471	0.572
Hieracium laevigatum	1	0.00	0.334	0.319	0.199	0.212	0.374	0.512
Hieracium sabaudum	1	0.00	0.311	0.296	0.193	0.185	0.353	0.444
Hohenbergia stellata	2	14.03	0.101	0.097	0.050	0.095	0.134	0.262
Holosteum umbellatum	1	0.00	0.405	0.397	0.190	0.297	0.498	0.818
Hylotelephium maximum	1	0.00	0.397	0.388	0.181	0.227	0.448	0.623
Hyoscyamus aureus	1, 2	6.00	0.329	0.316	0.215	0.223	0.449	0.597
Hypoxis decumbens	1	0.00	0.333	0.315	0.196	0.237	0.375	0.570
Impatiens walleriana	2	30.40	0.157	0.145	0.025	0.044	0.066	0.121
Ipomoea purpurea	2	38.87	0.144	0.144	0.106	0.168	0.283	0.430
Ipomoea squamosa	1	0.00	0.198	0.197	0.114	0.179	0.302	0.457
Isatis lusitanica	1	0.00	0.331	0.318	0.195	0.194	0.360	0.464
Ixora coccinea	2	41.48	0.298	0.285	0.153	0.191	0.373	0.512

Jacobaea vulgaris	1	0.00	0.306	0.290	0.196	0.185	0.359	0.433
Jacquemontia sp.	2	23.00	0.146	0.150	0.181	0.257	0.442	0.585
Justicia brandegeeana	1	0.00	0.237	0.217	0.132	0.134	0.250	0.324
Justicia gendarussa	1, 2	11.84	0.151	0.148	0.084	0.111	0.223	0.305
Knautia arvensis	1	0.00	0.277	0.272	0.137	0.202	0.364	0.557
Knautia dipsacifolia	1	0.00	0.263	0.258	0.138	0.200	0.368	0.550
Kopsia fruticosa	2	40.12	0.209	0.204	0.101	0.186	0.271	0.476
Lamium album	1, 2	11.00	0.409	0.400	0.226	0.287	0.560	0.789
Lamium galeobdolon	1, 2	6.00	0.357	0.343	0.163	0.163	0.391	0.436
Lamium garganicum	1, 2	12.00	0.312	0.306	0.128	0.235	0.342	0.648
Lantana camara	2	9.87	0.213	0.197	0.153	0.147	0.367	0.392
Lapsana communis	1	0.00	0.335	0.319	0.212	0.204	0.438	0.547
Lathyrus blepharicarpus	1, 2	7.00	0.134	0.125	0.138	0.205	0.268	0.469
Lathyrus gorgoni	1, 2	9.00	0.174	0.162	0.139	0.204	0.272	0.466
Lathyrus pratensis	1, 2	6.00	0.291	0.274	0.190	0.182	0.397	0.486
Lathyrus vernus	1, 2	10.00	0.188	0.186	0.085	0.136	0.228	0.375
Launaea angustifolia	1, 2	2.00	0.290	0.273	0.194	0.179	0.400	0.419
Launaea mucronata	1, 2	2.00	0.309	0.295	0.181	0.196	0.342	0.474
Launaea nudicaulis	1, 2	2.00	0.274	0.257	0.197	0.189	0.412	0.507
Lebeckia halenbergensis	1	0.00	0.260	0.245	0.181	0.165	0.427	0.442
Leontodon laciniatus	1, 2	2.00	0.279	0.261	0.197	0.183	0.408	0.491
Leontodon tuberosus	1, 2	2.00	0.318	0.304	0.197	0.200	0.363	0.480
Leopoldia comosa	1, 2	10.00	0.149	0.148	0.117	0.142	0.302	0.363
Leopoldia longipes	1, 2	10.00	0.272	0.262	0.116	0.139	0.293	0.373
Lepidium draba	1	0.00	0.423	0.415	0.208	0.305	0.527	0.840
Liatris spicata	1	0.00	0.150	0.152	0.128	0.191	0.335	0.490
Limodorum abortivum	1, 2	14.00	0.192	0.189	0.054	0.152	0.143	0.389
Linaria vulgaris	1, 2	17.00	0.339	0.330	0.183	0.224	0.448	0.601
Linum pubescens	1, 2	3.00	0.110	0.106	0.163	0.201	0.303	0.415
Lobelia anceps	1, 2	5.38	0.252	0.249	0.153	0.209	0.406	0.576
Lotus longesiliquosus	1, 2	5.00	0.241	0.225	0.161	0.153	0.387	0.408
Lycium shawii	1, 2	8.00	0.312	0.303	0.152	0.202	0.381	0.556
Malouetia arborea	2	10.76	0.415	0.406	0.232	0.300	0.567	0.825
Malvaviscus arboreus	2	58.04	0.133	0.124	0.059	0.080	0.156	0.220
Mammillaria bombycina	2	11.25	0.207	0.205	0.123	0.195	0.327	0.498
Mammillaria elongata	2	10.22	0.369	0.358	0.206	0.249	0.495	0.666

Maranta leuconeura	1, 2	9.63	0.371	0.363	0.197	0.273	0.494	0.751
Matricaria aurea	1	0.00	0.271	0.254	0.183	0.182	0.432	0.486
Megaskepasma erythrochlamys	2	19.11	0.068	0.067	0.041	0.053	0.110	0.146
Melampyrum pratense	1, 2	8.00	0.290	0.275	0.173	0.181	0.415	0.484
Melampyrum sylvaticum	1, 2	4.00	0.265	0.250	0.190	0.183	0.397	0.489
Mentha aquatica	1	0.00	0.315	0.311	0.160	0.248	0.427	0.683
Merremia dissecta	2	18.45	0.424	0.416	0.129	0.262	0.341	0.722
Mesembryanthemum cryptanthum	1, 2	4.00	0.400	0.390	0.202	0.252	0.501	0.693
Mesembryanthemum nodiflorum	1, 2	2.00	0.398	0.387	0.192	0.234	0.468	0.643
Moehringia trinervia	1	0.00	0.425	0.416	0.207	0.274	0.519	0.753
Moricandia nitens	1, 2	8.00	0.236	0.234	0.088	0.144	0.227	0.368
Mucuna bennettii	2	70.36	0.169	0.161	0.070	0.079	0.182	0.211
Musa ornata	2	40.13	0.200	0.202	0.149	0.208	0.400	0.532
Mussaenda philippica	2	24.44	0.311	0.296	0.230	0.228	0.470	0.611
Myosotis alpestris	1, 2	4.00	0.256	0.256	0.152	0.230	0.407	0.590
Myosotis decumbens	1, 2	2.00	0.345	0.340	0.171	0.245	0.451	0.674
Myosotis stricta	1, 2	2.00	0.278	0.276	0.119	0.219	0.317	0.561
Myosotis vestergrenii	1, 2	4.00	0.233	0.232	0.155	0.230	0.416	0.590
Nanorrhinum scoparium	1, 2	17.00	0.368	0.358	0.189	0.218	0.454	0.583
Neomarica candida	2	1.00	0.426	0.417	0.222	0.332	0.564	0.915
Neoregelia cruenta	1, 2	23.28	0.298	0.290	0.104	0.178	0.276	0.489
Nopalea cochenillifera	2	32.48	0.127	0.124	0.018	0.060	0.048	0.165
Ocimum basilicum	2	4.02	0.392	0.386	0.149	0.309	0.393	0.851
Odontonema tubaeforme	2	29.00	0.105	0.103	0.028	0.082	0.075	0.226
Onobrychis crista-galli	1, 2	4.00	0.222	0.220	0.127	0.204	0.339	0.522
Ononis natrix	1, 2	7.00	0.298	0.281	0.205	0.193	0.420	0.516
Orchis italica	1, 2	10.00	0.280	0.275	0.152	0.223	0.404	0.614
Origanum vulgare	1, 2	5.00	0.198	0.193	0.046	0.068	0.123	0.186
Oxalis debilis	2	13.09	0.122	0.127	0.134	0.190	0.351	0.485
Oxytropis jacquinii	1, 2	10.00	0.175	0.173	0.096	0.162	0.256	0.415
Oxytropis neglecta	1, 2	12.00	0.250	0.248	0.104	0.158	0.270	0.404
Pachystachys lutea	2	29.81	0.278	0.258	0.174	0.164	0.411	0.439
Pachystachys spicata	2	28.39	0.043	0.043	0.022	0.024	0.058	0.066
Papaver hybridum	1	0.00	0.036	0.035	0.067	0.103	0.180	0.236
Papaver rhoeas	1	0.00	0.058	0.056	0.071	0.110	0.190	0.260
Papaver umbonatum	1	0.00	0.045	0.044	0.036	0.060	0.098	0.142

Parnassia palustris	1	0.00	0.405	0.396	0.218	0.277	0.548	0.763
Pavonia alnifolia	2	1.00	0.200	0.193	0.063	0.112	0.167	0.309
Pedicularis comosa	1, 2	14.00	0.319	0.309	0.167	0.201	0.415	0.538
Pedicularis recutita	1, 2	12.00	0.185	0.179	0.053	0.107	0.142	0.295
Phalaenopsis amabilis	2	34.22	0.391	0.382	0.137	0.285	0.360	0.785
Phlomoides laciniata	1, 2	13.00	0.369	0.359	0.177	0.248	0.439	0.681
Phyllodoce caerulea	1	0.00	0.263	0.258	0.138	0.200	0.368	0.550
Phyteuma betonicifolium	1, 2	16.00	0.064	0.065	0.109	0.117	0.281	0.300
Phyteuma hedraianthifolium	1	0.00	0.198	0.196	0.105	0.173	0.274	0.442
Phyteuma hemisphaericum	1	0.00	0.176	0.173	0.056	0.125	0.149	0.343
Phyteuma nigrum	1, 2	12.00	0.091	0.089	0.032	0.028	0.085	0.077
Picris longirostris	1, 2	2.00	0.314	0.299	0.222	0.212	0.454	0.567
Pilosella officinarum	1	0.00	0.324	0.309	0.197	0.204	0.369	0.494
Pinguicula alpina	1, 2	7.00	0.406	0.397	0.220	0.281	0.538	0.773
Pitcairnia staminea	2	19.42	0.139	0.130	0.061	0.080	0.163	0.221
Platanthera clavellata	1, 2	10.00	0.094	0.093	0.075	0.096	0.201	0.264
Potentilla heptaphylla	1	0.00	0.312	0.298	0.197	0.191	0.361	0.459
Potentilla reptans	1	0.00	0.287	0.274	0.170	0.171	0.319	0.411
Premna cordifolia	2	2.56	0.314	0.304	0.100	0.102	0.252	0.272
Primula elatior	1, 2	11.00	0.318	0.302	0.229	0.226	0.469	0.604
Prunus padus	1	0.00	0.396	0.388	0.218	0.298	0.547	0.820
Pseudolaelia corcovadensis	1, 2	2.00	0.283	0.280	0.131	0.263	0.352	0.674
Pulicaria incisa	1	0.00	0.327	0.311	0.237	0.231	0.479	0.618
Pulmonaria mollis	1, 2	10.00	0.082	0.085	0.160	0.154	0.308	0.332
Pulmonaria obscura	1, 2	10.00	0.098	0.101	0.181	0.169	0.348	0.364
Quesnelia arvensis	1, 2	20.21	0.210	0.207	0.122	0.202	0.326	0.518
Quesnelia liboniana	1, 2	44.33	0.067	0.066	0.029	0.025	0.077	0.070
Quesnelia sp.	2	20.21	0.185	0.182	0.043	0.055	0.114	0.151
Randia sp.	2	38.00	0.420	0.412	0.204	0.334	0.528	0.919
Ranunculus acris	1	0.00	0.290	0.272	0.195	0.180	0.404	0.420
Ranunculus asiaticus	1	0.00	0.107	0.107	0.077	0.131	0.204	0.336
Ranunculus marginatus	1	0.00	0.228	0.217	0.132	0.136	0.326	0.327
Ranunculus millefolius	1	0.00	0.320	0.307	0.189	0.211	0.356	0.508
Renanthera coccinea	2	4.94	0.008	0.008	0.005	0.005	0.013	0.014
Retama raetam	1, 2	6.00	0.391	0.383	0.183	0.297	0.479	0.819
Rhagadiolus stellatus	1, 2	2.00	0.323	0.306	0.218	0.203	0.442	0.475

Rivina humilis	1, 2	2.00	0.317	0.309	0.170	0.229	0.442	0.629
Russelia equisetiformis	2	21.77	0.077	0.073	0.044	0.048	0.118	0.132
Ruta chalepensis	1	0.00	0.274	0.257	0.194	0.186	0.405	0.498
Salvia dominica	1, 2	8.00	0.396	0.387	0.188	0.301	0.488	0.827
Salvia fruticosa	1, 2	4.00	0.298	0.293	0.127	0.246	0.340	0.630
Salvia hierosolymitana	1, 2	10.00	0.276	0.271	0.146	0.196	0.387	0.540
Salvia lanigera	1, 2	11.00	0.158	0.157	0.120	0.187	0.315	0.477
Salvia splendens	2	40.40	0.059	0.058	0.032	0.035	0.085	0.097
Sanchezia oblonga	1	0.00	0.088	0.084	0.035	0.038	0.093	0.091
Sanchezia speciosa	1, 2	44.99	0.261	0.246	0.164	0.170	0.393	0.454
Sansevieria cylindrica	2	25.91	0.350	0.339	0.231	0.238	0.482	0.637
Saraca thaipingensis	2	14.49	0.292	0.275	0.121	0.133	0.236	0.320
Satureja thymbra	1, 2	5.00	0.243	0.238	0.101	0.185	0.271	0.510
Scandix pecten-veneris	1	0.00	0.337	0.328	0.170	0.234	0.433	0.644
Scilla hyacinthoides	1, 2	1.00	0.290	0.287	0.139	0.270	0.372	0.691
Scorzonera papposa	1, 2	2.00	0.252	0.248	0.077	0.190	0.206	0.487
Scorzoneroides autumnalis	1	0.00	0.333	0.318	0.191	0.189	0.398	0.507
Scrophularia xanthoglossa	1, 2	1.00	0.146	0.140	0.065	0.082	0.172	0.226
Securigera varia	1, 2	8.00	0.397	0.389	0.204	0.287	0.519	0.789
Senecio glaucus	1	0.00	0.282	0.266	0.196	0.188	0.410	0.502
Senecio vernalis	1	0.00	0.324	0.308	0.208	0.209	0.434	0.560
Silene acaulis	1, 2	5.00	0.257	0.254	0.147	0.221	0.395	0.566
Silene aegyptiaca	1, 2	15.00	0.276	0.270	0.091	0.226	0.243	0.579
Silene flos-cuculi	1, 2	9.00	0.223	0.218	0.093	0.187	0.247	0.479
Silene nutans	1, 2	11.00	0.401	0.392	0.195	0.301	0.506	0.829
Sinapis arvensis	1	0.00	0.332	0.319	0.190	0.234	0.364	0.563
Sinapis incana	1, 2	3.00	0.338	0.325	0.191	0.222	0.361	0.536
Smilax sp.	2	4.22	0.313	0.301	0.187	0.207	0.441	0.553
Sobralia yauaperyensis	2	42.43	0.392	0.385	0.198	0.317	0.518	0.873
Solidago canadensis	1	0.00	0.303	0.285	0.204	0.191	0.421	0.512
Sonchus oleraceus	1, 2	2.00	0.302	0.292	0.155	0.184	0.293	0.444
Spathoglottis unguiculata	2	1.00	0.061	0.062	0.097	0.127	0.253	0.325
Stachys recta	1, 2	8.00	0.383	0.374	0.212	0.245	0.509	0.656
Stachys sylvatica	1, 2	9.00	0.134	0.131	0.026	0.060	0.069	0.164
Stachytarpheta speciosa	2	38.89	0.179	0.181	0.127	0.187	0.338	0.479
Stellaria holostea	1	0.00	0.428	0.420	0.162	0.335	0.429	0.921

Stellaria palustris	1	0.00	0.423	0.414	0.176	0.302	0.463	0.830
Stifftia chrysantha	2	24.88	0.242	0.230	0.131	0.147	0.324	0.395
Stromanthe sp.	2	7.47	0.125	0.121	0.069	0.089	0.183	0.245
Swartzia simplex	1	0.00	0.273	0.252	0.163	0.204	0.319	0.483
Symphoricarpos albus	1	0.00	0.248	0.243	0.089	0.150	0.239	0.412
Symphytum brachycalyx	1, 2	1.00	0.406	0.397	0.222	0.295	0.556	0.813
Syzygium malaccense	2	28.32	0.150	0.148	0.079	0.079	0.203	0.203
Tacinga palmadora	2	9.79	0.140	0.134	0.088	0.089	0.229	0.237
Tamarix nilotica	1	0.00	0.370	0.361	0.174	0.239	0.443	0.656
Tanaecium pyramidatum	2	39.45	0.261	0.258	0.134	0.239	0.360	0.613
Taraxacum officinale	1	0.00	0.228	0.211	0.157	0.146	0.377	0.390
Thalia geniculata	2	7.07	0.311	0.304	0.157	0.226	0.414	0.622
Thunbergia erecta	2	52.21	0.113	0.114	0.152	0.203	0.382	0.462
Thunbergia mysorensis	2	69.04	0.303	0.287	0.116	0.157	0.287	0.419
Thymus serpyllum	1, 2	3.00	0.184	0.182	0.097	0.143	0.260	0.393
Tillandsia polystachia	1, 2	30.24	0.235	0.229	0.121	0.165	0.317	0.454
Tillandsia stricta	2	13.66	0.158	0.156	0.103	0.151	0.276	0.414
Tillandsia tenuifolia	2	25.74	0.190	0.193	0.157	0.242	0.412	0.620
Tridax procumbens	1	0.00	0.416	0.408	0.253	0.293	0.607	0.784
Trifolium clypeatum	1, 2	8.50	0.397	0.389	0.226	0.285	0.569	0.785
Trifolium repens	1, 2	3.00	0.403	0.395	0.192	0.238	0.482	0.655
Trifolium resupinatum	1, 2	3.00	0.188	0.186	0.101	0.153	0.270	0.421
Trifolium stellatum	1, 2	7.50	0.219	0.213	0.114	0.150	0.303	0.413
Trigonella caelesyriaca	1, 2	3.00	0.328	0.312	0.238	0.226	0.481	0.517
Trigonella kotschyi	1, 2	3.00	0.318	0.302	0.221	0.211	0.447	0.483
Tripleurospermum auriculatum	1	0.00	0.277	0.260	0.189	0.186	0.395	0.498
Tussilago farfara	1	0.00	0.312	0.296	0.188	0.200	0.444	0.535
Urospermum picroides	1, 2	2.00	0.350	0.338	0.248	0.253	0.506	0.676
Ursinia cakilefolia	1	0.00	0.168	0.152	0.101	0.145	0.196	0.344
Vaccinium vitis-idaea	1	0.00	0.377	0.368	0.207	0.242	0.514	0.665
Vellozia candida	2	47.34	0.422	0.414	0.219	0.326	0.555	0.898
Verbascum densiflorum	1	0.00	0.343	0.329	0.202	0.217	0.378	0.524
Verbascum lychnitis	1, 2	2.00	0.313	0.299	0.195	0.187	0.357	0.438
Veronica arvensis	1, 2	2.00	0.347	0.341	0.152	0.241	0.404	0.663
Veronica chamaedrys	1, 2	2.00	0.164	0.165	0.148	0.197	0.369	0.505
Veronica prostrata	1, 2	1.00	0.218	0.218	0.119	0.155	0.303	0.396

Veronica spicata	1, 2	2.00	0.158	0.156	0.082	0.124	0.216	0.316
Viburnum opulus	1, 2	1.00	0.406	0.398	0.212	0.290	0.537	0.797
Vicia hybrida	1	0.00	0.348	0.336	0.224	0.231	0.473	0.617
Vicia sativa	1, 2	6.00	0.208	0.204	0.117	0.168	0.313	0.463
Vigna unguiculata	1, 2	16.39	0.188	0.188	0.132	0.182	0.352	0.466
Vincetoxicum hirundinaria	1, 2	2.00	0.402	0.393	0.173	0.191	0.433	0.526
Vriesea neoglutinosa	1, 2	31.22	0.028	0.029	0.016	0.019	0.044	0.052
Youngia japonica	2	4.24	0.345	0.333	0.157	0.151	0.378	0.362
Yucca aloifolia	2	1.00	0.422	0.414	0.185	0.306	0.479	0.842
Zilla spinosa	1, 2	9.00	0.283	0.279	0.124	0.245	0.332	0.628

Table S5. K statistics used for testing phylogenetic signal using Blomberg's K statistic (Blomberg et al., 2003) in the full dataset ($N = 389$ species). $K < 1$ indicates a given trait is not more similar than expected by chance while accounting for phylogenetic relatedness. The different scenarios indicate different choices as to how to insert the branches not found in the PhytoPhylo megaphylogeny [Qian and Jin (2016) modified from Zanne et al. (2014)], which generated three distinct trees.

**Trait**	Blomberg's K		
	**Tree I**	**Tree II**	**Tree III**
ACB_{Apis}	0.060	0.005	0.048
ACB_{Bombus}	0.061	0.005	0.049
CCB_{Apis}	0.054	0.009	0.042
CCB_{Bombus}	0.058	0.003	0.044
SP_{Apis}	0.049	0.002	0.037
SP_{Bombus}	0.057	0.002	0.044
$Depth$	0.087	0.008	0.059

Table S6. ANOVA tests using contrast as the response variable and pollination system as the explanatory one. Results for achromatic (ACB) and chromatic (CCB) contrasts against the background and spectral purity (SP) are given. Bold values indicate a significant effect of pollination system under $p < 0.05$.

**Response**	**Explanatory**	**DF**	**SSE**	**MSE**	**F**	**p**
ACB_{Apis}	$System$	3	0.348	0.116	12.252	**< 0.001**
ACB_{Bombus}		3	0.333	0.111	12.444	**< 0.001**
CCB_{Apis}		3	0.199	0.066	23.833	**< 0.001**
CCB_{Bombus}		3	0.252	0.084	21.620	**< 0.001**
SP_{Apis}		3	0.826	0.275	19.954	**< 0.001**
SP_{Bombus}		3	1.643	0.548	17.750	**< 0.001**

Table S7. Tukey's HSD results for mean comparisons in bee contrasts found between different pollination systems. Results for achromatic (textbfACB) and chromatic (textbfCCB) contrasts against the background and spectral purity (textbfSP) are given. Bold values indicate significant differences under $p < 0.05$.

Contrast	Comparison	β	Confidence Interval	p
ACB_{Apis}	bee+insect – bee	0.016	$-0.018, 0.050$	0.614
	insect – bee	-0.013	$-0.067, 0.041$	0.928
	bird – bee	-0.103	$-0.152, -0.053$	$< \mathbf{0.001}$
	insect– bee+insect	-0.029	$-0.085, 0.027$	0.543
	bird – bee+insect	-0.119	$-0.170, -0.067$	$< \mathbf{0.001}$
	bird – insect	-0.090	$-0.156, -0.023$	0.003
ACB_{Bombus}	bee+insect – bee	0.013	$-0.020, 0.047$	0.723
	insect – bee	-0.012	$-0.064, 0.041$	0.939
	bird – bee	-0.102	$-0.150, -0.054$	$< \mathbf{0.001}$
	insect – bee+insect	-0.025	$-0.079, 0.029$	0.633
	bird – bee+insect	-0.115	$-0.166, -0.065$	$< \mathbf{0.001}$
	bird – insect	-0.090	$-0.155, -0.026$	0.002
CCB_{Apis}	bee+insect – bee	0.021	$0.003, 0.040$	0.016
	insect – bee	-0.005	$-0.034, 0.024$	0.975
	bird – bee	-0.070	$-0.097, -0.044$	$< \mathbf{0.001}$
	insect – bee+insect	-0.026	$-0.057, 0.004$	0.119
	bird – bee+insect	-0.092	$-0.120, -0.064$	$< \mathbf{0.001}$
	bird – insect	-0.066	$-0.102, -0.030$	$< \mathbf{0.001}$
CCB_{Bombus}	bee+insect – bee	0.004	$-0.018, 0.026$	0.961
	insect – bee	-0.010	$-0.045, 0.024$	0.866
	bird – bee	-0.093	$-0.125, -0.061$	$< \mathbf{0.001}$
	insect – bee+insect	-0.014	$-0.050, 0.021$	0.724
	bird – bee+insect	-0.097	$-0.130, -0.064$	$< \mathbf{0.001}$
	bird – insect	-0.083	$-0.125, -0.040$	$< \mathbf{0.001}$
SP_{Apis}	bee+insect – bee	0.022	$-0.019, 0.063$	0.512
	insect – bee	-0.014	$-0.079, 0.051$	0.943
	bird – bee	-0.160	$-0.220, -0.101$	$< \mathbf{0.001}$
	insect – bee+insect	-0.036	$-0.104, 0.031$	0.512
	bird – bee+insect	-0.182	$-0.245, -0.120$	$< \mathbf{0.001}$
	bird – insect	-0.146	$-0.226, -0.066$	$< \mathbf{0.001}$
SP_{Bombus}	bee+insect – bee	0.000	$-0.062, 0.061$	0.999
	insect – bee	-0.031	$-0.128, 0.066$	0.846
	bird – bee	-0.242	$-0.331, -0.153$	$< \mathbf{0.001}$
	insect – bee+insect	-0.031	$-0.132, 0.071$	0.863
	bird – bee+insect	-0.242	$-0.335, -0.148$	$< \mathbf{0.001}$
	bird – insect	-0.211	$-0.331, -0.092$	$< \mathbf{0.001}$

Table S8. Tukey's HSD results for mean comparisons in bee contrasts found between different flower color categories. Results for achromatic (**ACB**) and chromatic (**CCB**) contrasts against the background and spectral purity (**SP**) are given, considering only colors with $N > 30$ species. Bold values indicate significant differences between means under $p < 0.05$.

Contrast	Comparison	β	Confidence Interval	p
ACB_{Apis}	UV-Yellow – UV-White	-0.072	$-0.091, < 0.001$	$< \mathbf{0.001}$
	UV+Yellow – UV-White	-0.052	$-0.075, < 0.001$	$< \mathbf{0.001}$
	UV-Pink – UV-White	-0.168	$-0.192, < 0.001$	$< \mathbf{0.001}$
	UV-Red – UV-White	-0.256	$-0.276, < 0.001$	$< \mathbf{0.001}$
	UV+Yellow – UV-Yellow	0.020	$-0.004, 0.163$	0.163
	UV-Pink – UV-Yellow	-0.096	$-0.122, < 0.001$	$< \mathbf{0.001}$
	UV-Red – UV-Yellow	-0.184	$-0.206, < 0.001$	$< \mathbf{0.001}$
	UV-Pink – UV+Yellow	-0.116	$-0.145, < 0.001$	$< \mathbf{0.001}$
	UV-Red – UV+Yellow	-0.204	$-0.229, < 0.001$	$< \mathbf{0.001}$
	UV-Red – UV-Pink	-0.088	$-0.115, < 0.001$	$< \mathbf{0.001}$
ACB_{Bombus}	UV-Yellow – UV-White	-0.080	$-0.099, < 0.001$	$< \mathbf{0.001}$
	UV+Yellow – UV-White	-0.059	$-0.082, < 0.001$	$< \mathbf{0.001}$
	UV-Pink – UV-White	-0.162	$-0.186, < 0.001$	$< \mathbf{0.001}$
	UV-Red – UV-White	-0.253	$-0.272, < 0.001$	$< \mathbf{0.001}$
	UV+Yellow – UV-Yellow	0.021	$-0.004, 0.133$	0.133
	UV-Pink – UV-Yellow	-0.082	$-0.108, < 0.001$	$< \mathbf{0.001}$
	UV-Red – UV-Yellow	-0.173	$-0.194, < 0.001$	$< \mathbf{0.001}$
	UV-Pink – UV+Yellow	-0.103	$-0.131, < 0.001$	$< \mathbf{0.001}$
	UV-Red – UV+Yellow	-0.193	$-0.218, < 0.001$	$< \mathbf{0.001}$
	UV-Red – UV-Pink	-0.091	$-0.117, < 0.001$	$< \mathbf{0.001}$
CCB_{Apis}	UV-Yellow – UV-White	0.006	$-0.008, 0.749$	0.749
	UV+Yellow – UV-White	-0.007	$-0.023, 0.779$	0.779
	UV-Pink – UV-White	-0.068	$-0.086, < 0.001$	$< \mathbf{0.001}$
	UV-Red – UV-White	-0.132	$-0.147, < 0.001$	$< \mathbf{0.001}$
	UV+Yellow – UV-Yellow	-0.013	$-0.031, 0.261$	0.261
	UV-Pink – UV-Yellow	-0.074	$-0.093, < 0.001$	$< \mathbf{0.001}$
	UV-Red – UV-Yellow	-0.138	$-0.154, < 0.001$	$< \mathbf{0.001}$
	UV-Pink – UV+Yellow	-0.061	$-0.082, < 0.001$	$< \mathbf{0.001}$
	UV-Red – UV+Yellow	-0.125	$-0.144, < 0.001$	$< \mathbf{0.001}$
	UV-Red – UV-Pink	-0.064	$-0.083, < 0.001$	$< \mathbf{0.001}$
CCB_{Bombus}	UV-Yellow – UV-White	-0.070	$-0.085, < 0.001$	$< \mathbf{0.001}$
	UV+Yellow – UV-White	-0.069	$-0.087, < 0.001$	$< \mathbf{0.001}$
	UV-Pink – UV-White	-0.081	$-0.101, < 0.001$	$< \mathbf{0.001}$
	UV-Red – UV-White	-0.192	$-0.208, < 0.001$	$< \mathbf{0.001}$
	UV+Yellow – UV-Yellow	0.001	$-0.019, 1.000$	1.000
	UV-Pink – UV-Yellow	-0.011	$-0.032, 0.574$	0.574
	UV-Red – UV-Yellow	-0.123	$-0.140, < 0.001$	$< \mathbf{0.001}$
	UV-Pink – UV+Yellow	-0.012	$-0.036, 0.581$	0.581
	UV-Red – UV+Yellow	-0.124	$-0.144, < 0.001$	$< \mathbf{0.001}$
	UV-Red – UV-Pink	-0.111	$-0.133, < 0.001$	$< \mathbf{0.001}$
SP_{Apis}	UV-Yellow – UV-White	-0.059	$-0.088, -0.030$	$< \mathbf{0.001}$
	UV+Yellow – UV-White	-0.127	$-0.162, -0.093$	$< \mathbf{0.001}$
	UV-Pink – UV-White	-0.158	$-0.196, -0.121$	$< \mathbf{0.001}$
	UV-Red – UV-White	-0.332	$-0.362, -0.302$	$< \mathbf{0.001}$
	UV+Yellow – UV-Yellow	-0.069	$-0.106, -0.032$	$< \mathbf{0.001}$
	UV-Pink – UV-Yellow	-0.100	$-0.139, -0.060$	$< \mathbf{0.001}$
	UV-Red – UV-Yellow	-0.273	$-0.306, -0.240$	$< \mathbf{0.001}$
	UV-Pink – UV+Yellow	-0.031	$-0.075, 0.013$	0.295
	UV-Red – UV+Yellow	-0.205	$-0.243, -0.166$	$< \mathbf{0.001}$
	UV-Red – UV-Pink	-0.174	$-0.214, -0.133$	$< \mathbf{0.001}$
SP_{Bombus}	UV-Yellow – UV-White	-0.208	$-0.249, -0.167$	$< \mathbf{0.001}$
	UV+Yellow – UV-White	-0.248	$-0.297, -0.200$	$< \mathbf{0.001}$
	UV-Pink – UV-White	-0.239	$-0.291, -0.187$	$< \mathbf{0.001}$
	UV-Red – UV-White	-0.527	$-0.570, -0.484$	$< \mathbf{0.001}$
	UV+Yellow – UV-Yellow	-0.041	$-0.093, 0.012$	0.209
	UV-Pink – UV-Yellow	-0.031	$-0.086, 0.025$	0.545
	UV-Red – UV-Yellow	-0.319	$-0.366, -0.273$	$< \mathbf{0.001}$
	UV-Pink – UV+Yellow	0.010	$-0.052, 0.071$	0.992
	UV-Red – UV+Yellow	-0.279	$-0.332, -0.225$	$< \mathbf{0.001}$
	UV-Red – UV-Pink	-0.288	$-0.345, -0.232$	$< \mathbf{0.001}$

Table S9. Regression models using contrasts as the response variable achromatic and flower depth as the explanatory variable. Results for achromatic (**ACB**) and chromatic (**CCB**) contrasts against the background and spectral purity (**SP**) are given. For the regressions, data of species for which $flower\ depth \neq 0$ mm regardless of pollination system were used (286 species). Bold values indicate significant associations under $p < 0.05$.

Response	Explanatory	β	SE	R^2	p
ACB_{Apis}	$log(depth)$	-0.029	0.006	0.087	$< \mathbf{0.001}$
ACB_{Bombus}		-0.029	0.005	0.087	$< \mathbf{0.001}$
CCB_{Apis}		-0.021	0.003	0.148	$< \mathbf{0.001}$
CCB_{Bombus}		-0.020	0.004	0.081	$< \mathbf{0.001}$
SP_{Apis}		-0.044	0.007	0.121	$< \mathbf{0.001}$
SP_{Bombus}		-0.053	0.011	0.073	$< \mathbf{0.001}$
