# Supplementary figures and images for: Flower Conspicuousness to Bees Across Pollination Systems: A Generalized Test of the Bee-Avoidance Hypothesis
Source: Front Plant Sci. 2020 Sep 24;11:558684. doi: 10.3389/fpls.2020.558684 (PMC7542005; doi:10.3389/fpls.2020.558684)

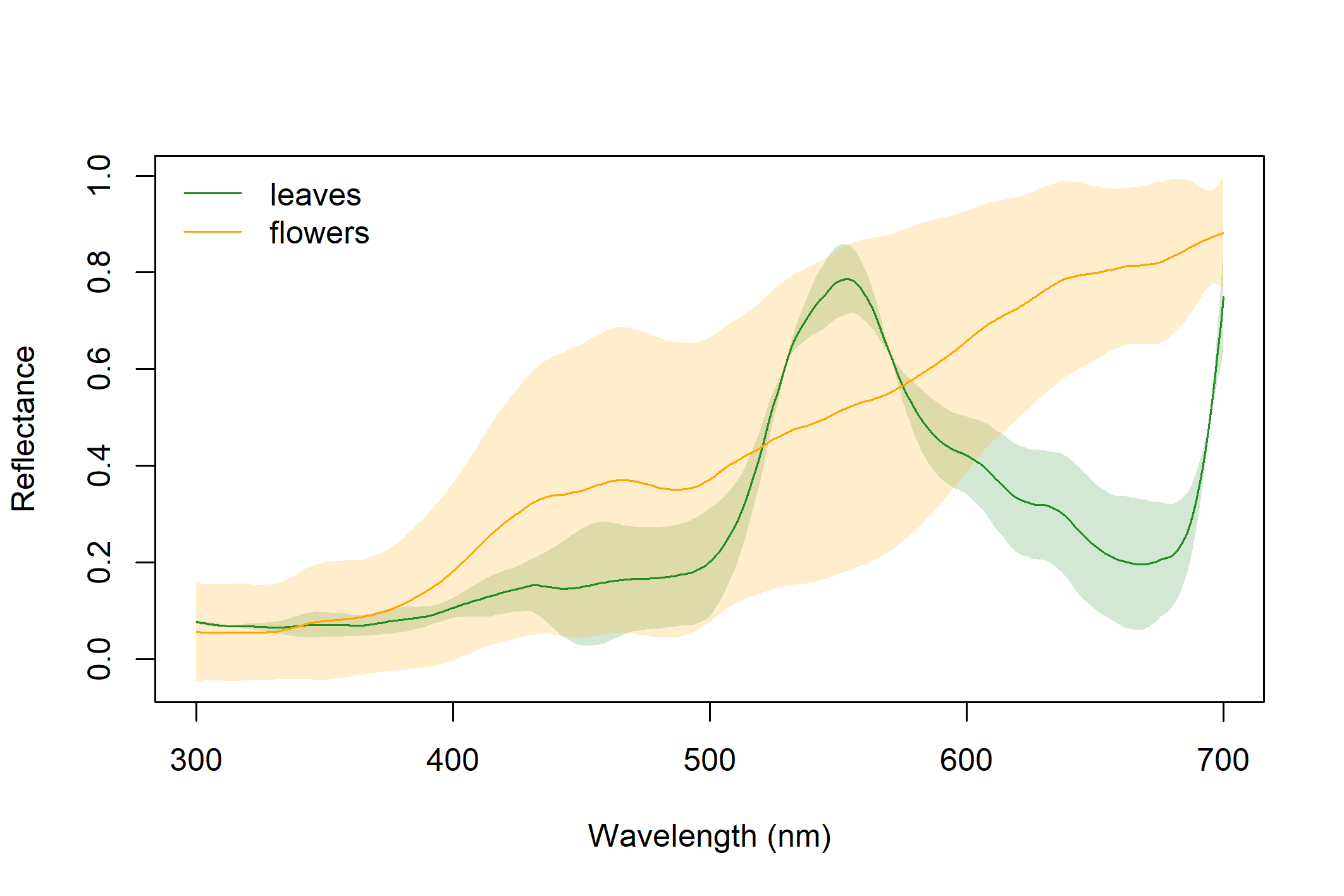

Supplement: Supplementary file 3 [file Image_1.tiff]

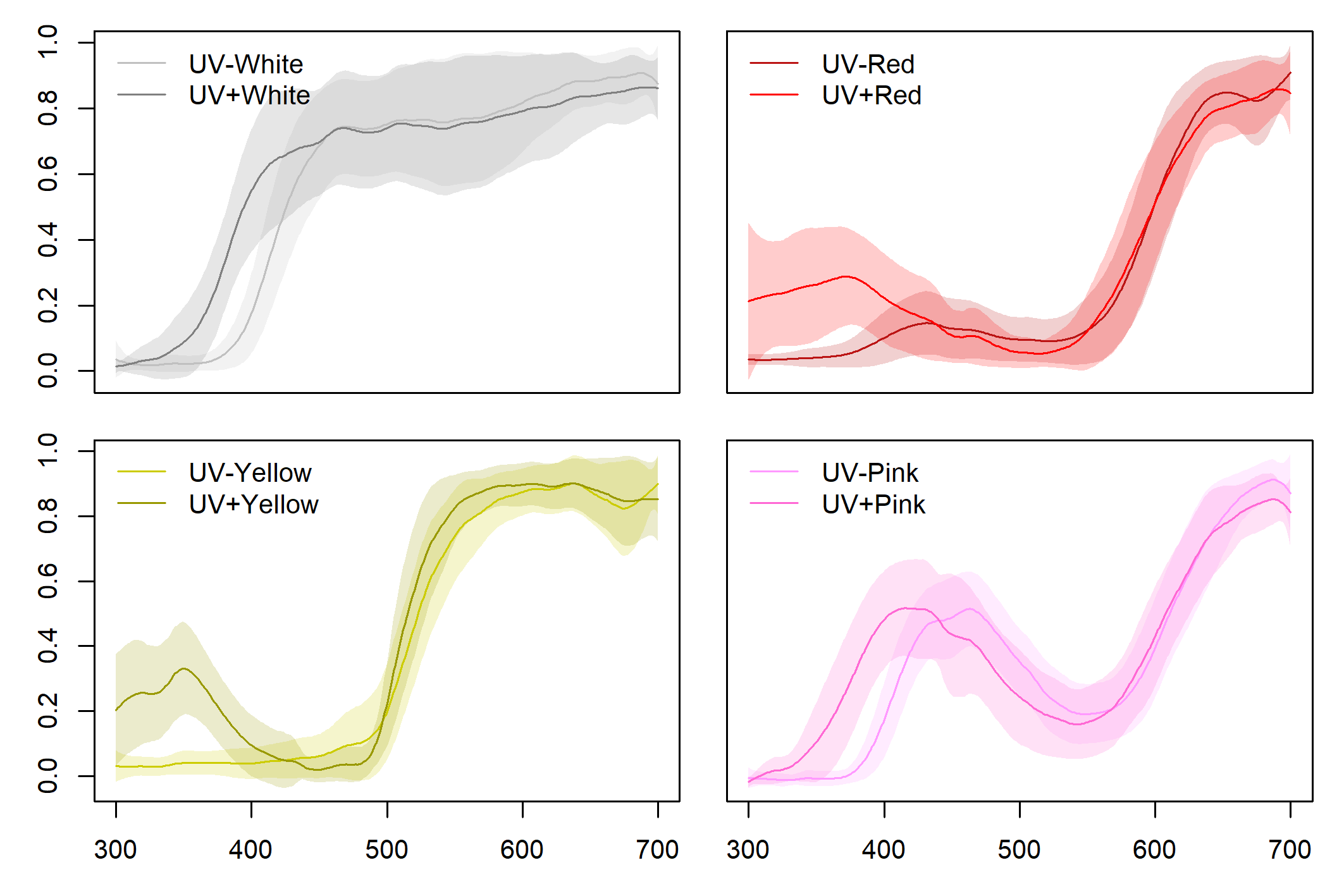

Supplement: Supplementary file 4 [file Image_2.tiff]

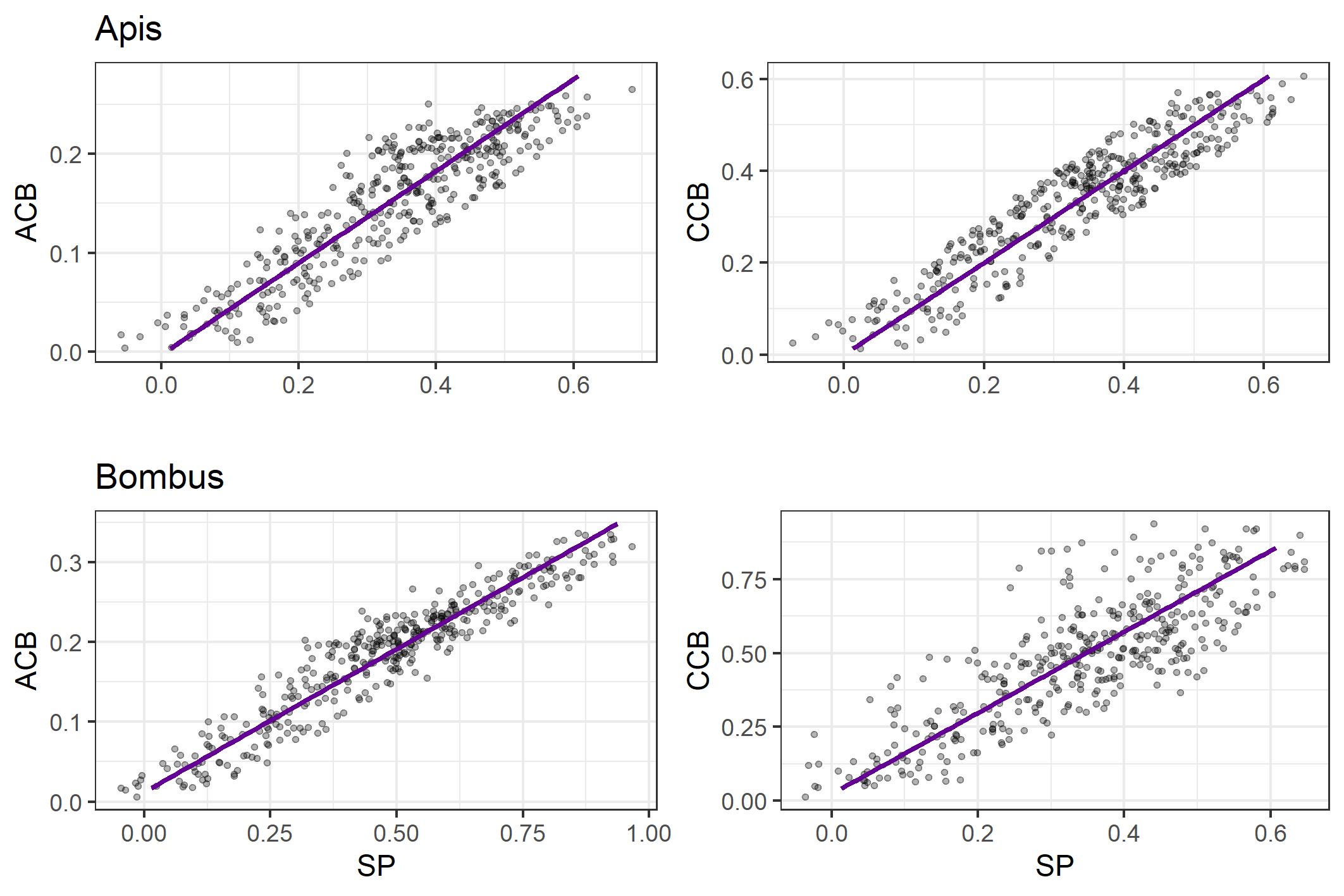

Supplement: Supplementary file 5 [file Image_3.tiff]
